# Sustainable Construction of Heterocyclic 1,2,3-Triazoles by Strict Click [3+2] Cycloaddition Reactions Between Azides and Alkynes on Copper/Carbon in Water

**DOI:** 10.3389/fchem.2019.00081

**Published:** 2019-02-19

**Authors:** Noura Aflak, Hicham Ben El Ayouchia, Lahoucine Bahsis, El Mountassir El Mouchtari, Miguel Julve, Salah Rafqah, Hafid Anane, Salah-Eddine Stiriba

**Affiliations:** ^1^Laboratoire de Chimie Analytique et Moléculaire, LCAM, Faculté Polydisciplinaire de Safi, Université Cadi Ayyad, Safi, Morocco; ^2^Instituto de Ciencia Molecular/ICMol, Universidad de Valencia, Valencia, Spain

**Keywords:** copper, activated carbon, 1, 2, 3-triazole, click chemistry, heterogeneous catalyst, recovery/recycling, water

## Abstract

1,4-Disubstituted-1,2,3-triazoles, considered as an important and useful class of heterocycles with potential applications in material science and biology, have been prepared in an efficient and selective manner by copper on carbon-catalyzed [3+2] cycloaddition reactions of azides and alkynes (CuAAC) in water under strict click chemistry conditions. Copper(I) catalysts heterogenized onto commercially activated carbon materials (Cu-CC) and on another carbon material produced from vegetable biomass *using Argan nut shells* (Cu-CANS) were found to be versatile catalytic sources for sustainable CuAAC. These copper on carbon supports were prepared and fully characterized by using two types of activated carbons that exhibit different porosity and specific surface. The delineation of the nature of the catalytic copper species and the role of the carbon support in the CuAAC were addressed. These heterogeneous copper on carbon catalysts were recovered and reused until ten catalytic runs without any noticeable loss of activity.

## Introduction

1,2,3-Triazoles are important and useful non-classical bioisostere linkage heterocycles. They have several applications as agrochemical agents, dyes, corrosion inhibitors, photostabilizers, and photographic materials. Several 1,2,3-triazole derivatives show interesting biological activities under the so-called peptidomimetic substances (Chung et al., 2002; Mark, 2006; Run et al., 2007; Ostapenko et al., 2008; Zheng et al., 2008). The most popular method for the construction of 1,2,3-triazole moiety is the Huisgen reaction of [3+2] dipolar cycloaddition of azides with alkynes (Yan et al., 2005; Zhang et al., 2006). Copper(I)-catalyzed azide-alkyne cycloaddition (CuAAC) (Tornøe et al., 2002; Pachón et al., 2005; Bock et al., 2006) has emerged as one of the most reliable reactions under the click chemistry regime (Kolb and Sharpless, 2003; Yadav et al., 2007) that enables the practical and efficient preparation of 1,4-disubstituted-1,2,3-triazoles, from a wide range of substrates with excellent selectivity, which cannot be achieved by traditional Huisgen non-catalyzed thermal approaches (Huisgen, 1963).

The synthesis of 1,2,3-triazoles under CuAAC proceeds in the presence of copper salts as homogeneous catalysts, which make the separation and recovery of such copper catalysts very difficult (Gaetke and Chow, 2003; Hong et al., 2010; Yang et al., 2014). In addition, it is most likely that under homogeneous catalytic fashion, the final 1,2,3-triazoles can be contaminated by copper particles. So far, many challenges remain and much work still needs to be done in terms of the copper catalyst recovery and the obtention of copper-free 1,2,3-triazolic compounds. Therefore, an efficient and simple way to synthesize 1,2,3-triazoles is still necessary by working under strict click chemistry conditions and taking into account the sustainable chemistry criteria. In order to overcome these problems, recent works have focused on heterogeneous catalytic systems, which have several advantages such as good dispersion of the catalytically active sites, easier and safer handling, easy separation of the products from the reaction mixture and reusability of the catalyst. Thus, a good number of heterogeneous catalytic systems has been developed. For instance, immobilizing copper salts on silica and their use as heterogeneous catalysts in CuAAC (Miao and Wang, 2008; Coelho et al., 2010; Diz et al., 2015; Jumde et al., 2015), as magnetic nanoparticles (Xiong and Cai, 2013; Moghaddam and Ayati, 2015; Pourjavadi et al., 2015, 2016; Tajbakhsh et al., 2015; Bahrami and Arabi, 2016; Jahanshahi and Akhlaghinia, 2016), polymers (Bonami et al., 2009; Wallyn et al., 2011; Xiong et al., 2016), zeolites (Chassaing et al., 2007, 2008), hydroxyapatite (Masuyama et al., 2011), and carbon support such as charcoal (Lipshutz and Taft, 2006) have been subject of previous works.

**Scheme 1:**
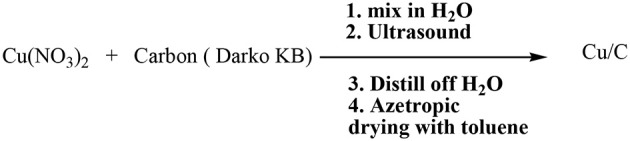
Lipshutz's protocol for the preparation of “copper-in-charcoal.”

The Lipshutz group has in fact described the preparation of copper-in-charcoal by impregnating of copper(II) nitrate in activated carbon (Darco-KB) using water as solvent under ultrasound radiation followed by distillation of water by azeotropic drying with toluene. The prepared copper-in-charcoal namely Cu/C was employed to assist the click of 1,2,3-triazoles using high temperature microwave conditions (Scheme 1).

Although the Lipshutz's copper-in-charcoal was efficient in assisting CuAAC, the studied triazole click reactions made use of a high catalyst loading of 10 mol%, an additional base (Et_3_N) and were peformed at high temperature (60 °C) in a hazardous solvent such as dioxane. The catalyst could be reused for only just three catalytic cycles without any loss of its activity (Lipshutz and Taft, 2006; Buckley et al., 2015).

**Scheme 2:**
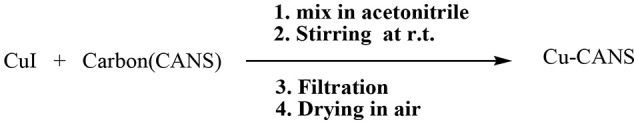
Preparation scheme of copper on carbon support made from *Argan nut shells* Cu-CANS.

In recent years, green synthesis and nature-friendly as well as sustainable resources and processes involving supported catalysts from agricultural wastes biomass have been found to be of increasing interest in the synthesis of heterocycles. In the context of our efforts to develop green, highly eco-efficient, and practical chemical methods utilizing bio-heterogeneous catalysts in [3+2] cycloaddition reactions of azides with alkynes (Bahsis et al., 2018), we herein report an easy sustainable protocol for the synthesis of 1,2,3-triazoles. For that, we use a new supported copper(I) on activated carbon materials easily made from agricultural wastes biomass such as *Argan Nut Shells*, namely Cu/CANS (Scheme 2). The choice of starting from CuX (X = I, Br, Cl) precursor and its impregnation into the pores of the carbon material in acetonitrile arises from the fact that such precursor of Cu(I), considered as the catalytically active specie in CuAAC, is nicely soluble in acetonitrile and the solutions are air stable and may be stored at room temperature.

Such a copper-supported on carbon Cu/CANS was found to be highly eco-efficient to assist CuAAC in water at room temperature as well as a recyclable heterogeneous catalyst. The commercially available activated carbon material (Cu/C) was also used for comparative purposes.

## Experimental Section

### General Methods

All chemicals were used as purchased without further purification. The reactions were performed under ambient conditions. NMR analyses were carried out on a spectrometer Bruker AC-400 MHz (400 MHz for proton, 100 MHz for carbon) by using deuterated chloroform as solvent. The chemical shifts (δ) are expressed in ppm. The high resolution mass spectra (HRMS) were recorded in the EI (70 eV) or FAB mode at the mass spectrometry service of the University of Valencia. Melting points were determined using a Stuart melting point apparatus SMP3, employing the capillary tubes. FT-IR spectra (4000–450 cm^−1^ range) were recorded with a Nicolet 5700 FT-IR spectrometer on samples prepared as KBr pellets. The polycrystalline sample of each support was lightly ground in an agate mortar, pestle and filled into 0.5 mm borosilicate capillary prior to being mounted and aligned on an Empyrean PANalytical powder diffractometer using Cu-Kα radiation (λ = 1.54056 Å). Three repeated measurements were collected at room temperature in the 10° < 2 Θ < 60° range with a step size of 0.01°. Scanning Electronic Microscopy (SEM) images were obtained with a HITACHI-S4100 equipment operated at 20 kV. The specific surface areas were determined from the dinitrogen adsorption/desorption isotherms (at 77 K) on a Quantachrome Autosorb-1 nautomatic analyzer using the BET (Brunauer-Emmett Teller) method. The pore size distribution was calculated from the N_2_ adsorption isotherms with the classic theory model of Barrett, Joyner and Halenda (BJH). The Scanning Electron Microscopy was carried out by using a VEGA3 TESCAN microscope and a high resolution JEOL Field Emission Gun-Scanning Electron Microscope (FEG-SEM).

### Procedure for the Synthesis of the Benzyl Azide Derivative

Benzyl bromide derivative (10.0 g, 58.5 mmol) and NaN_3_ (11.4 g, 175 mmol) were dissolved in 200 mL of dimethylformamide. The reaction mixture protected from light is stirred for 20 h at room temperature. After filtration, water was added to the filtrate and the product was extracted with dichloromethane three times. The organic phases were combined, dried over anhydrous MgSO_4_, then filtered-off and the solvents evaporated under reduced pressure, yielding a liquid product.

### Synthesis of 1-(Azidomethyl)Benzene

Colorless liquid. Yield: 90%. *R*_*f*_ = 0.8 in hexane/ethyl acetate (4:1 v/v). ^1^H NMR (400 MHz, CDCl_3_, δ ppm): 4.39 (s, 2H, CH_2_); 7.41–7.5 (m, 5H, CH_ar_). FT-IR (film on NaCl, cm^−1^): 2,096 cm^−1^ (N_3_).

### Synthesis of (Azidomethyl)-4-Methoxybenzene

Brown liquid. Yield: 87%. *R*_*f*_ = 0.64 in hexane/ethyl acetate (4:1 v/v). ^1^H NMR (400 MHz, CDCl_3_, δ ppm): 3.68 (s, 3H, OMe); 4.13 (s, 2H, CH_2_); 6.78–6.81 (d, *J* = 12.00 Hz, 2H, CH_ar_); 7.11–7.14 (d, *J* = 12.00 Hz, 2H, CH_ar_). ^13^C NMR (100 MHz, CDCl_3_, δ ppm): 54.3 (CH_2_); 55.2 (CH_3_); 114.2 (2CH_ar_); 127.4 (C_ar_); 129.7 (2CH_ar_); 159.64 (C_ar_).

### Procedure for the Synthesis of 1-Azidobenzene

Aniline (13 mmol) was suspended in 80 mL of hydrochloric acid (17%) at room temperature, then ethanol was added until a clear solution was obtained. The solution was cooled to 0°C and NaNO_2_ (19.5 mmol) was added in small portions. After stirring at 0°C for 15–30 min, NaN_3_ (19.5 mmol) was slowly added (*caution!! when handling NaN*_3_) and the mixture was stirred for additional 2 h at room temperature. The reaction mixture was extracted with diethyl ether (3 × 80 mL) and the combined organic fractions were washed with saturated NaHCO_3_ solution (3 × 50 mL) and with brine (50 mL). After drying over MgSO_4_ the ether was removed under reduced pressure and the desired azidobenzene was obtained without further purification as a brown liquid. Yield: 90%. *R*_*f*_ = 0.86 in hexane. ^1^H NMR (400 MHz, CDCl_3_, δ ppm): 7.08–7.44 (m, 5H, CH_ar_). ^13^C NMR (100 MHz, CDCl_3_, δ ppm): 119.46 (CH_ar_); 125.31 (2CH_ar_); 130.19 (2CH_ar_); 140.6 (C_ar_). FT-IR (film on NaCl, cm^−1^): 2,119 cm^−1^ (N_3_).

### Procedure for the Preparation of Activated Carbon-*Argan Nut Shells* (CANS)

The argan oil shells were collected, washed with distilled water, dried at room temperature and crushed by a plunger ball mill until a fine powder was obtained. The unmodified *Argan nut shells* material is abbreviated as ANS. The prepared raw material was treated by phosphoric acid with a mass proportion of H_3_PO_4_/ANS 1:1. The mixture was stirred using the plaster mill with a speed of 400 rpm/mn for 10 min. Subsequently, the mixture was kept at 120°C during 4 h. The carbonization of the obtained dried material was carried out in a muffle furnace at 500°C for 1 h at air atmosphere, whereas the activation with phosphate group would produce a well-developed porosity of the as-prepared activated carbon. After cooling, the recovered solid was crushed, washed several times with 0.15 M HCl solution, then with distilled water until the pH of the solution becomes neutral (pH ~ 7). The resulting material was dried completely and then crushed again by the plunger ball mill at a speed of 400 rpm for 15 min. Finally the obtained phosphate-containing carbon material was kept in a hermetic glass bottle.

### Synthetic Procedure of Copper on Carbon Catalyst

The commercially available carbon (CC) or carbon prepared from *Argan nut shells* (CANS) (1 g) was added to a solution of copper(I) iodide (250 mg) in acetonitrile (50 mL). The suspension was stirred overnight at room temperature and the resulting solid compounds were filtered-off, washed with acetonitrile (2 × 15 mL), diethyl ether (2 × 15 mL) and dried overnight. The catalyst was characterized by X-ray diffraction (XRD), scanning electronic microscopy (SEM), and infrared spectroscopy (FT-IR). Atomic Absorption Spectroscopy (AAS) (Aurora AI800) was used to determine the copper contents in both carbon materials.

### General Procedure of Copper on Carbon-Catalyzed Click of 1,2,3-Triazole From Azides and Alkynes

Azide (0.751 mmol, 1.2 equivalent), alkyne derivative (0.622 mmol, 1 equivalent), and CuI (0.005 equivalent) on carbon catalyst were placed in a reaction tube and 5 mL of water was added. The mixture was stirred for 6 h at room temperature. After completion of the reaction as evidenced by TLC, the 1,2,3-triazol product was extracted using diethyl ether and the Cu on carbon catalyst separated by filtration. The combined diethyl ether washings were evaporated under reduced pressure to afford the corresponding final pure 1,2,3-triazole. The recovered catalyst was dried and reused at least 10 times without any noticeable loss of its activity.

### Synthesis of 1-Benzyl-4-Phenyl-1*H*-1,2,3-Triazole (3a)

White solid. Yield: 94%. *R*_*f*_ = 0.3 in hexane/ethyl acetate (3:1 v/v). Mp = 130–132 °C. ^1^H NMR (400 MHz, CDCl_3_, δ ppm): 5.60 (s, 2H, CH_2_); 7.28–7.44 (m, 8H, CH_ar_); 7.68 (s, 1H, CH_triazole_); 7.81–7.83 (d, J = 8.00 Hz, 2H, CH_ar_). ^13^C NMR (100 MHz, CDCl_3_, δ ppm): 54.4 (CH_2_); 119.5 (CH_ar_); 125.9 (3CH_ar_); 127.8 (2CH_ar_); 128.2 (2CH_ar_); 129.2 (CH_triazole_); 131,5 (C_ar_); 135.0 (C_ar_); 148.3 (C_triazole_). HRMS (FAB^+^) *m/z*: Calcd for C_15_H_14_N_3_: 236.1188; Found: 236.1177.

### Synthesis of Methyl 4-(1-Benzyl-1*H*-1,2,3-Triazol-4-yl)Benzoate (3b)

White solid. Yield: 88%. *R*_*f*_ = 0.42 in hexane/ethyl acetate (2:1 v/v). Mp = 176–178 °C. ^1^H NMR (400 MHz, CDCl_3_, δ ppm): 3.91 (s, 3H, OCH_3_); 5.58 (s, 2H, CH_2_); 7.33–7.38 (m, 5H, CH_ar_); 7.74 (s, 1H, CH_triazole_); 7.86–7.89 (d, *J* = 12.00 Hz, 2H, CH_ar_); 8.05–8.08 (d, *J* = 12.00 Hz, 2H, CH_ar_). ^13^C NMR (100 MHz, CDCl_3_, δ ppm): 52.3 (CH_3_); 54.5 (CH_2_); 120.4 (CH_ar_); 125.6 (2CH_Ar_); 128.3 (2CH_ar_); 129.1 (2CH_ar_); 129.4 (2C_ar_); 129.7 (CH_triazolic_); 130.3 (C_ar_); 134.5 (C_triazolic_); 134.9 (C_ar_); 147.3 (C_ar_); 166.9 (CO). HRMS (FAB^+^) *m/z*: Calcd for C_17_H_16_N_3_O_2_: 294.1243; Found: 294.1245.

### Synthesis of 4-(1-Benzyl-1*H*-1,2,3-Triazol-4-yl)-N,N-Dimethylbenzenamine (3c)

Yellow solid. Yield: 89%. *R*_*f*_ = 0.26 in hexane/ethyl acetate (2:1 v/v). Mp = 202–204 °C. ^1^H NMR (400 MHz, CDCl_3_, δ ppm): 2.97 (s, 3H, CH_3_); 5.54 (s, 2H, CH_2_); 6.72–6.75 (d, *J* = 12.0 Hz, 2H, CH_ar_); 7.26–7.38 (m, 5H, CH_ar_); 7.53 (s, 1H, CH_triazole_); 7.65–7.68 (d, *J* = 12.00 Hz, 2H, CH_ar_). ^13^C NMR (100 MHz, CDCl_3_, δ ppm): 40.6 (CH_2_); 54.2 (CH_3_); 112.5 (2CH_ar_); 118.1 (C_ar_); 126.8 (CH_ar_); 128.1 (2CH_ar_); 128.8 (2CH_ar_); 129.2 (2CH_ar_); 135.1 (C_ar_+ CH_triazolic_); 150.5 (C_triazolic_). HRMS (FAB^+^) *m/z*: Calcd for C_17_H_19_N_4_: 279.1609; Found: 279.1599.

### Synthesis of 4-(1-Benzyl-1*H*-1,2,3-Triazol-4-yl)Benzenamine (3d)

White solid. Yield: 92%. *R*_*f*_ = 0.48 in hexane/ethyl acetate (1:2 v/v). M_P_ = 184°C. ^1^H NMR (400 MHz, CDCl_3_, δ ppm): 4.85 (s, 2H, NH_2_); 5.56 (s, 2H, CH_2_); 6.72–6.75 (d, *J* = 12.00 Hz, 2H, CH_ar_); 7.31–7.35 (m, 5H, CH_ar_); 7.49–7.52 (d, *J* = 12.00 Hz, 2H, CH_ar_); 8.05 (s, 1H, CH_triazole_). ^13^C NMR (100 MHz, CDCl_3_, δ ppm): 55.36 (CH_2_); 116.83 (2CH_ar_); 121.07 (C_ar_); 121.44 (CH_ar_); 128.17 (2CH_ar_); 129.43 (2CH_ar_); 129.96 (2CH_ar_); 130.44 (CH_triazolic_); 137.32 (C_ar_); 149.90 (C_triazolic_); 150.36 (C_ar_). HRMS (FAB^+^) *m/z*: Calcd for C_15_H_15_N_4_: 251.1297; Found: 251.1299.

### Synthesis of Ethyl 1-Benzyl-1*H*-1,2,3-Triazole-4-Carboxylate (3e)

White solid. Yield: 95%. *R*_*f*_ = 0.29 in hexane/ethyl acetate (2:1 v/v). M_p_ = 83–85°C. ^1^H NMR (400 MHz, CDCl_3_, δ ppm): 1.35–1.40 (t, *J* = 10.00 Hz, 3H, CH_3_); 4.35–4.42 (q, *J* = 9.33 Hz, 4H, OCH_2_); 5.57 (s, 2H, CH_2_); 7.26–7.29 (m, 3H, CH_ar_); 7.37–7.4 (m, 3H, CH_ar_); 7.96 (s, 1H, CH_triazole_). ^13^C NMR (100 MHz, CDCl_3_, δ ppm): 14.4 (CH_3_); 54.6 (CH_2_); 61.4 (CH_2_); 127.4 (CH_ar_); 128.4 (2CH_ar_); 129.3 (CH_ar_); 129.4 (CH_ar_); 133.8 (CH_triazolic_); 140.7 (C_triazolic_); 160.8 (CO). HRMS (FAB^+^) *m/z*: Calcd for C_12_H_14_N_3_O_2_: 232.1086; Found: 232.1087.

### Synthesis of 1,4-Diphenyl-1*H*-1,2,3-Triazole (3f)

White solid. Yield: 89%. *R*_*f*_ = 0.48 in hexane/ethyl acetate (3:1 v/v). M_P_ = 183–184°C. ^1^H NMR (400 MHz, CDCl_3_, δ ppm): 7.33–7.4 (m, 2H, CH_ar_); 7.44–7.49 (t, *J* = 7.47 Hz, 2H, CH_ar_); 7.53–7.58 (t, *J* = 7.56 Hz, 2H, CH_ar_); 7.79–7.81 (d, *J* = 7.80 Hz, 2H, CH_ar_); 7.92–7.94 (d, *J* = 7.93 Hz, 2H, CH_ar_); 8.22 (s, 1H, CH_triazole_). ^13^C NMR (100 MHz, CDCl_3_, δ ppm): 118.07 (2CH_ar_); 120.98 (C_ar_); 126.34 (2 CH_ar_); 128,98 (CH_ar_); 129,31 (CH_ar_); 129,37 (2CH_ar_); 130,23 (2CH_ar_); 130.34 (CH_triazolic_); 130.73 (C_ar_); 137.47 (C_triazolic_). HRMS (FAB^+^) *m/z*: Calcd for C_14_H_12_N_3_: 222.1031; Found: 222.1029.

### Synthesis of N,N-Dimethyl-4-(1-Phenyl-1*H*-1,2,3-Triazol-4-yl)Benzenamine (3g)

Yellow solid. Yield: 87%. *R*_*f*_ = 0.5 in hexane/ethyl acetate (2:1 v/v). M_p_ = 169–171 °C. ^1^H NMR (400 MHz, CDCl_3_, δ ppm): 2.19 (s, 1H, CH_3_); 6.81–6.84 (d, *J* = 12.00 Hz, 2H, CH_ar_); 7.43–7.58 (m, 5H, CH_ar_); 7.80–7.82 (d, *J* = 8.00 Hz, 2H, CH_ar_); 8.08 (s, 1H, CH_triazole_). ^13^C NMR (100 MHz, CDCl_3_, δ ppm): 40.6 (2CH_3_); 112.6 (2CH_ar_); 116.1 (2CH_ar_); 120.6 (5CH_ar_); 126.9 (C_ar_+CH_triazolic_); 130.1 (C_triazolic_); 141 (C_ar_). HRMS (FAB^+^) *m/z*: Calcd for C_16_H_17_N_4_: 265.1453; Found: 265.1444.

### Synthesis of Ethyl 1-Phenyl-1*H*-1,2,3-Triazole-4-Carboxylate (3h)

White solid. Yield: 73%. *R*_*f*_ = 0.56 in hexane/ethyl acetate (2:1 v/v). M_p_ = 75–77°C. ^1^H NMR (400 MHz, CDCl_3_, δ ppm): 1.41–1.45 (t, *J* = 8.00 Hz, 3H, CH_3_); 4.43–4.5 (q, *J* = 9.33 Hz, 4H, CH_2_); 7.46–7.58 (m, 3H, CH_ar_); 7.74–7.76 (m, 3H, CH_ar_); 8.51 (s, 1H, CH_triazole_). ^13^C NMR (100 MHz, CDCl_3_, δ ppm): 31.1 (CH_3_); 61.6 (CH_2_); 120.9 (C_ar_); 125.6 (2CH_ar_); 129.6 (2CH_ar_); 130.1 (CH_triazolic_); 136.5 (C_triazolic_); 141 (CO). HRMS (FAB^+^) *m/z*: Calcd for C_11_H_12_N_3_O_2_: 218.0929; Found: 218.0924.

### Synthesis of 1-(4-Methoxybenzyl)-4-Phenyl-1*H*-1,2,3-Triazole (3i)

White solid. Yield: 96%. *R*_*f*_ = 0.28 in hexane/ethyl acetate (3:1 v/v). M_p_ = 132–135°C. ^1^H NMR (400 MHz, CDCl_3_, δ ppm): 3.73 (s, 3H, OCH_3_); 5.43 (s, 2H, CH_2_); 6.82–6.85 (d, *J* = 12.00 Hz, 2H, CH_ar_); 7.18–7.82 (m, 5H, CH_ar_); 7.54 (s, 1H, CH_triazole_); 7.69–7.72 (d, *J* = 12.00 Hz, 2H, CH_ar_). ^13^C NMR (100 MHz, CDCl_3_, δ ppm): 53.9 (CH_2_); 55.4 (CH_3_); 114.6 (2CH_ar_); 119.3 (2CH_ar_); 125.8 (2CH_ar_); 128.2 (CH_ar_); 128.9 (2CH_ar_); 129.8 (C_ar_); 128.8 (2CH_ar_); 130.7 (C_ar_+CH_triazolic_); 148.2 (C_triazolic_); 160.1 (CH_ar_). HRMS (FAB^+^) *m/z*: Calcd for C_16_H_16_N_3_O: 266.1293; Found: 266.1286.

### Synthesis of Methyl 4-(1-(4-Methoxybenzyl)-1*H*-1,2,3-Triazol-4-yl)Benzoate (3j)

White solid. Yield: 80%. *R*_*f*_ = 0.66 in hexane/ethyl acetate (1:1 v/v). M_p_ = 183–185°C. ^1^H NMR (400 MHz, CDCl_3_, δ ppm): 3.74 (s, 3H, OCH_3_); 3.84 (s, 3H, OCH_3_); 5.44 (s, 2H, CH_2_); 6.83–6.86 (d, *J* = 12.00 Hz, 2H, CH_ar_); 7.19–7.22 (d, *J* = 12.00 Hz, 2H, CH_ar_); 7.63 (s, 1H, CH_triazole_); 7.77–7.80 (d, *J* = 12.00 Hz, 2H, CH_ar_); 7.97–8.00 (d, *J* = 12.00 Hz, 2H, CH_ar_). ^13^C NMR (100 MHz, CDCl_3_, δ ppm): 52.1 (CH_3_); 53.9 (CH_2_); 55.3 (CH_3_); 114.5 (2CH_ar_); 125.4 (2CH_ar_); 129.5 (2CH_ar_); 129.7 (2C_ar_); 130.1 (CH_triazolic_); 134.9 (C_triazolic_); 147.1 (C_ar_); 160.0 (C_ar_); 166.7 (CO). HRMS (FAB^+^) *m/z*: Calcd for C_18_H_18_N_3_O_3_: 324.1348; Found: 324.1338.

### Synthesis of 4-(1-(4-Methoxybenzyl)-1*H*-1,2,3-Triazol-4-yl)Benzenamine (3k)

Yellow solid. Yield: 78%. *R*_*f*_ = 0.34 in hexane/ethyl acetate (1:1 v/v). M_p_ = 121–123°C. ^1^H NMR (400 MHz, CDCl_3_, δ ppm): 2.09 (s, 1H, NH_2_); 3.73 (s, 3H, OCH_3_); 5.40 (s, 2H, CH_2_); 6.61–6.64 (d, *J* = 12.00 Hz, 2H, CH_ar_); 6.81–6.84 (d, *J* = 12.00 Hz, 2H, CH_ar_); 7.17–7.19 (d, *J* = 8.00 Hz, 2H, CH_ar_); 7.41 (s, 1H, CH_triazole_); 7.49–7.52 (d, *J* = 12.00 Hz, 2H, CH_ar_). ^13^C NMR (100 MHz, CDCl_3_, δ ppm): 53.6 (CH_3_); 55.3 (CH_2_); 114.4 (2CH_ar_); 115.1 (2CH_ar_); 117.9 (2CH_ar_); 126.8 (2CH_ar_); 126.9 (2C_ar_); 129.6 (C_ar_ + CH_triazolic_); 146.4 (C_triazolic_); 159.8 (C_ar_). HRMS (FAB^+^) *m/z*: Calcd for C_16_H_17_N_4_O: 381.1402; Found: 281.1398.

## Results and Discussion

### Synthesis and Characterization of Copper on Carbon Catalyst

The synthesis of activated carbon from the local biomass such as *Argan nut shells* by the chemical activation method was adopted in this study. The advantage of this activation is to opt for low pyrolysis temperatures and a smaller activation cost. The high quality of activated carbon with a very large porous texture and high surface area was prepared from *Argan nut shells* biomass by using orthophosphoric acid as activating agent. This activation process aims to develop and modulate the porous structure of carbon, leading to a very sharp increase in its specific surface area. Indeed, the specific surface area of activated carbon is one of the most important physical structure parameters, the accuracy of its measured value being essential for realistic reference significance. In this work, the dinitrogen adsorption method was adopted to measure the specific surface area of activated carbon and the results obtained are summarized in Table 1. In this work, the Brunauer–Emmett–Teller (BET) method was adopted to measure the specific surface area of activated carbon and the corresponding results are also summarized in Table 1. It can be deduced from such results that commercially available carbon material CC, used for comparative purposes, presents a specific surface area equal to S_BET_ = 702.76 m^2^/g with microspores rounding a size < 2 nm, while the prepared carbon material CANS exhibits a highly porous structure as shown by the high found value of the specific surface area found to be S_BET_ = 1151.75 m^2^/g) and the mesoporous character evaluated at 2.2 nm. This behavior allows easy access and contact between the reagents employed in a given heterogeneous catalytic protocol.

**Table 1:** The specific surface area, average pore diameter, and V_total_ of pores of CANS and CC.

**Activated carbon**	**S_**BET**_ (m^**2**^/g)**	**Average pore diameter (nm)**	**V_**Total**_ of pores (cm^**3**^/g)**
CANS	1151.75	2.204	0.635
CC	702.76	1.168	0.193

*CANS, carbon from Argan Nut Shells biomass; CC, commercially carbon*.

The Cu-carbon catalysts were prepared by impregnation of carbon supports with CuI in acetonitrile overnight at room temperature. The reaction mixture filtered, and the solid successively washed with acetonitrile, diethyl ether and dried under vacuum. The catalysts were characterized by several techniques such as Scanning electron microscopy (SEM), Energy-dispersive X-ray (EDX), X-ray diffraction (XRD) analysis, and FT-IR spectroscopy.

**Figure 1:**
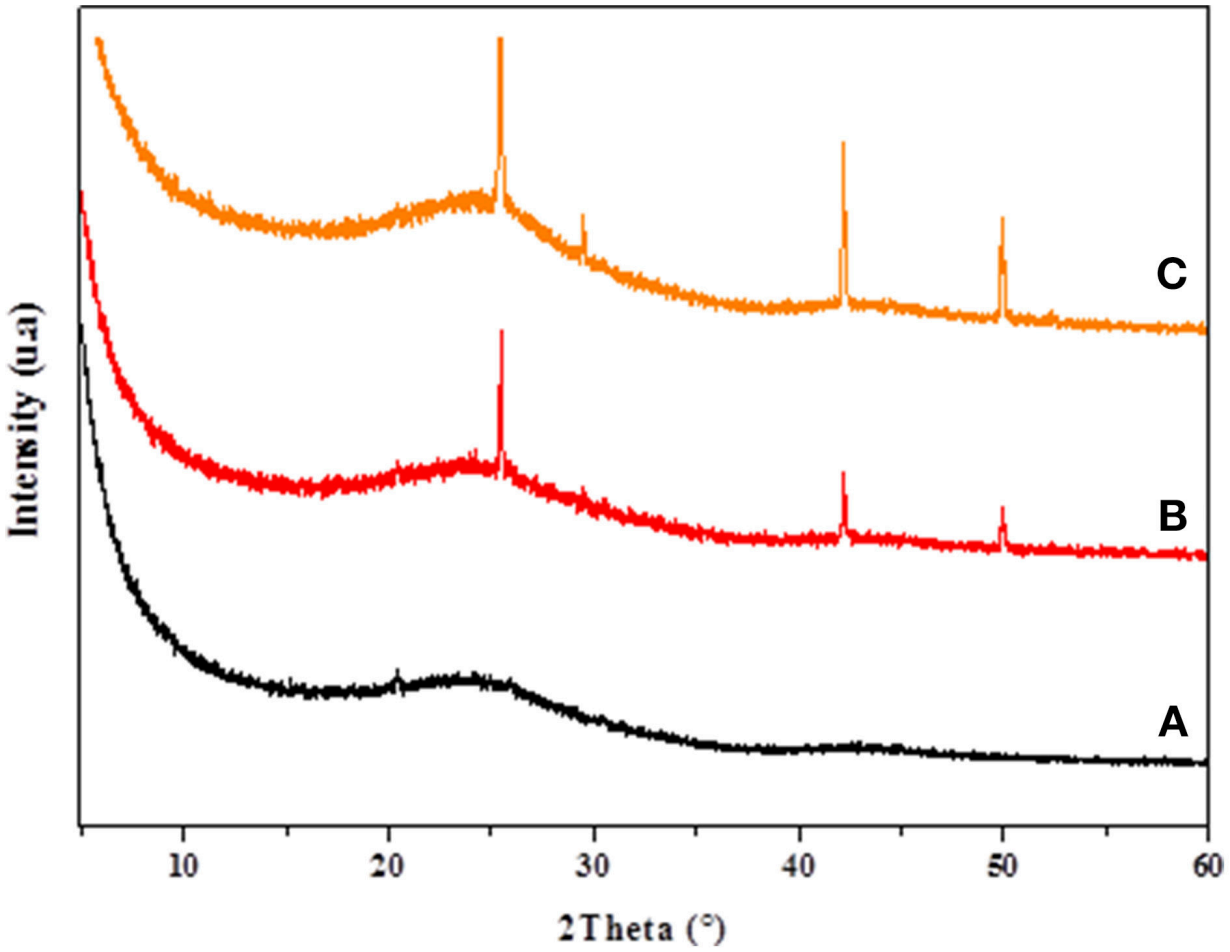
XRD results of CANS support **(A)**, Cu-CANS material **(B)**, and phenylacetylene in Cu-CANS **(C)**.

**Figure 2:**
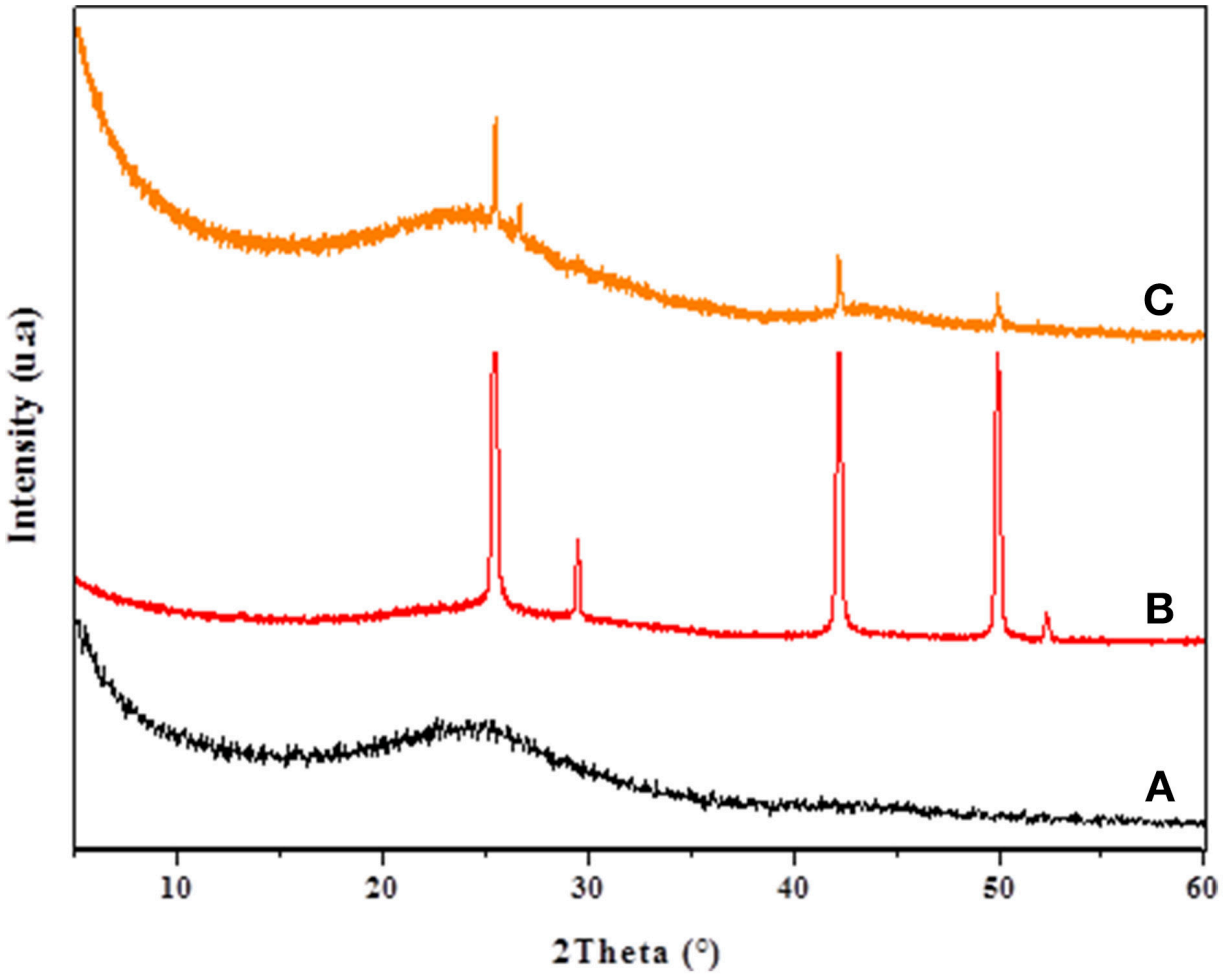
XRD results of CC support **(A)**, Cu-CC material **(B)**, and phenylacetylene in Cu-CC **(C)**.

The XRD patterns of CC, CANS, Cu-CC, and Cu-CANS are presented in Figures 1, 2. The obtained XRD patterns displayed the following diffraction peaks (2θ [°]): 25.5, 29.46, 42.19, and 49.91° for Cu-CANS, (Figure 1) which can be correlated to the (111), (200), (220), and (311) *hkl* indices, respectively, of CuI (JCPDS no. 06-0246). As shown in Figure 2, five peaks at 2θ = 25.47, 29.46, 42.34, 49.96, and 52.30° corresponding to the (111), (200), (220), (311), and (222) planes of CuI (JCPDS no. 06-0246) were observed in the pattern of the Cu-CC composite, indicating that CuI has been successfully loaded on the carbons. The average crystallite size *D* of the nanoparticles is calculated from the Scherrer equation: *D* = *K*λ/(βcosθ), where *K* is the Debye-Scherrer constant (0.9), λ is the X-ray wavelength, β FWHM (full-width at half-maximum or half width) is in radians, and θ is the Bragg diffraction angle. Here, the (111) peak of the highest intensity was picked out to evaluate the particle diameter of CuI. The values of the *D* constants were calculated to be about 18.81 and 26.15 nm for nm for Cu-CC and Cu-CANS, respectively.

**Figure 3:**
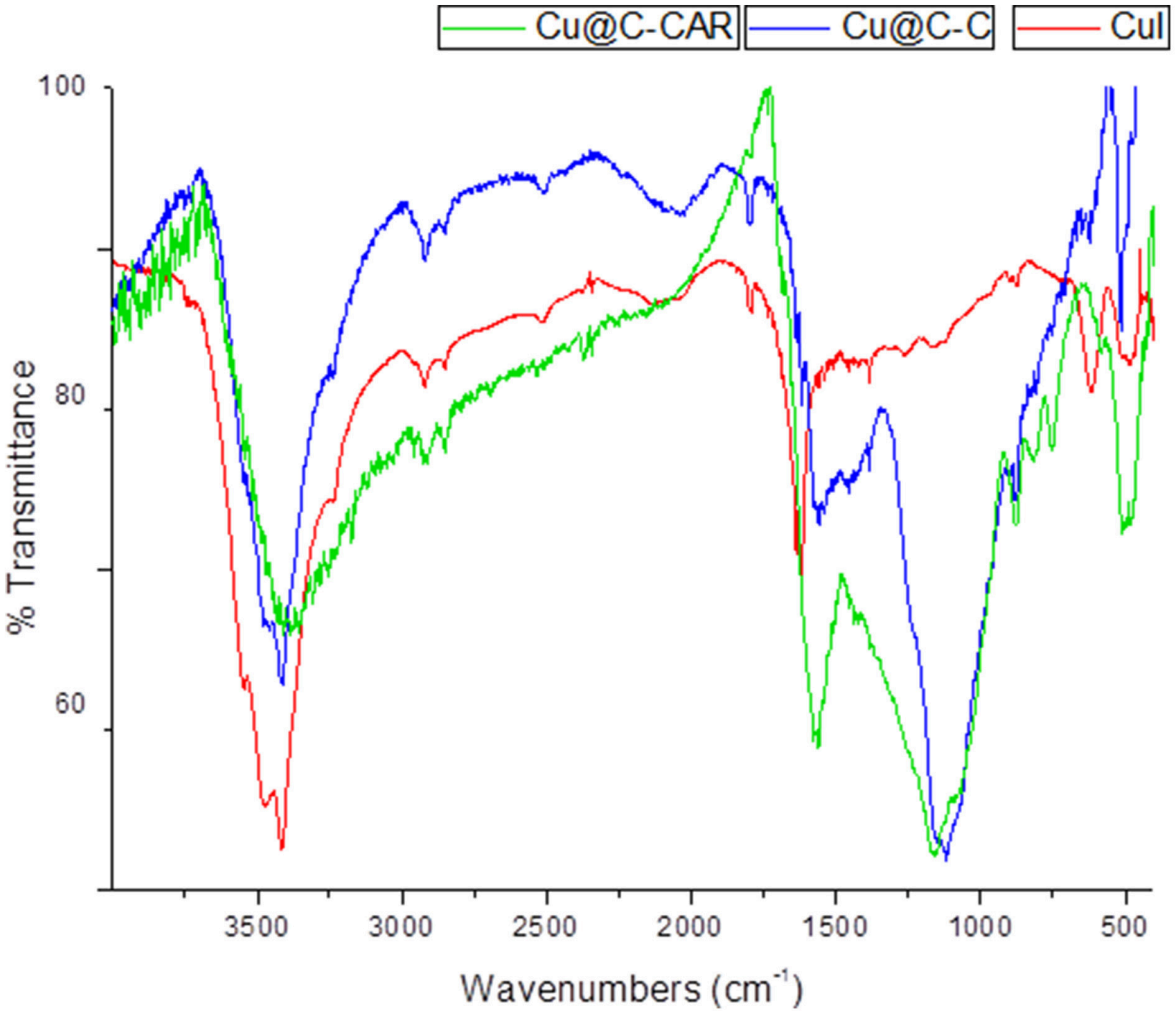
Comparative FT-IR spectra of CuI precursor (red), Cu-CC (blue), and Cu-CANS material (green).

Infrared spectroscopy is one of the most widely used techniques in heterogeneous catalysis to characterize and identify the purity of solids by the presence of characteristic bands of extraneous compounds. Comparison between FT-IR spectrum of copper particles on activated carbon with that of copper iodide and with the carbon support reveals the appearance of weak absorption bands at 496 cm^−1^ (Figure 3). This is related to the supported copper particles on activated carbon. Furthermore, the change observed in the absorption peaks of the carbonyl group from 1654.7 to 1650.4 and 1561.4 to 1557 cm^−1^ is related to the coordination of oxygen atoms (acetate and phosphate) to copper metal ions (Samim et al., 2007; Ghouma et al., 2017). The presence of oxygen atoms (phosphate-containing groups) may afford the assembly of polynuclear copper ions in the carbon support because of the known bridging ability of the phosphate groups (Ikotun et al., 2010).

**Figure 4:**
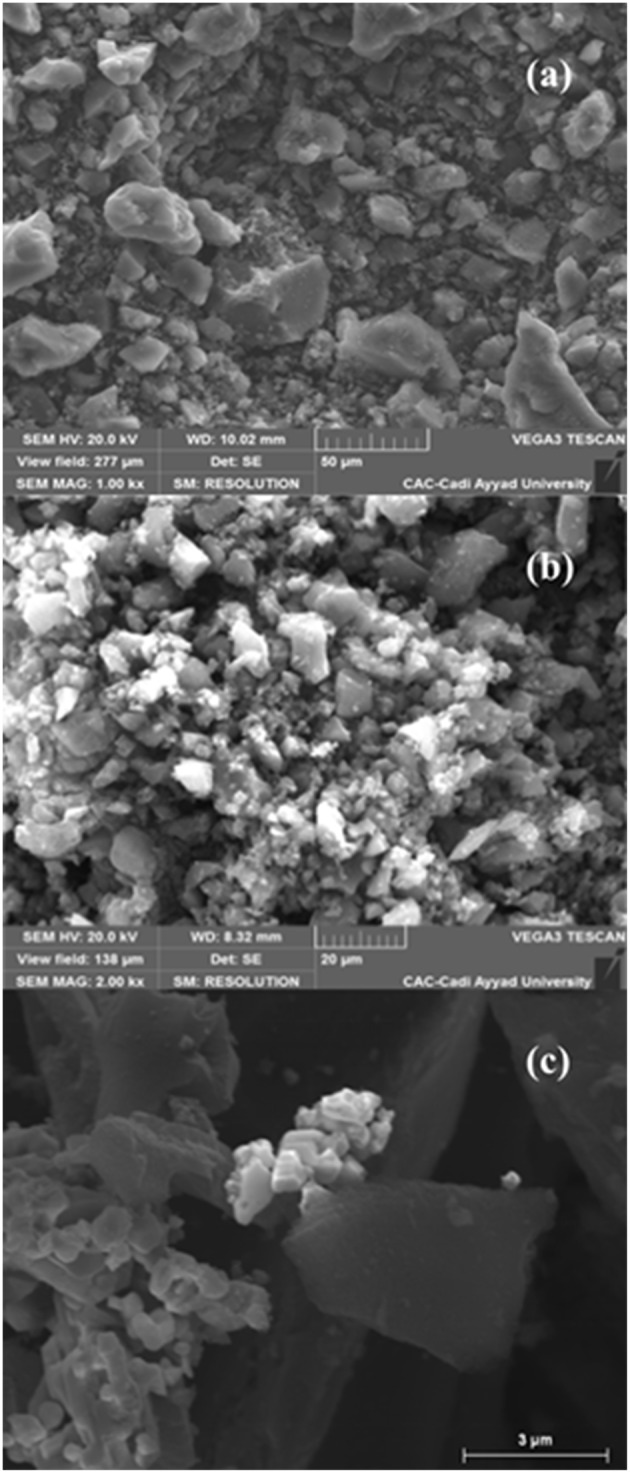
SEM images of CANS support **(a)**, Cu-CANS **(b)**, and Cu-CC material **(c)**.

**Figure 5:**
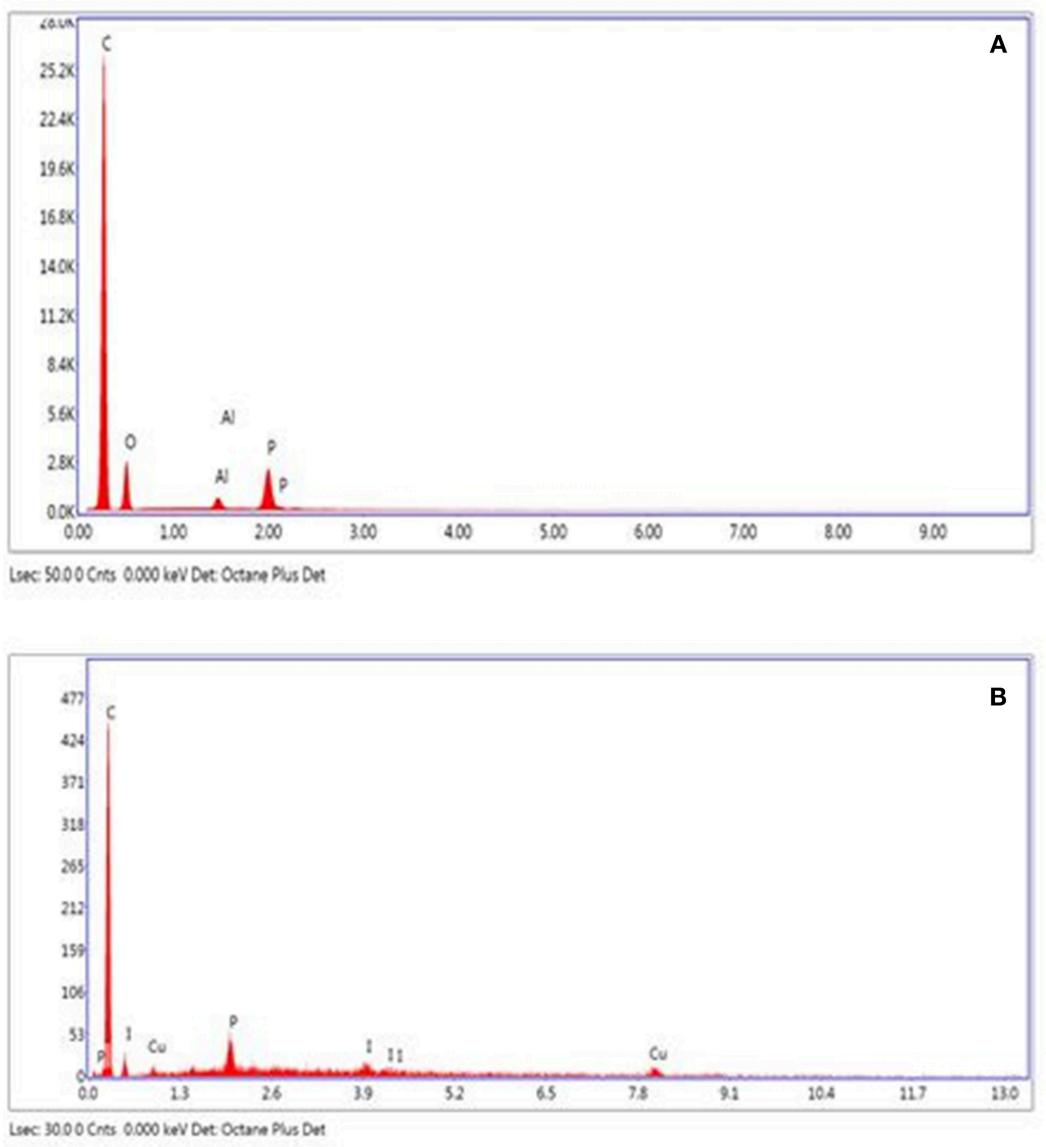
EDX spectrum of **(A)** CANS and **(B)** Cu-CANS catalyst.

We used the scanning electron microscopy (SEM) to study and visualize the morphology of the surface before and after the immobilization of copper on activated carbons. The SEM images of activated carbons and catalysts clearly reveal the presence of different size pores on the raw activated carbons and showed that both catalysts have the same shape and contain a mosaic of copper particles of different sizes and morphologies (Figure 4). The energy dispersive X-ray (EDX) results obtained from the SEM analysis for the CANS, and Cu-CANS showed the presence of C, O, Al and P atoms for the former and C, Cu, and P atoms for the latter (Figure 5).

The copper contents in the carbon materials were determined by AAS analysis and found to be 1.38 wt% of Cu for Cu-CANS and 3.82 wt% of Cu for Cu-CC. Thus, 100 mg of carbon composite contains 2.18 mol% of copper for Cu-CANS and 6.01 mol% of copper for Cu-CC. The low copper loadings in Cu-CANS compared with Cu-CC can be explained by their respective preparation method and the functionalizing groups that may coordinate to the copper ions in the carbon material.

### Adsorption Isotherms

The equilibrium adsorption isotherms are very important for understanding the mechanism of the CuI adsorption on both activated carbons investigated. The adsorption data were analyzed with the help of the following linear forms of Freundlich and Langmuir isotherms.

The Langmuir isotherm is valid for monolayer adsorption on surface containing a finite number of identical sites (Langmuir, 1916). The linear form of the Langmuir isotherm can be represented by the following equation:

$$\begin{array}{l} \frac{C_{e}}{q_{e}}=\frac{1}{{\bar{q}}_{m}K_{L}}+\frac{1}{q_{m}}C_{e} \end{array}$$

where *C*_*e*_ (mg/L) represents the equilibrium concentration of the adsorbate, *q*_*e*_ is the amount adsorbed at equilibrium (mg/g), and *K*_*L*_ (L/mg) and *q*_*m*_ (mg/g) stand for the Langmuir constant and the maximum amount of adsorbate, respectively.

The Freundlich isotherm model is an empirical equation based on sorption on a heterogeneous surface or surface supporting sites of varied affinities (Freundlich, 1906). The linearized Freundlich model is represented by the following equation:

$$\begin{array}{l} \log\left(q_{e}\right)=\log\left(K_{f}\right)+\frac{1}{n}\log\left(C_{e}\right) \end{array}$$

where *K*_*f*_ (mg/g) is the Freundlich constants related to the sorption capacity and *n* is the heterogeneity factor.

As shown in Table 2, the comparison of the Freundlich and Langmuir models, reveals that the values of the correlation coefficient of the Freundlish isotherm (*R*^2^ = 0.98 and 0.95) are high for both activated carbons. This result suggests that the CuI was heterogeneously adsorbed on a multilayer surface and the values found for *n* were >1, a feature which proves that the adsorption on both adsorbents is favorable.

**Table 2:** Isotherm constants for adsorption of CuI on CANS and CC activated carbons.

**Isotherm model**	**Langmuir**			**Freundlich**		
	***q_***max***_***	***K_***L***_***	***R*^**2**^**	***K_***F***_***	***n***	***R*^**2**^**
CANS	1,250	0.003	0.86	7.58	1.29	0.98
CC	0.63	0.037	0.90	1.05	1.09	0.95

### Catalytic Activity of the Cu-Carbon Catalytic System in the Synthesis of 1,2,3–Triazole Derivatives

In order to explore the catalytic activity of the synthesized catalysts and to optimize the reaction conditions for the [3+2] cycloaddition, the reaction between benzyl azide and phenylacetylene was chosen as a model [3+2] cycloaddition reaction. As starting point, different reaction conditions such as copper sources, solvent, and amount of catalyst were investigated (see Table 3). The control experiments show that the [3+2] cycloaddition reaction azide-alkyne does not take place in the absence of the catalyst (entries 1–3, Table 3). Moreover, the effect of copper(I) sources supported within the pores of both CC and CANS was examined (entries 4–6, Table 3). In fact, the results shown in Table 3 confirm the recently established catalytic activity trend found in CuAAC assisted by several N-heterocylic carbene copper(I) complexes, namely [(NHC)CuX] [NHC = *N*-Heterocycle carbene; X = I, Cl, Br]: [CuI(NHC)] > [CuBr(NHC)] > [CuCl(NHC)] (Díez-Gonzalez et al., 2010). Our results show that among the studied Cu(I)-carbon catalysts, CuI-CC and CuI-CANS are the most efficient ones for the 1,2,3-triazole click reactions performed under strict click chemistry conditions, specifically by using water as solvent and working at room (entries 4–6, Table 3). Having identified the CuI-CC and CuI-CANS as efficient catalytic systems for the reactions; we then explored the effect of the catalyst amount on the conversion yields and the catalyst loading being enhanced from 0.1 to 3 mol%. As a result, the conversion yields were increased from 70 to 99 % by the increasing of catalyst amount (entries 6–8, Table 3). However, no elevated conversion yields were observed once the catalyst amount becomes greater than 3 mol%. Consequently, in both Cu(I)-CC and Cu(I)-CANS catalysts, a catalyst loading of 0.5 mol% appears to be optimal with respect to excellent yields and short reaction times. Furthermore, the catalytic reaction was performed in different organic solvents, as shown in Table 3. We found that the catalytic conversion yields in water and acetonitrile were higher than those in other organic solvents, such as ethanol, methanol, toluene and hexane (entries 8–12, Table 3). As a matter of consequence, water known as a benign and inexpensive solvent, was then used as the solvent of choice.

**Table 3:** Catalyst and conditions screening for the cycloaddition of benzyl azide and phenylacetylene^a^.

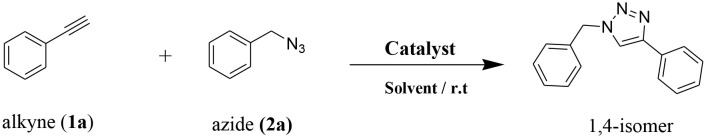					
**Entry**	**Catalyst**	**Cat. loading (mol %)**	**Solvent**	**Time (h)**	**Yield (%)^b^**
1	–	–	Water	24	0
2	CC	–	Water	24	0
3	CuCl-CC	5	Water	6	66
4	CuBr-CC	5	Water	6	74
5	CuI-CC	3	Water	6	99
6	CuI-CC	2	Water	6	98
7	CuI-CC	0.5	Water	6	77
8	CuI-CC	0.1	Water	6	74
9	CuI-CC	5	Ethanol	6	85
10	CuI-CC	5	Methanol	6	74
11	CuI-CC	5	Toluene	6	80
12	CuI-CC	5	Acetonitrile	6	98
13	CuI-CC	5	Hexane	6	35

a *Reaction conditions: benzylazide (0.75 mmol); phenylacetylene (0.62 mmol); solvent (5 mL); and catalyst were mixed and stirred at room temperature*.

b *Isolated yields*.

After optimizing the reaction conditions, in order to explore the scope and generality of this protocol, several alkynes such as para-substituted aryl alkyne derivatives and activated alkyne with azides such as para-substituted benzyl and phenyl azides were used as substrates for the synthesis of 1,4-disubstituted-1,2,3-triazoles. The results are given in Table 4. They show that the reactions are equally facile with both electron-donating and electron-withdrawing substituents present on the aryl alkynes and benzyl azides, as most of the reactions were completed within 6 h, resulting one regioisomer in good to high yields of the corresponding 1,4-disubstituted-1,2,3-triazole (see Table 4). These 1,2,3-triazoles were obtained in good turnover numbers ranging from 122 to 196. All the synthesized triazole derivatives were characterized by NMR spectroscopy and HRMS analysis (see Supplementary Material).

**Table 4:** Cycloaddition of azides and alkynes catalyzed by copper-carbon catalysts^a^.

**Entry**	**Alkyne**	**Azide**	**Product**	**Catalyst**	**Yield^b^ (%)**	**TON^c^**	**TOF^d^**
1	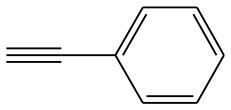	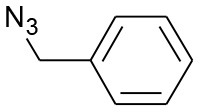	**3a**	Cu-CC	77	154	25.66
				Cu-CANS	95	190	31.66
2	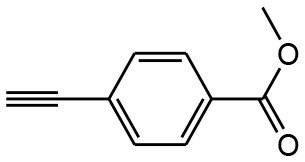	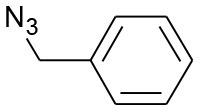	**3b**	Cu-CC	60	132	22.00
				Cu-CANS	88	176	29.33
3	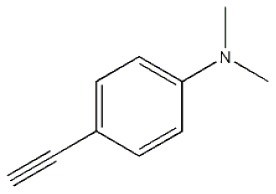	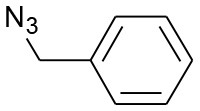	**3c**	Cu-CC	91	182	30.33
				Cu-CANS	89	178	29.66
4	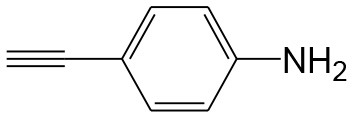	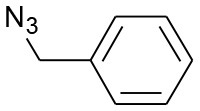	**3d**	Cu-CC	71	142	23.00
				Cu-CANS	92	184	30.66
5	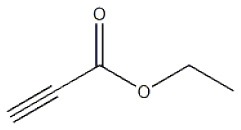	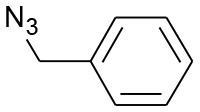	**3e**	Cu-CC	76	152	25.33
				Cu-CANS	95	190	31.66
6	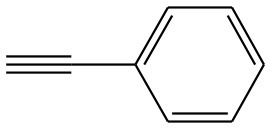	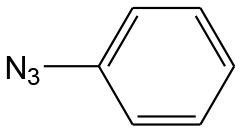	**3f**	Cu-CC	75	150	25.00
				Cu-CANS	94	188	31.33
7	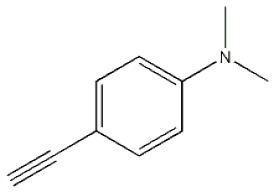	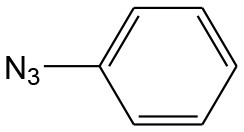	**3g**	Cu-CC	76	152	25.33
				Cu-CANS	87	122	20.33
8	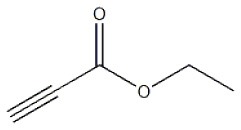	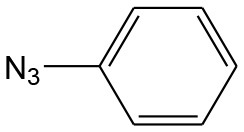	**3h**	Cu-CC	61	182	30.33
				Cu-CANS	73	146	24.33
9	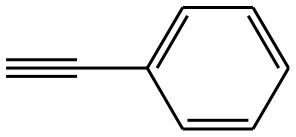	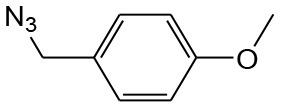	**3i**	Cu-CC	76	152	25.33
				Cu-CANS	96	192	32.00
10	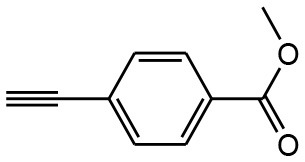	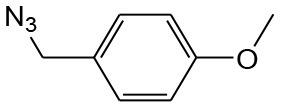	**3j**	Cu-CC	74	148	24.66
				Cu-CANS	80	160	26.66
11	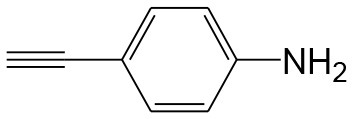	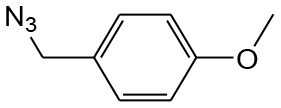	**3k**	Cu-CC	76	152	25.33
				Cu-CANS	78	156	26.00

a *Reaction conditions: azide (0.75 mmol); alkyne (0.62 mmol); water (5 mL); catalyst (0.005 equivalent) mixed at room temperature*.

b *Isolated yields*.

c *TON, Turnovers number (moles substrate/moles of catalyst)*.

d *TOF, Turnover frequency (TON/time of reaction)*.

**Figure 6:**
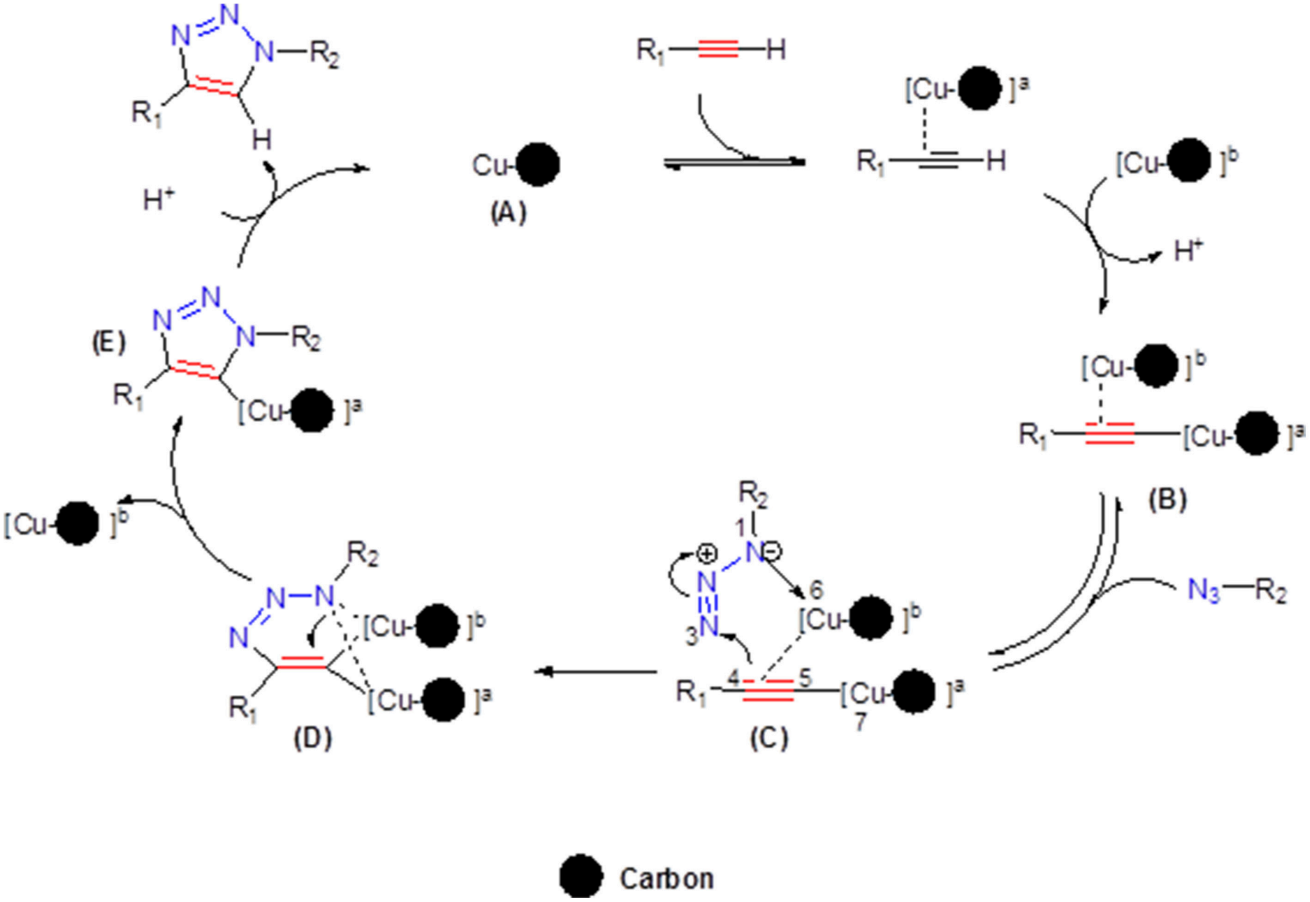
The proposed mechanism for the formation of 1-4-disubstituted-1,2,3-triazoles catalyzed by Cu/carbon.

On the basis of our previous reports (Ben El Ayouchia et al., 2018), a stepwise mechanism of CuAAC is outlined in Figure 6. The electron density of the alkyne in the proposed mechanism is reduced by the copper(I) ion stabilized in pore of the carbon support (**A**) forming the dinuclear copper-acetylide (**B**), enabling a facile nucleophilic attack by the organoazide, and then resulting in the corresponding complex (**C**). The next step consists of a nucleophilic attack at N3 of the organoazide by the acetylide carbon C4 forming the first covalent C—N bond and then producing the intermediate (**D**). The ring contraction of D leads to the formation of the triazolyl-copper (**E**). The last step corresponds to a fast protonation of the copper triazolide, releasing the final 1,2,3-triazole product as 1,4-regioisomer.

### Stability and Recycling of the Copper-Carbon Catalyst

**Figure 7:**
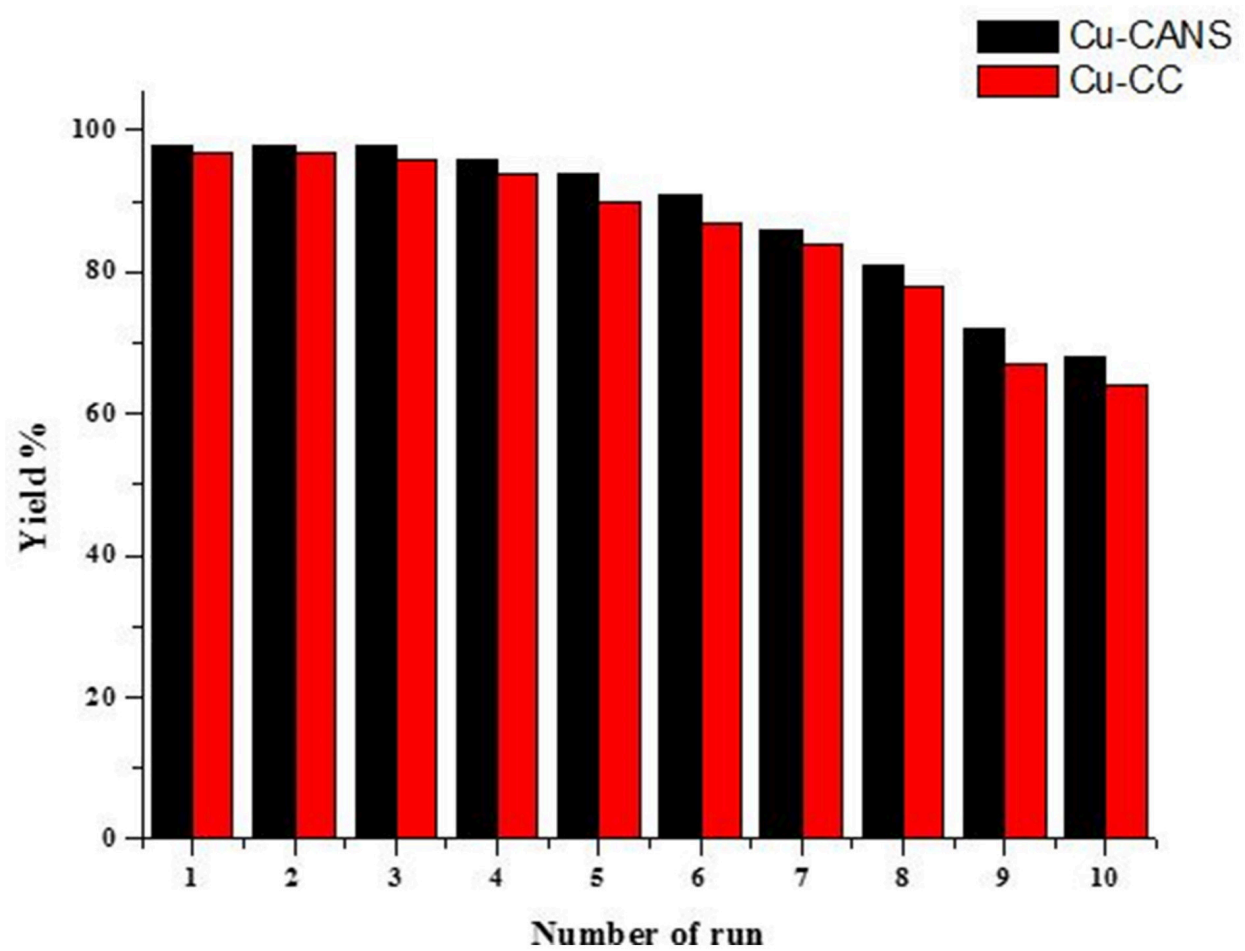
Recycling results of the Cu-CC and Cu-CANS catalytic systems in the copper-catalyzed cycloaddition reaction of phenylacetylene and benzyl azide.

Generally copper(I) catalysts are unstable and can readily oxidize to copper(II). However, it is legitimate to question also the stability of Cu-carbon entities as reagents in organic reactions. The conservation of the copper(I)-carbon catalysts under non-inert conditions (under air), did not cause any change in their external aspects and it has been observed, unexpectedly, that after 1 year nothing has been lost of their activities. This heterogeneous catalytic system offers easy manipulation and separation by simple filtration, thus facilitating the recycling of the copper-carbon catalysts. The recyclability of copper-carbon was tested in the model cycloaddition reaction of phenylacetylene and benzyl azide (Figure 7). After completion of the cycloaddition reaction in water at room temperature, the catalyst was recovered by simple filtration and reused after washing with diethyl ether and drying in the air. The catalyst was reused directly for the next run under the same conditions. This process was performed until 10 times without any significant loss of efficiency and selectivity of the Cu-carbon as shown by the catalytic histogram in Figure 7.

The percentages of the copper contents of the fresh and recycled Cu-CC and Cu-CANS after the first and the eight consecutive trial were determined by AAS analysis. The AAS results shown in Table 5 indicate that the weight percentage of the copper contents of the recycled catalysts after the first cycles is around 3.65 wt% for Cu-CC and 1.01 wt% for Cu-CANS, values which are lower than those of the fresh catalyst, 3.82 and 1.38 wt%, respectively. After the eight catalytic trial, the percentages of the copper content were found to be around 0.82 wt% for Cu-CC and 0.58 wt% for Cu-CANS. The decrease of the 1,2,3-triazole products yields is caused by the loss of the catalytically active species copper(I) during the work-up processes.

**Table 5:** Loadings of copper in each 100 mg of copper-carbon catalyst.

**Catalyst**	**Copper loading (wt%)**		
	**Before reaction**	**After the first cycle**	**After the eighth cycle**
Cu-CC	3.82	3.65	0.82
Cu-CANS	1.38	1.09	0.58

### Comparison of the Copper-Carbon Catalysts With Other Heterogeneous Catalysts Containing Copper Particles

In principle, any efficient prepared catalytic system is expected be superior to the commercially available catalysts used for the same purpose. Taking into account this principle, it seemed important to compare also the catalytic activity of the copper(I)-carbon with that of other copper-containing heterogeneous catalysts among the most active known for this type of cycloaddition reaction such as Cu-charcoal, Cu_2_O-C, copper-graphene, copper-alginate, copper-chitine, copper-hydroxyappatite, copper-argile, and copper-resin (Table 6). The series of catalysts used proved to be effective since the product was obtained with excellent yields in all the cases. However, the total conversion of the substrates was possible only after 15–18 h of reaction, proving the lower reactivity of these catalysts in this type of reaction. Remarkably, the Cu-CANS and Cu-CC catalysts have a higher activity, allowing the formation of the desired product using only 0.5 mol% during 6 h of reaction. In addition, this catalytic system has many advantages: it is robust, inexpensive, readily available, non-toxic, and has no sensitivity to humidity or air.

**Table 6:** Comparison of the catalytic activity of cooper-carbon catalysts with others heterogeneous copper-based catalytic systems.

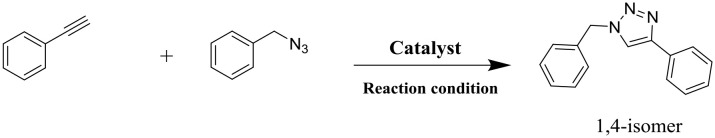							
**Entry**	**Catalyst**	**Time (h)**	**Cat. loading (mol %)**	**Temperature/solvent**	**Number of cycle**	**Yield (%)**	**Reference**
1	Cu-CC	6	1	r.t./water	10	98	This work
2	Cu-CANS	6	1	r.t./water	10	98	
3	Cu/C	48	10	23°C/dioxane	–	65	Lipshutz and Taft, 2006
4	Cu_2_O/C	2	5	r.t./i-PrOH:H_2_O	3	82	López-Ruiz et al., 2012
5	TRGO/Cu	48	2	40°C/THF	4	99	Shaygan Nia et al., 2014
6	Cu-Alginate	18	21	r.t./water	3	98	Rajender Reddy et al., 2007
7	Cu-Chitosan	6	10	r.t./water	5	90	Anil Kumar et al., 2015
8	Cu-Hydroxyappatite	16	5	50°C/water	8	95	Masuyama et al., 2011
9	Cu-zeolite	15	10	r.t./toluene	5	83	Chassaing et al., 2007

## Conclusions

In summary, we have successfully developed a highly efficient and recyclable inexpensive copper on carbon heterogeneous catalyst for the regioselective construction of 1,4-disubstituted 1,2,3-triazoles by the [3+2] cycloaddition reactions of azides with alkynes under very strict click chemistry conditions with a sustainable fashion. The copper on carbon Cu-CANS was readily prepared by the copper(I) impregnation of carbon material that has been made from a naturally raw vegetable biomass. A wide range of azides and alkynes can be combined to form the important biologically active 1,2,3-triazoles by using Cu-CANS in water as solvent at room temperature. Cu-CANS was recycled and reused for several catalytic trials without noticeable loss of its catalytic activity. The use of benign water as solvent at room temperature makes the entire catalytic protocol an environmental friendly one.

## Author Contributions

S-ES and HB designed the project and supervised the synthetic and characterization chemistry works. NA and LB undertook all the synthetic experimental works and characterizations of the copper on carbon material catalysts. EE and SR carried out the synthesis of the natural carbon material originated from vegetable biomass using *Argan nut shells* (CANS). SR performed the characterization of the carbon precursor material CANS. S-ES, HB, MJ, SR, and HA contributed to the writing of the manuscript and interpretation of the results. MJ supplied the funding for the work.

### Conflict of Interest Statement

The authors declare that the research was conducted in the absence of any commercial or financial relationships that could be construed as a potential conflict of interest.
