# Supplementary material for: Sustainable Construction of Heterocyclic 1,2,3-Triazoles by Strict Click [3+2] Cycloaddition Reactions Between Azides and Alkynes on Copper/Carbon in Water
Source: Front Chem. 2019 Feb 19;7:81. doi: 10.3389/fchem.2019.00081 (PMC6389623; doi:10.3389/fchem.2019.00081)
Supplement: Supplementary file 1 [file Data_Sheet_1.pdf]

Supplementary Material

Sustainable Construction of Heterocyclic 1,2,3-Triazoles by Strict Click [3+2] Cycloaddition Reactions Between Azides and Alkynes on Copper/Carbon in Water

Noura Aflak[1], Hicham Ben El Ayouchia[1,*], Lahoucine Bahsis[1], El Mountassir El Mouchtari[1], Miguel Julve[2], Salah Rafqah[1], Hafid Anane[1],and Salah-Eddine Stiriba [1,2*]

[1]Laboratoire de Chimie Analytique et Moléculaire, LCAM, Faculté Polydisciplinaire de Safi, Université Cadi Ayyad, Safi 46030, Morocco.

[2]Instituto de Ciencia Molecular /ICMol, Universidad de Valencia, C/ Catedrático José Beltrán 2, 46980 Valencia, Spain.

*** Correspondence:** Salah-E. Stiriba: E-mail: stiriba@uv.es and Hicham Ben El Ayouchia: E-mail: belayou@gmail.com

^{1}H, ^{13}C and Dept-135 NMR spectra of all 1,2,3-triazoles obtained in this work, as well as their mass spectra (**2S-45S**)

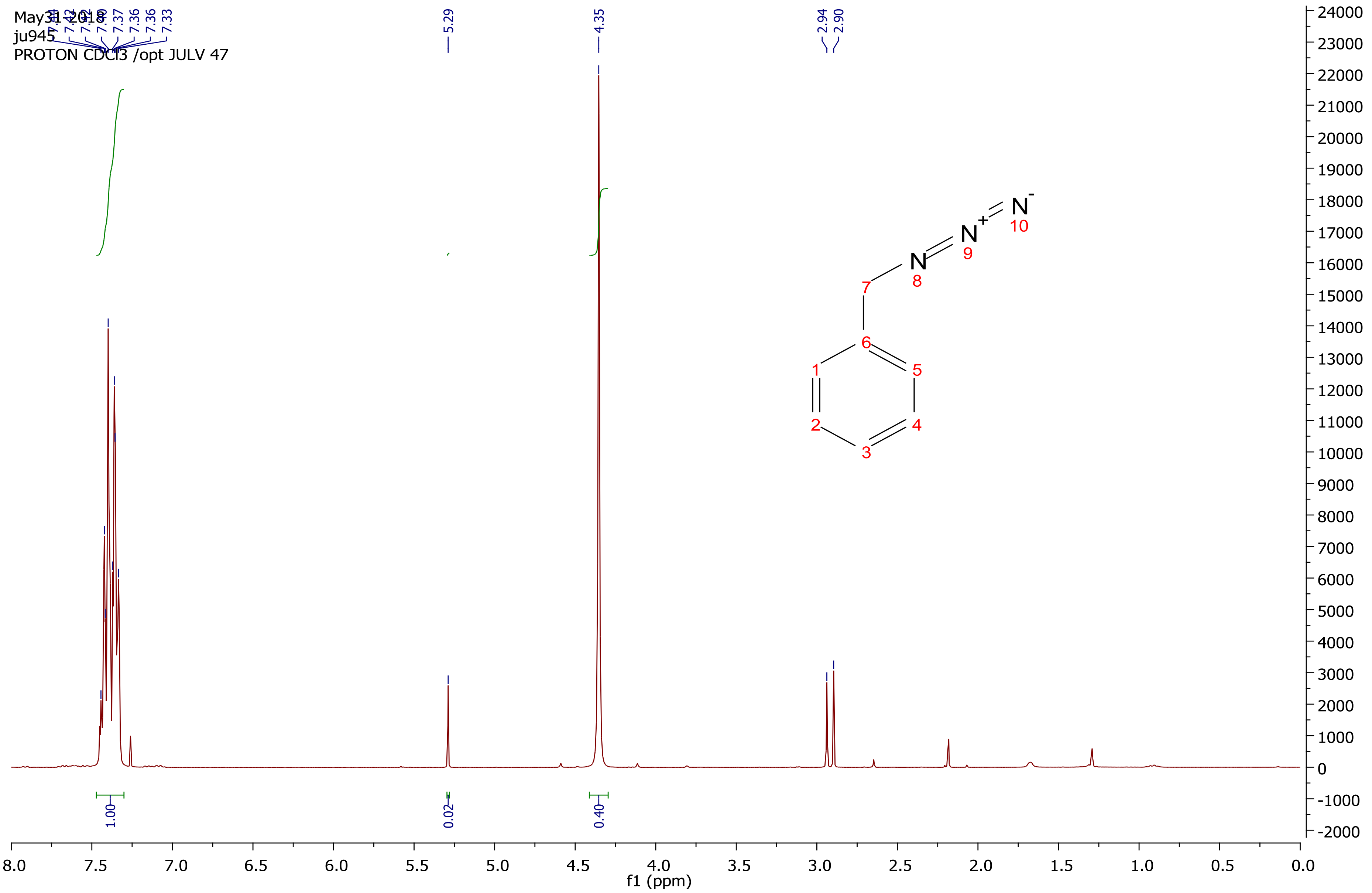
May31-2018
ju945
PROTON CDCl3 /opt JULV 47
7.44
7.42
7.42
7.40
7.37
7.36
7.36
7.33
5.29
4.35
2.94
2.90
1.00
0.02
0.40
f1 (ppm)
N
N+
N-
1
2
3
4
5
6
7
8
9
10

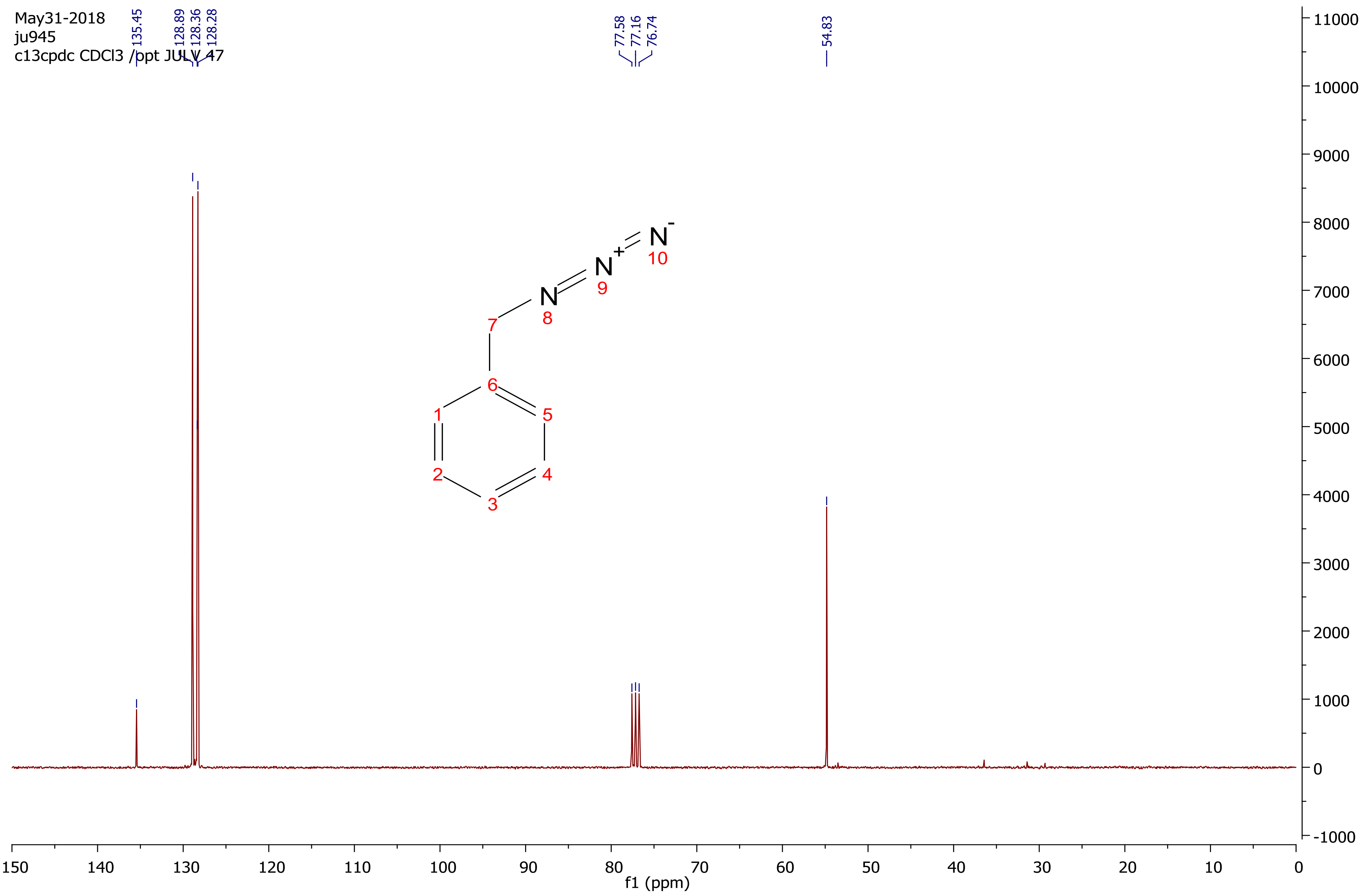
May31-2018
ju945
c13cpdc CDCl3 /ppt JULV 47
135.45
128.89
128.36
128.28
77.58
77.16
76.74
54.83
N
N
N
+
-
1
2
3
4
5
6
7
8
9
10
f1 (ppm)

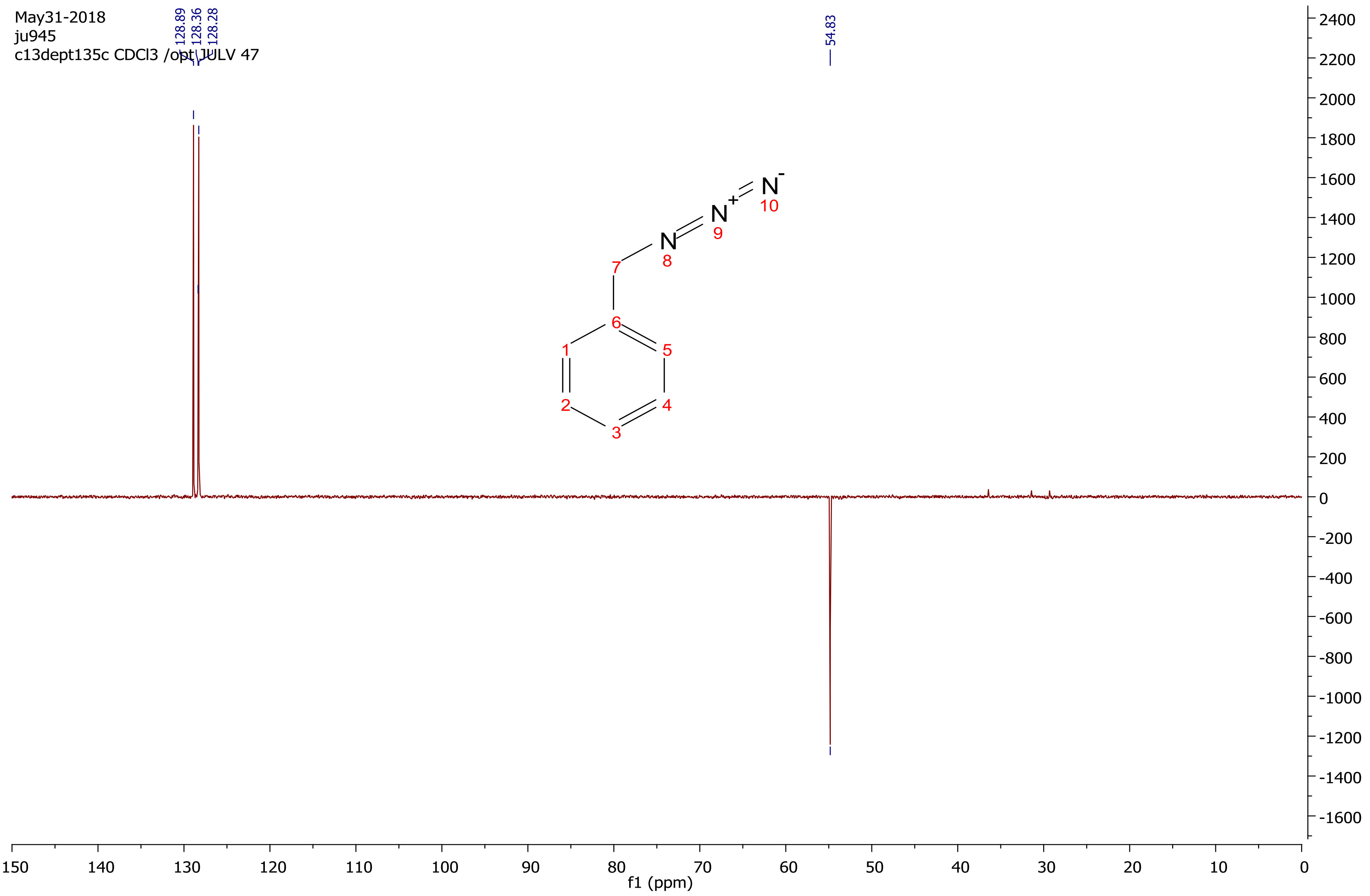
May31-2018
ju945
c13dept135c CDCl3 /opt JULV 47
128.89
128.36
128.28
54.83
N
N+
N-
1
2
3
4
5
6
7
8
9
10
f1 (ppm)

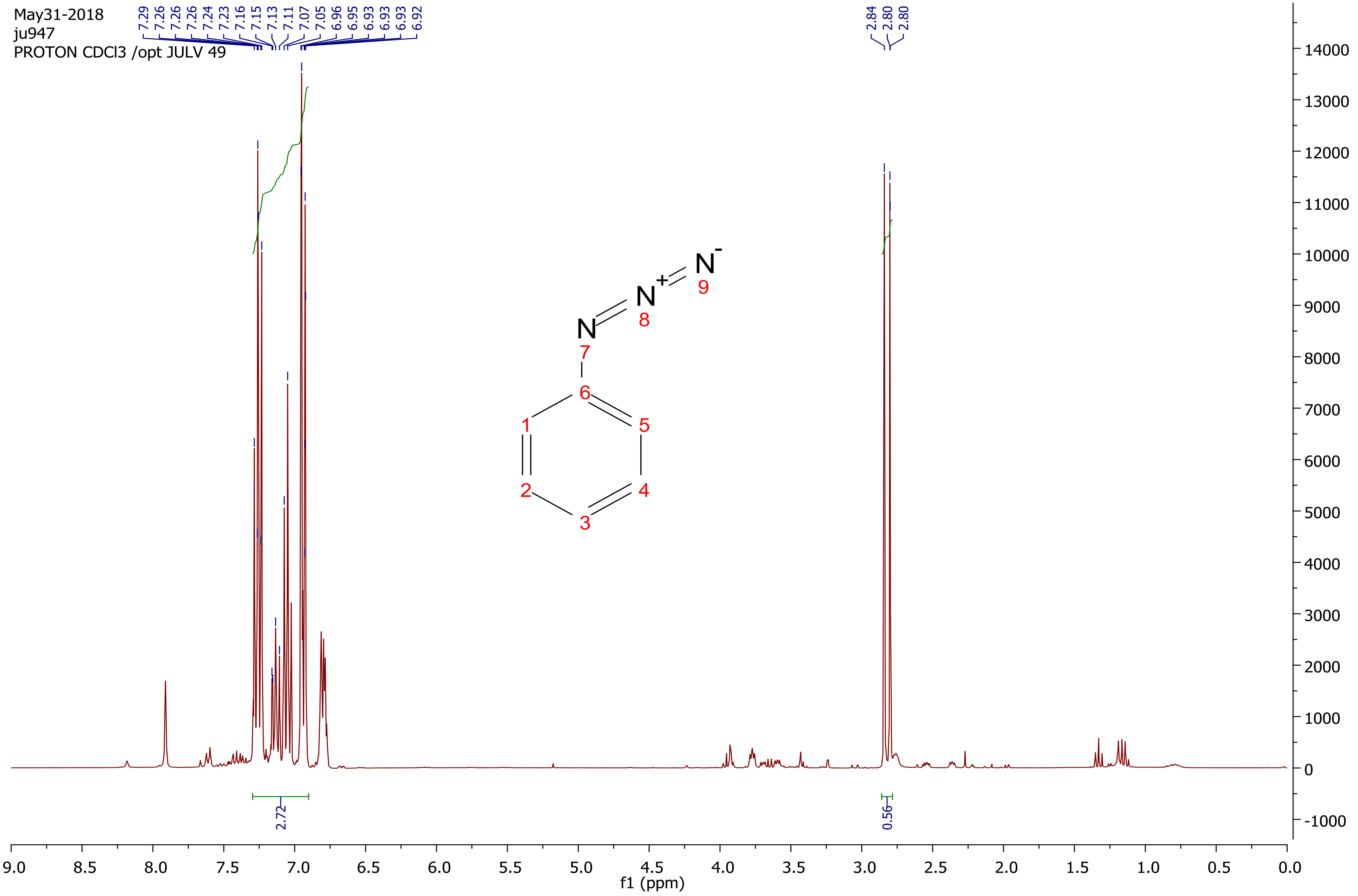

May31-2018
ju947
PROTON CDCl3 /opt JULV 49
7.29
7.26
7.26
7.26
7.24
7.23
7.16
7.15
7.13
7.11
7.07
7.05
6.96
6.95
6.93
6.93
6.93
6.92
2.84
2.80
2.80
N
N+
N-
1
2
3
4
5
6
7
8
9
2.72
0.56
f1 (ppm)

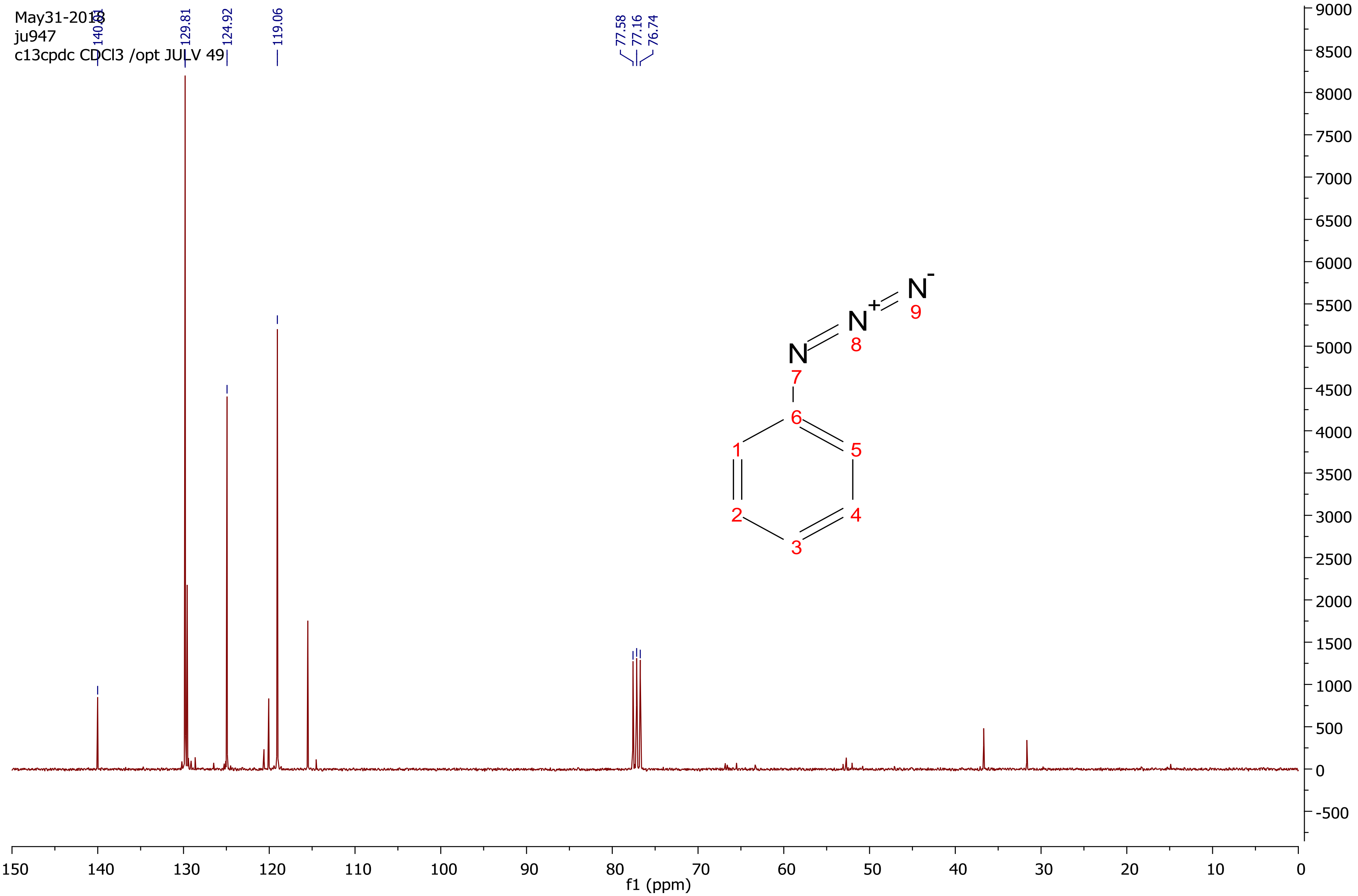
May31-2018
ju947
c13cpdc CDCl3 /opt JULV 49
140.01
129.81
124.92
119.06
77.58
77.16
76.74
N
7
N
8
+
N
9
-
6
1
5
2
4
3
f1 (ppm)

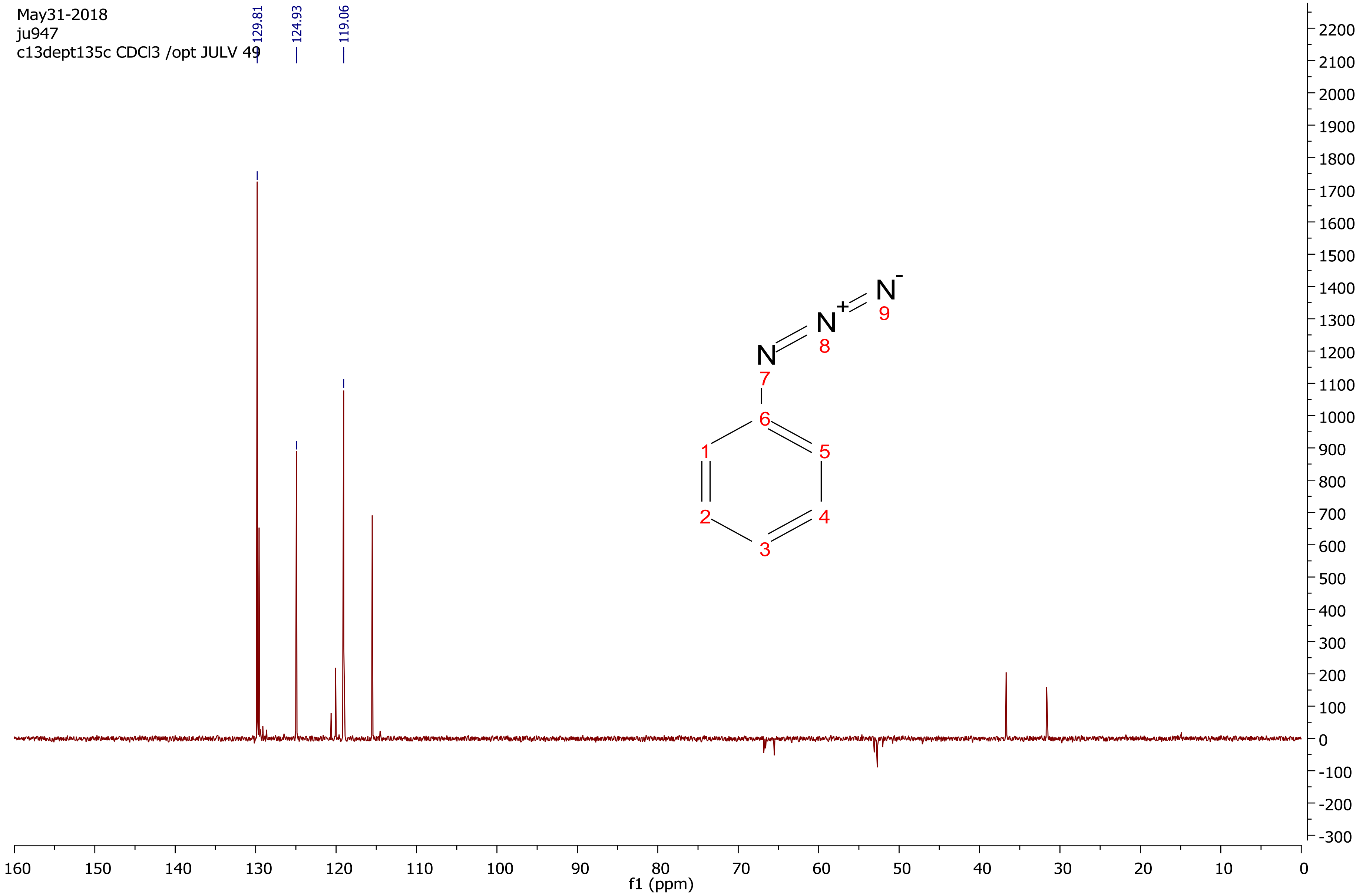
May31-2018
ju947
c13dept135c CDCl3 /opt JULV 49
129.81
124.93
119.06
N
N
N
+
-
1
2
3
4
5
6
7
8
9
f1 (ppm)

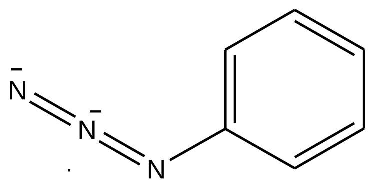

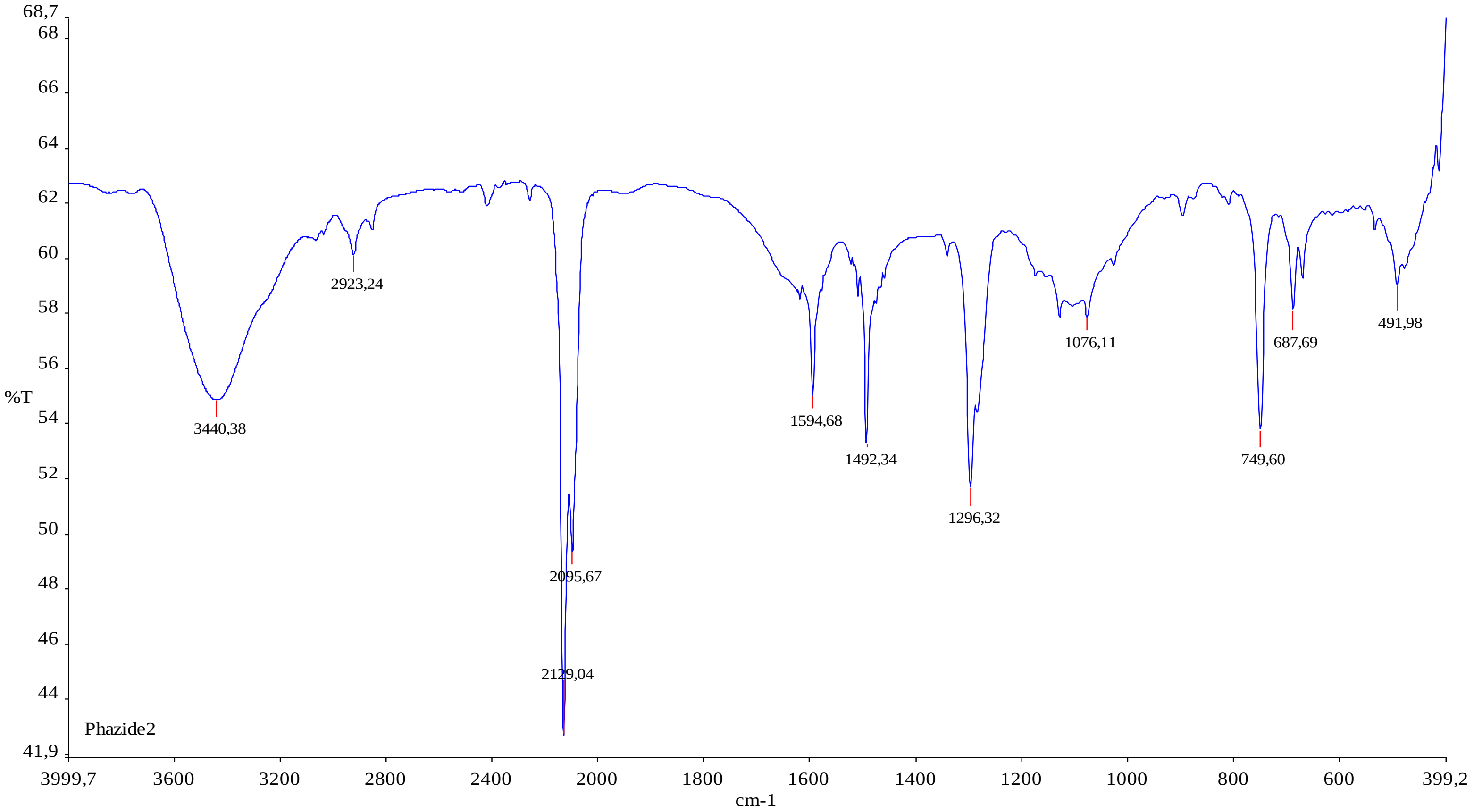

68,7
68
66
64
62
60
58
56
54
52
50
48
46
44
41,9
%T
3440,38
2923,24
2129,04
2095,67
1594,68
1492,34
1296,32
1076,11
749,60
687,69
491,98
Phazide2
3999,7
3600
3200
2800
2400
2000
1800
1600
1400
1200
1000
800
600
399,2
cm-1

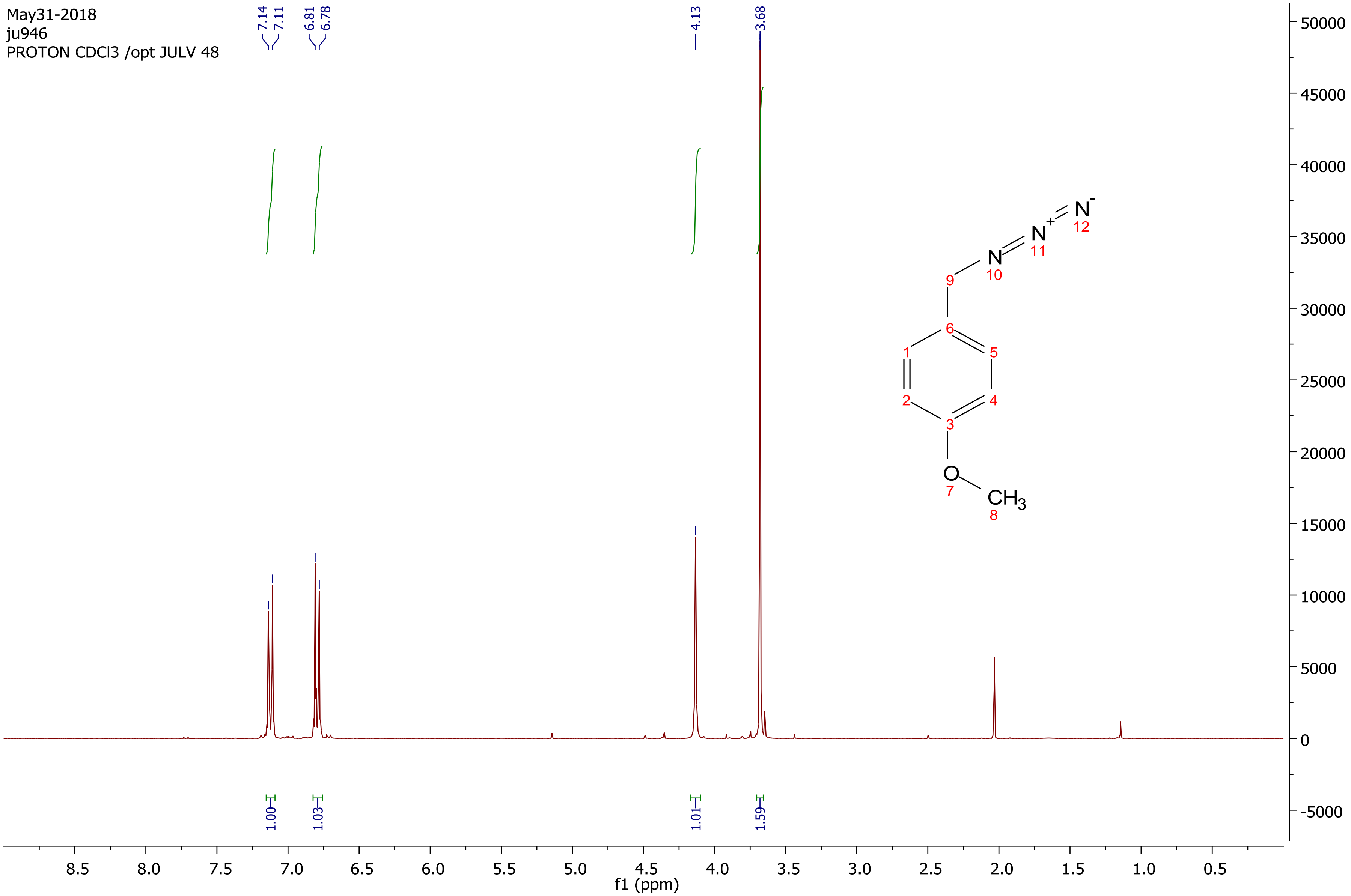
May31-2018
ju946
PROTON CDCl3 /opt JULV 48
7.14
7.11
6.81
6.78
4.13
3.68
1.00
1.03
1.01
1.59
f1 (ppm)
N
N+
N-
CH3
O

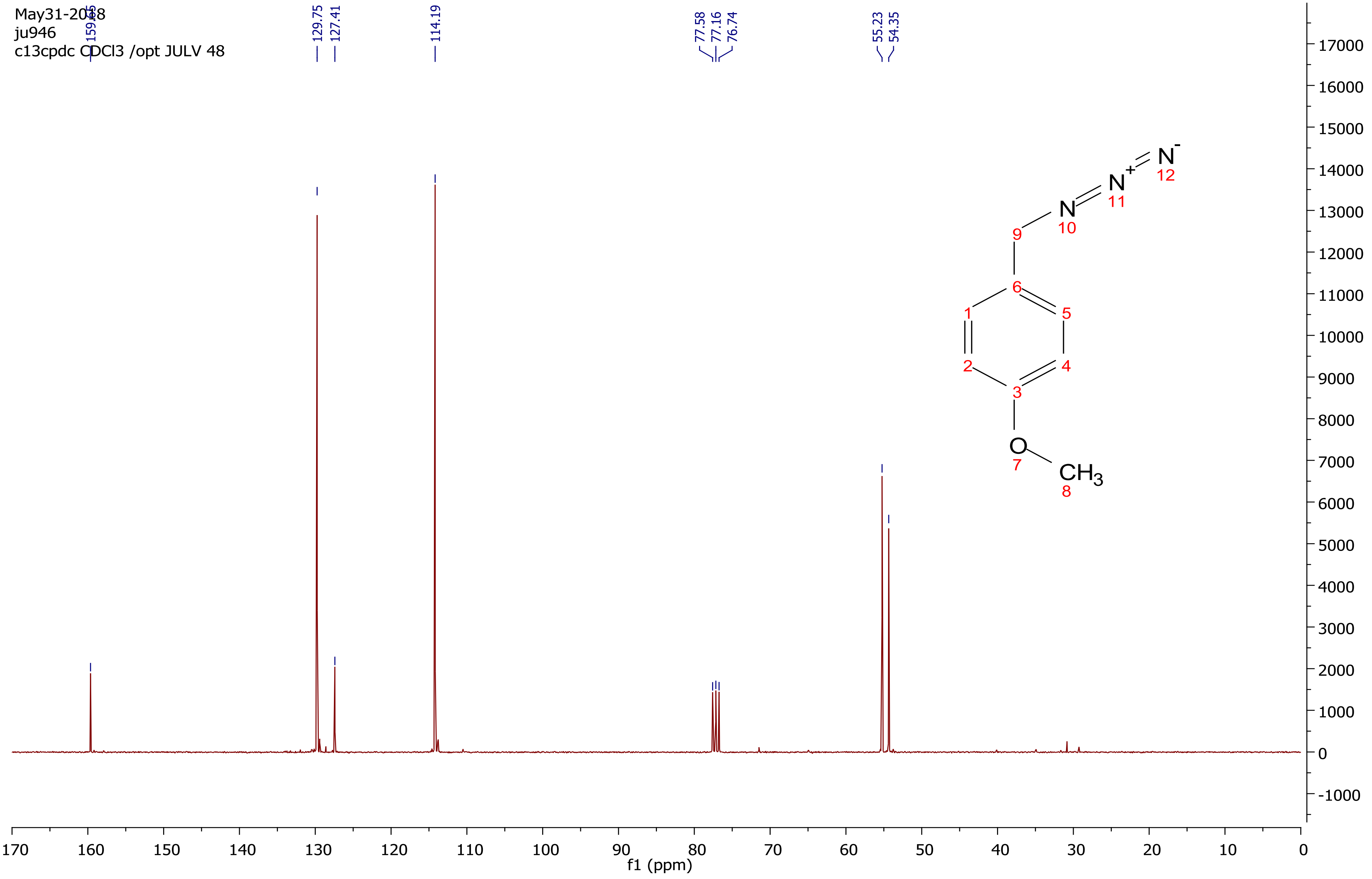
May31-2018
ju946
c13cpdc CDCl3 /opt JULV 48
159.65
129.75
127.41
114.19
77.58
77.16
76.74
55.23
54.35
f1 (ppm)
N
N
N
CH3
O
1
2
3
4
5
6
7
8
9
10
11
12

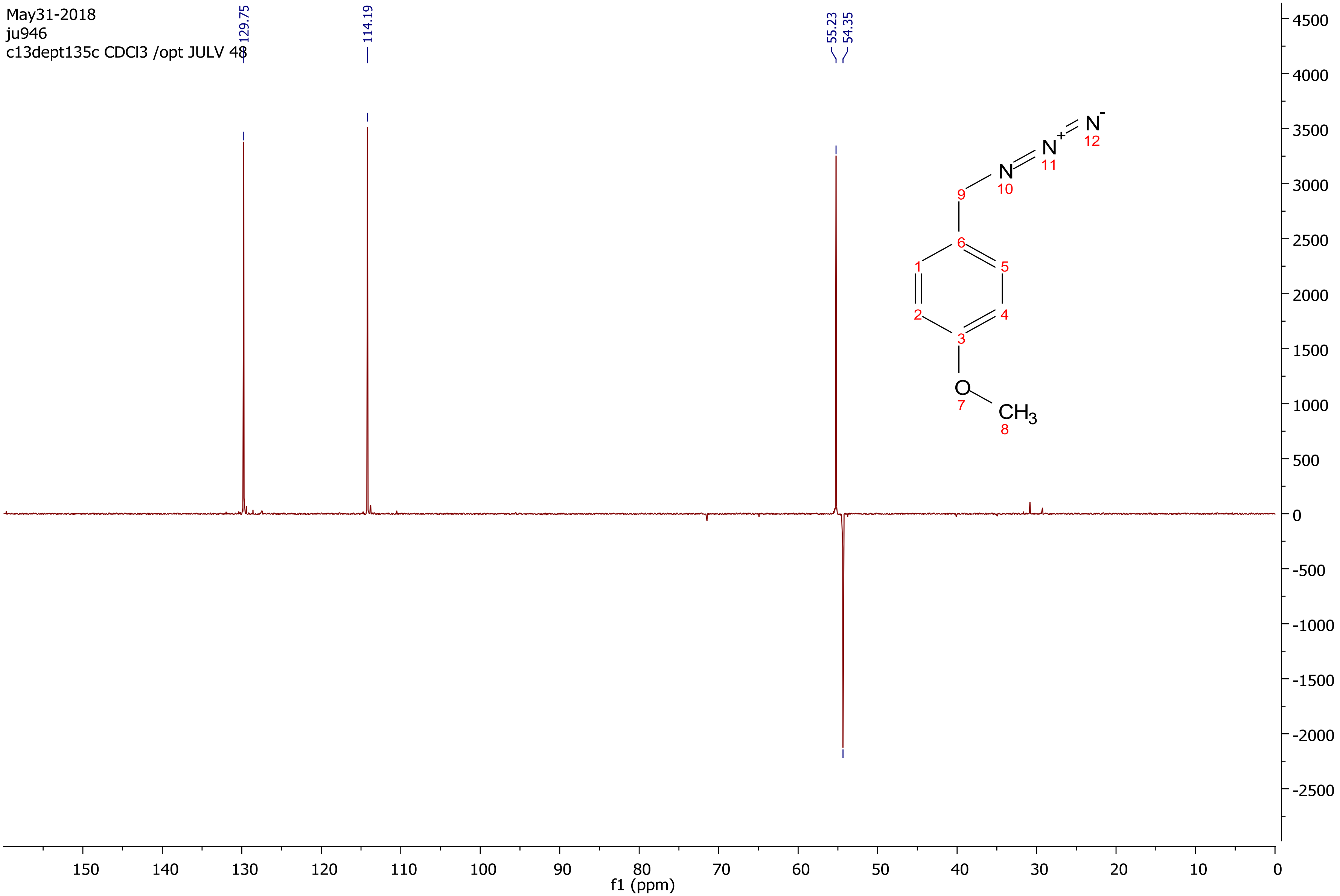

May31-2018
ju946
c13dept135c CDCl3 /opt JULV 48
129.75
114.19
55.23
54.35
N
N+
N-
1
2
3
4
5
6
7
8
9
10
11
12
O
CH3
4500
4000
3500
3000
2500
2000
1500
1000
500
0
-500
-1000
-1500
-2000
-2500
150
140
130
120
110
100
90
80
70
60
50
40
30
20
10
0
f1 (ppm)

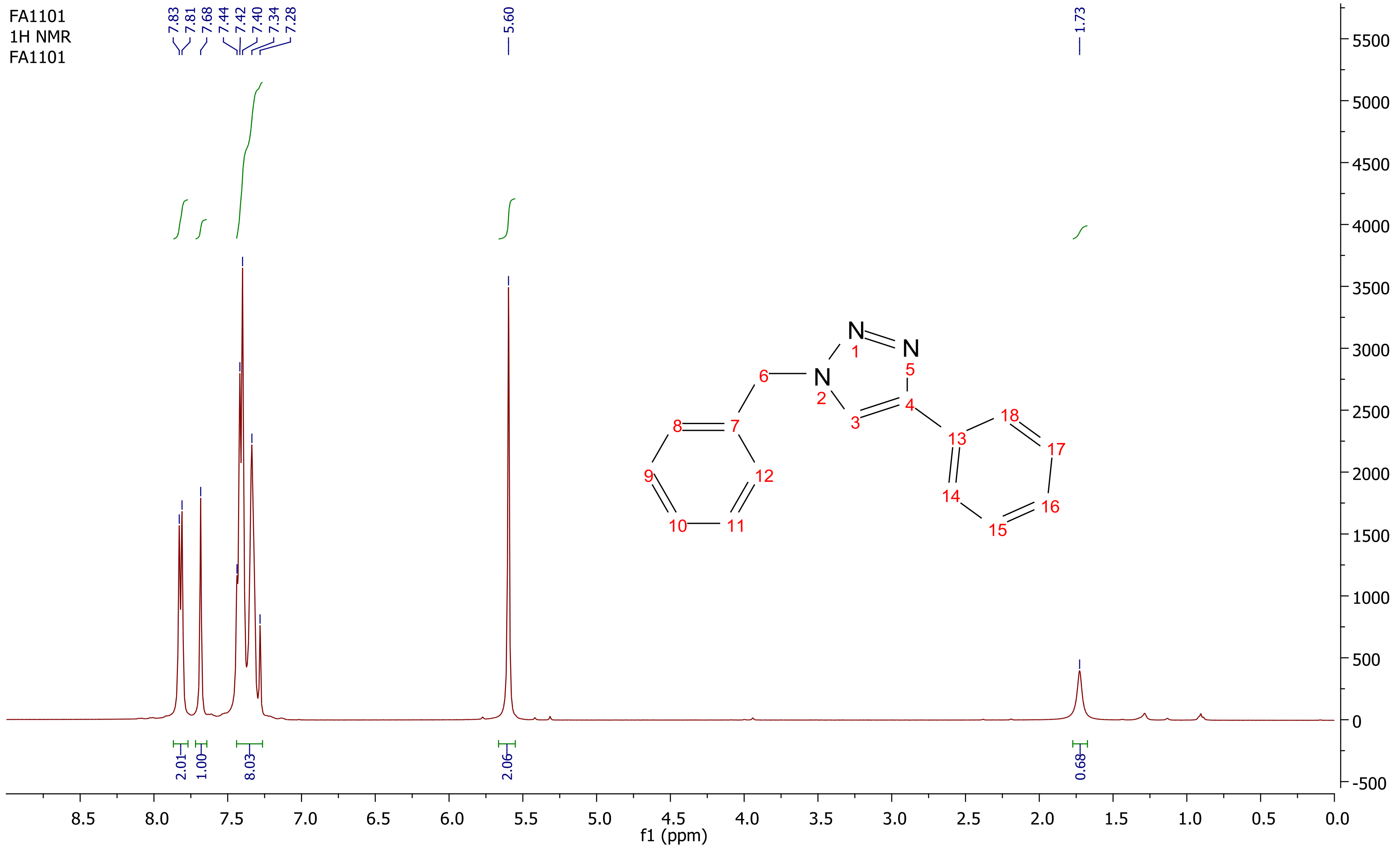
FA1101
1H NMR
FA1101
7.83
7.81
7.68
7.44
7.42
7.40
7.34
7.28
5.60
1.73
2.01
1.00
8.03
2.06
0.68
f1 (ppm)

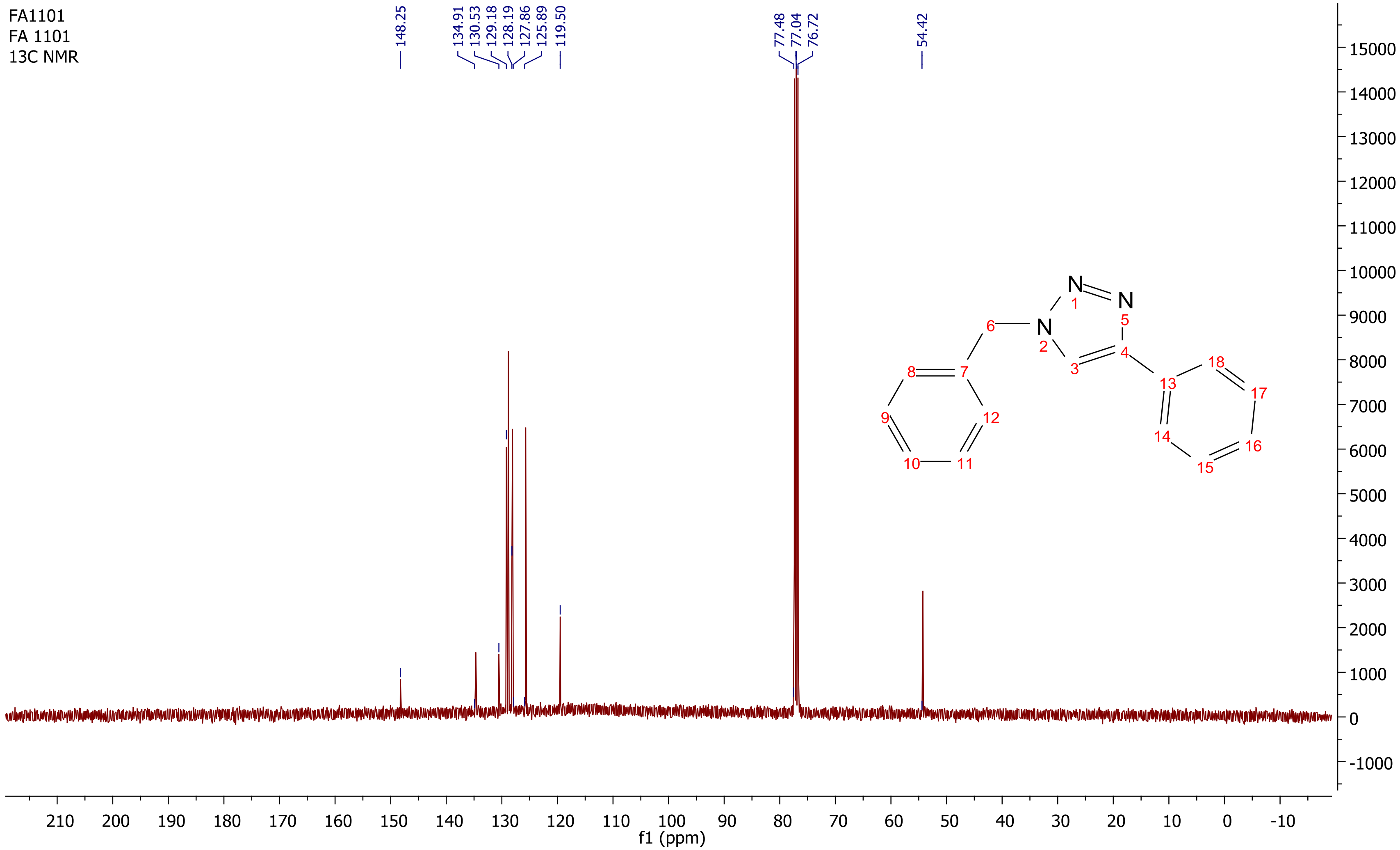
FA1101
FA 1101
13C NMR
148.25
134.91
130.53
129.18
128.19
127.86
125.89
119.50
77.48
77.04
76.72
54.42
f1 (ppm)

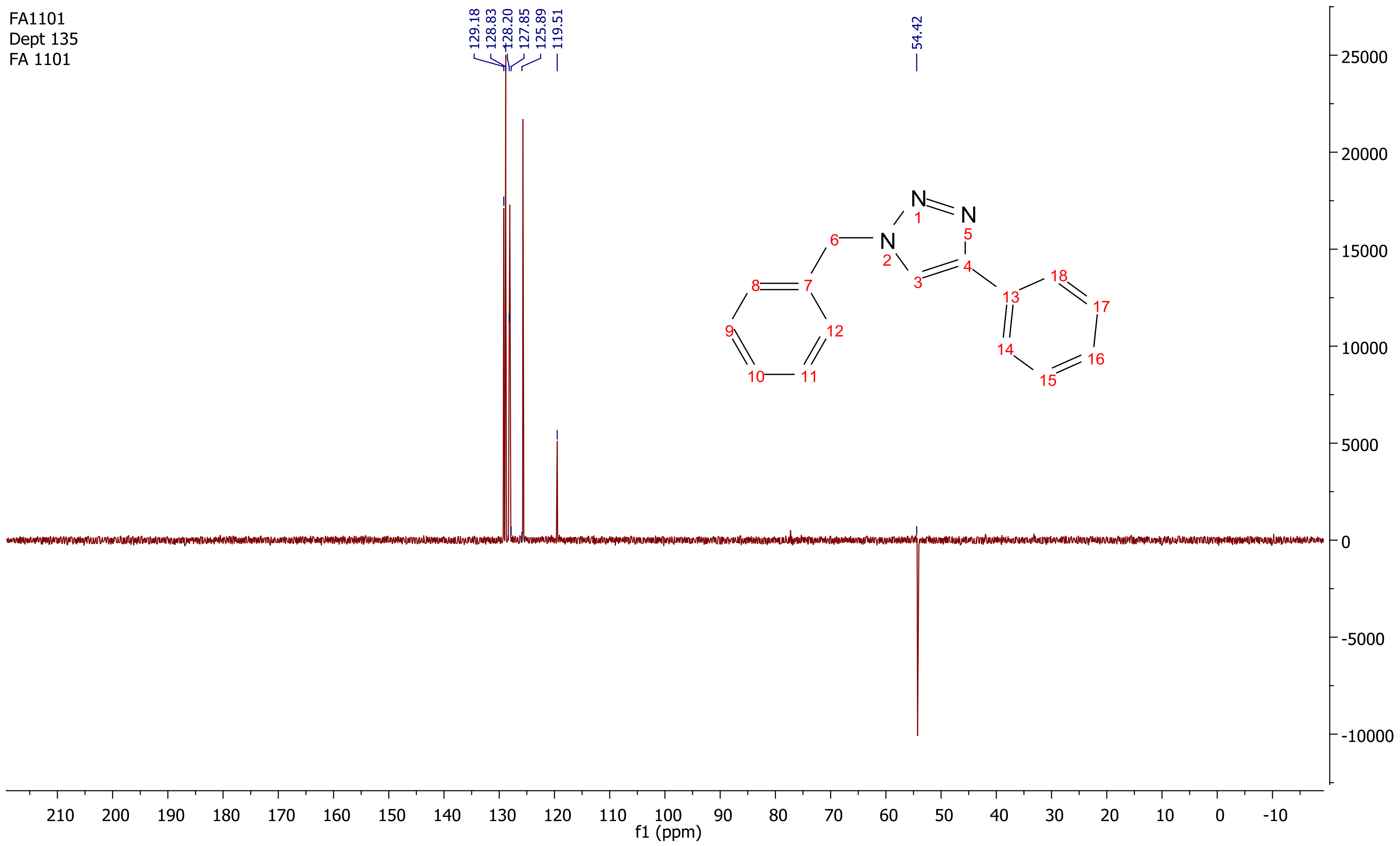
FA1101
Dept 135
FA 1101
129.18
128.83
128.20
127.85
125.89
119.51
54.42
f1 (ppm)

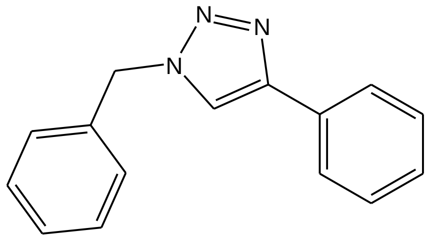
N
N
N

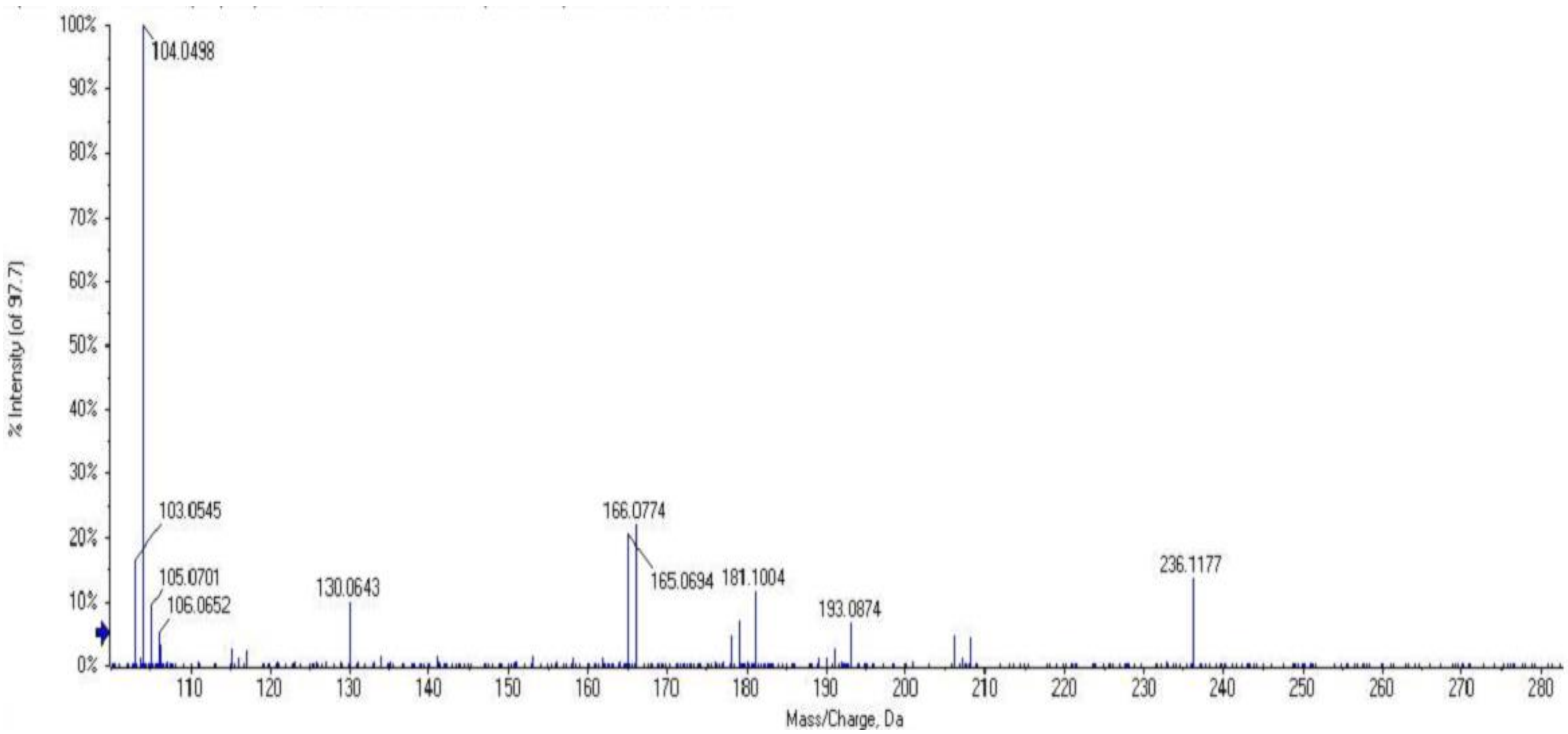
100%
90%
80%
70%
60%
50%
40%
30%
20%
10%
0%
% Intensity (of 97.7)
104.0498
103.0545
105.0701
106.0652
130.0643
166.0774
165.0694
181.1004
193.0874
236.1177
110
120
130
140
150
160
170
180
190
200
210
220
230
240
250
260
270
280
Mass/Charge, Da

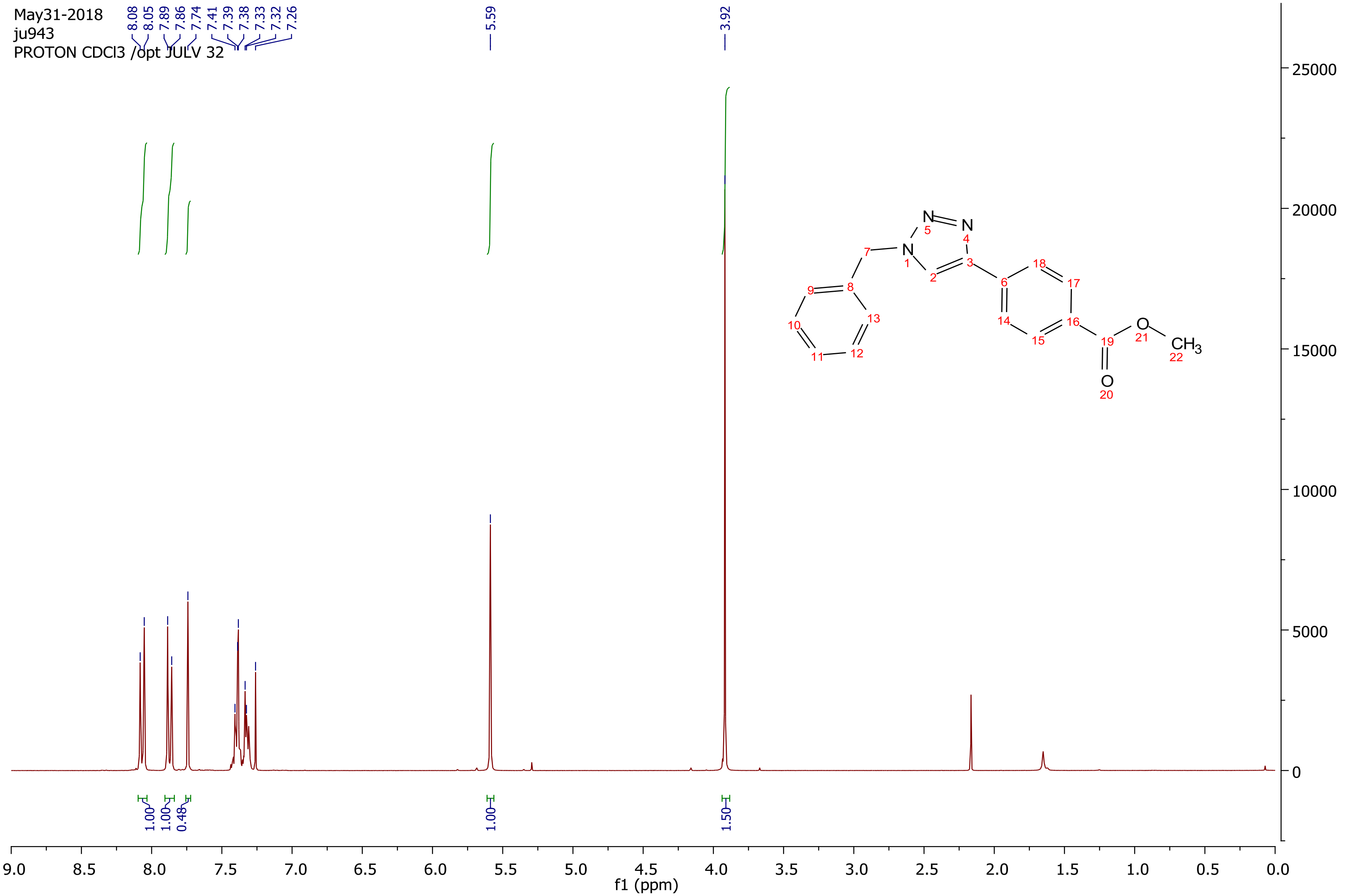
May31-2018
ju943
PROTON CDCl3 /opt JULV 32
8.08
8.05
7.89
7.86
7.74
7.41
7.39
7.38
7.33
7.32
7.26
5.59
3.92
1.00
1.00
0.48
1.00
1.50
25000
20000
15000
10000
5000
0
9.0
8.5
8.0
7.5
7.0
6.5
6.0
5.5
5.0
4.5
4.0
3.5
3.0
2.5
2.0
1.5
1.0
0.5
0.0
f1 (ppm)
N
N
N
O
O
CH3

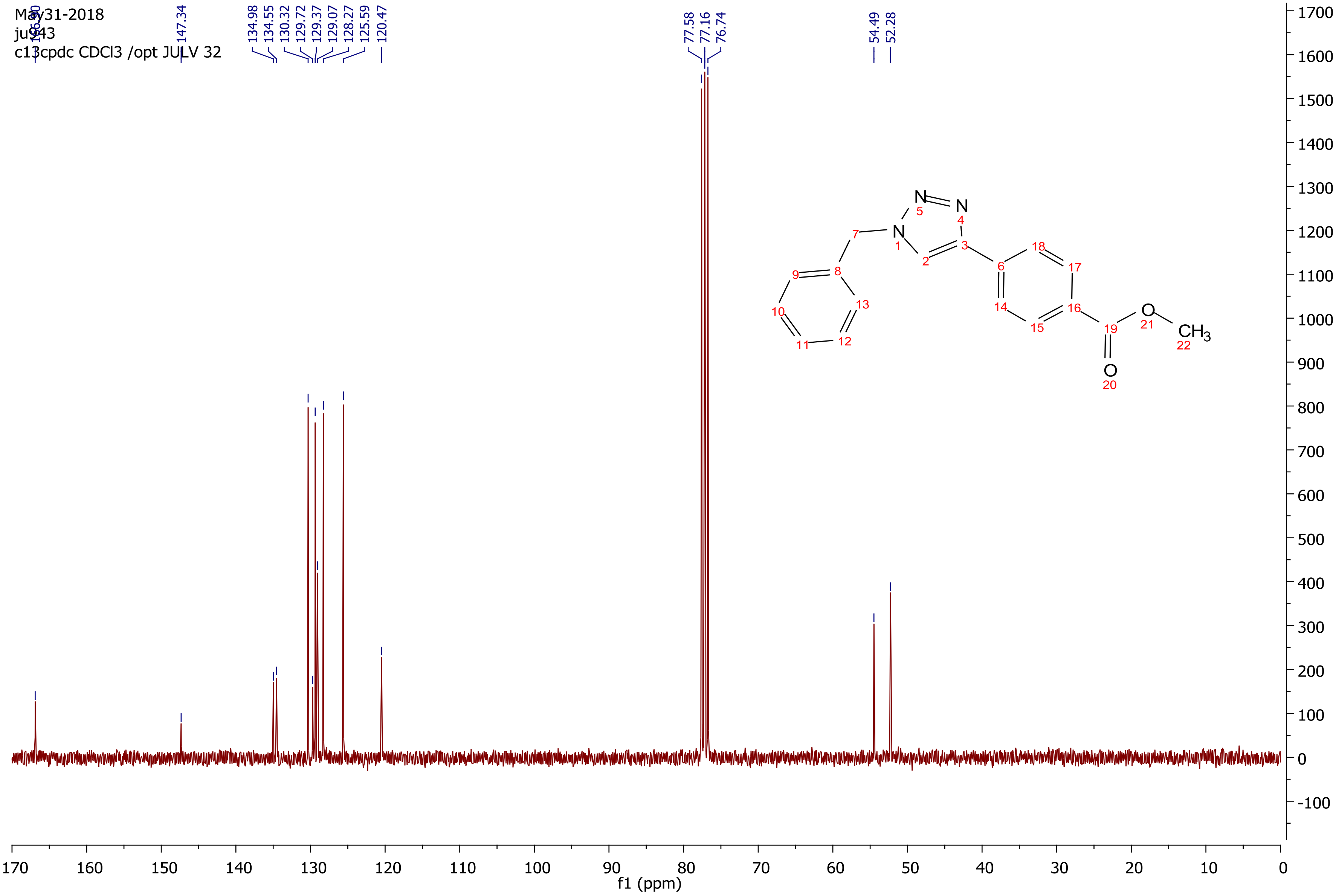

May31-2018
ju943
c13cpdc CDCl3 /opt JULV 32
166.90
147.34
134.98
134.55
130.32
129.72
129.37
129.07
128.27
125.59
120.47
77.58
77.16
76.74
54.49
52.28
N
N
N
5
4
7
1
3
2
9
8
18
17
6
13
10
14
16
O
15
19
21
CH3
11
12
22
O
20
1700
1600
1500
1400
1300
1200
1100
1000
900
800
700
600
500
400
300
200
100
0
-100
170
160
150
140
130
120
110
100
90
80
70
60
50
40
30
20
10
0
f1 (ppm)

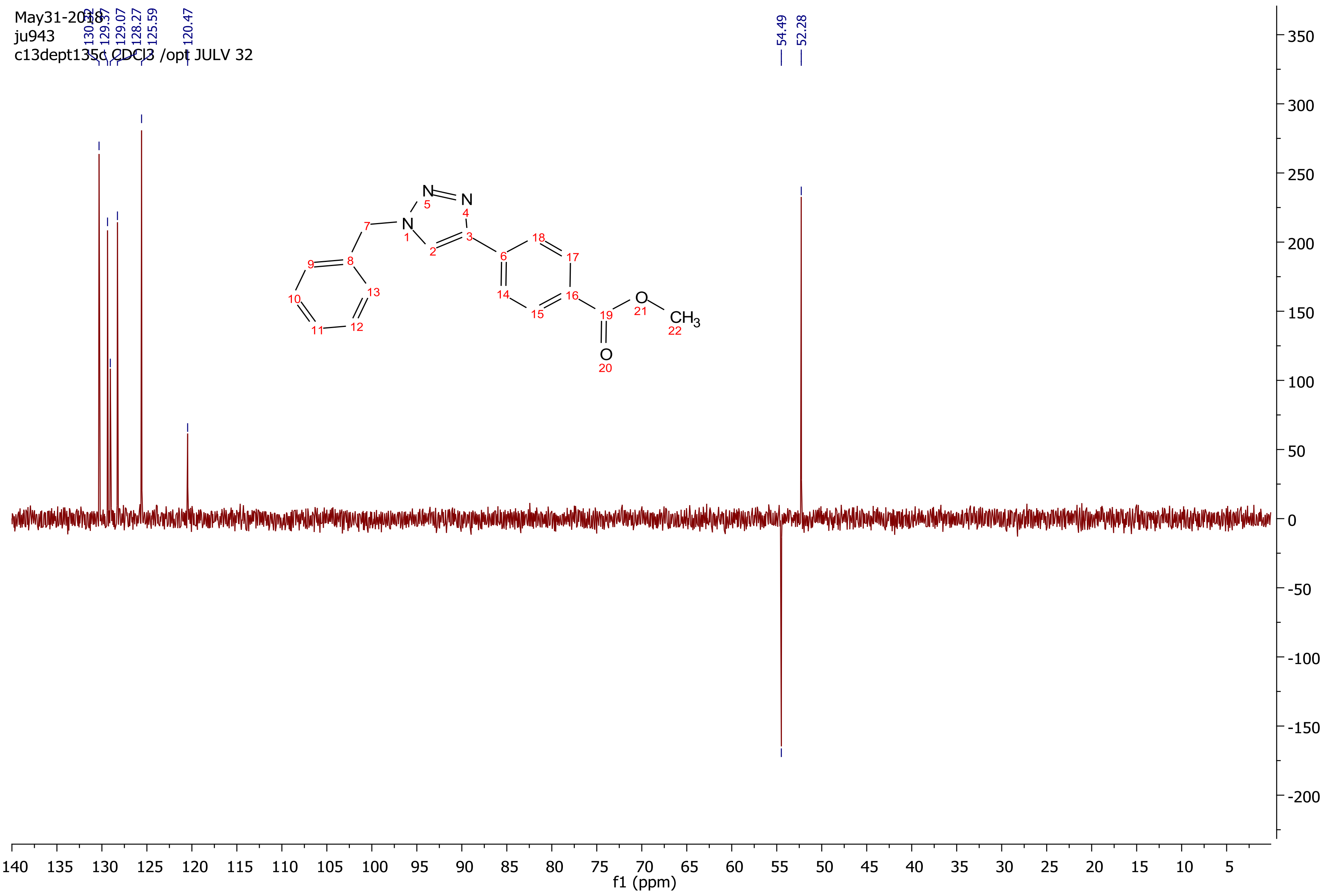
May31-2018
ju943
c13dept135c CDCl3 /opt JULV 32
130.32
129.37
129.07
128.27
125.59
120.47
54.49
52.28
N
N
N
CH3
O
O
f1 (ppm)

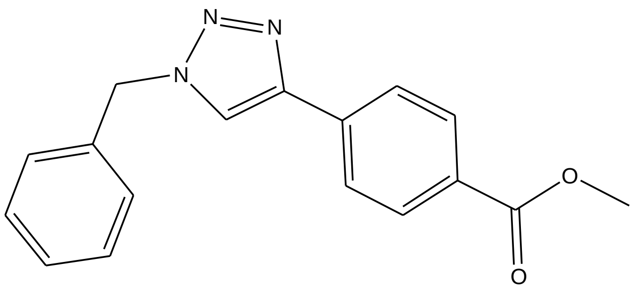
N
N
N
O
O

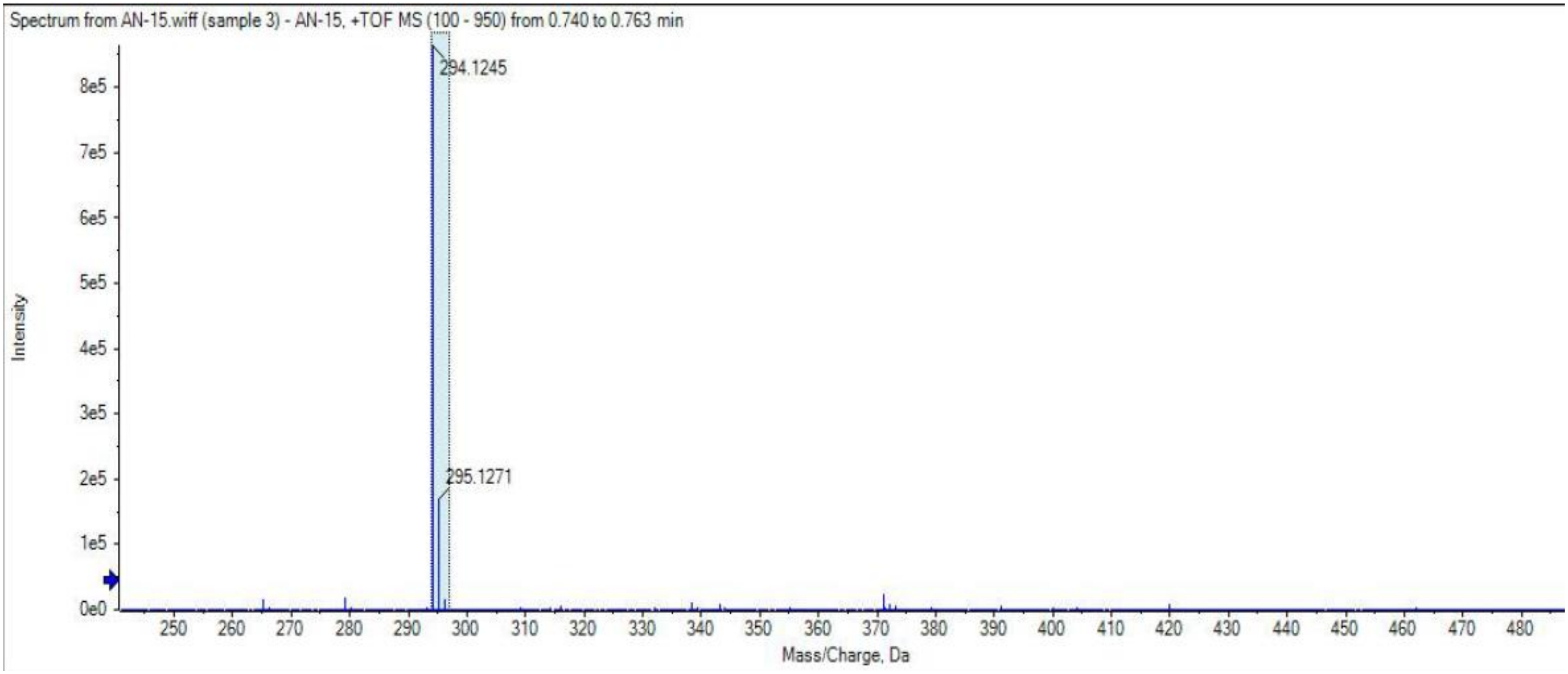
Spectrum from AN-15.wiff (sample 3) - AN-15, +TOF MS (100 - 950) from 0.740 to 0.763 min
294.1245
295.1271
Intensity
Mass/Charge, Da

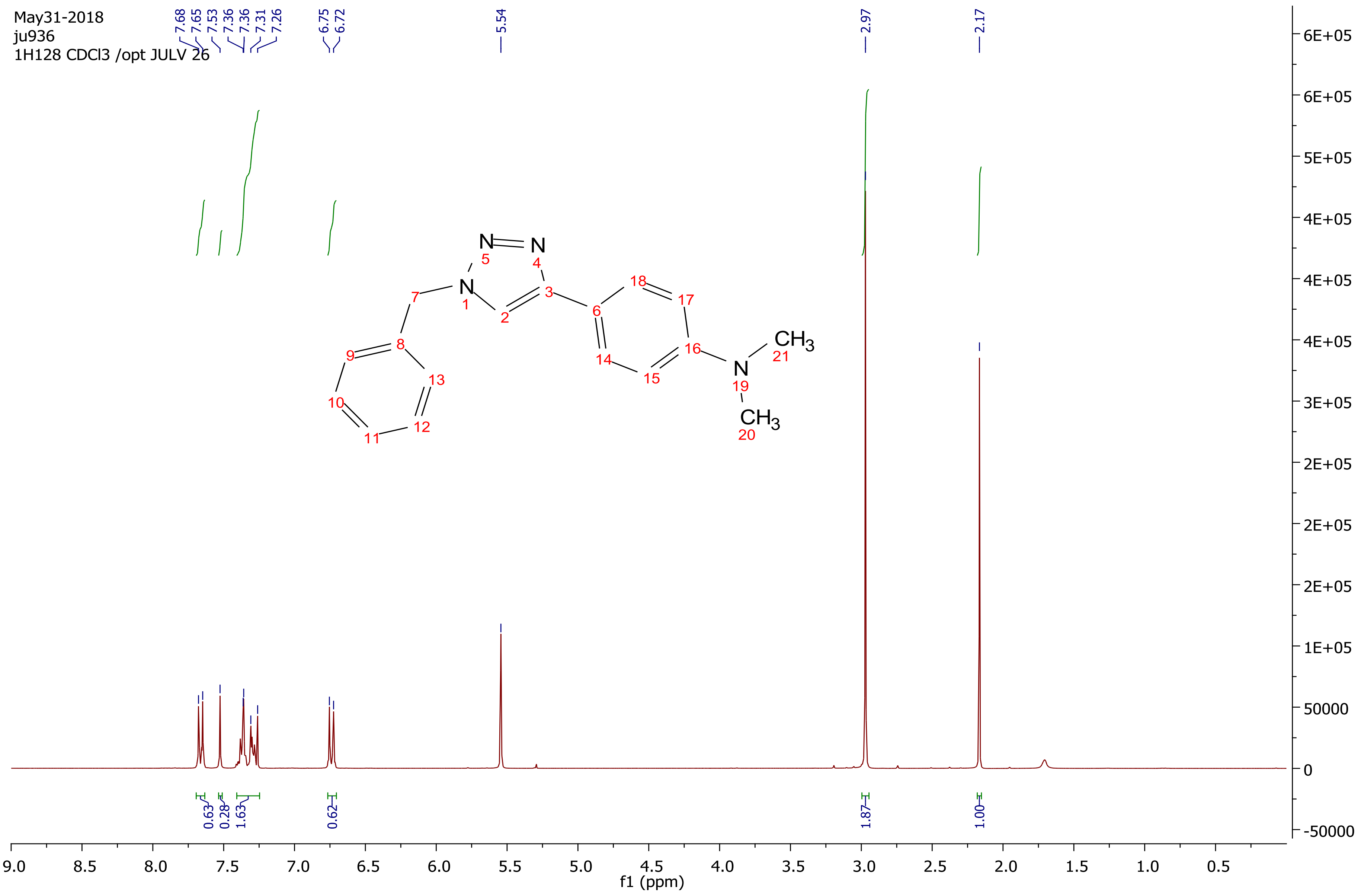
May31-2018
ju936
1H128 CDCl3 /opt JULV 26
7.68
7.65
7.53
7.36
7.36
7.31
7.26
6.75
6.72
5.54
2.97
2.17
0.63
0.28
1.63
0.62
1.87
1.00
f1 (ppm)
N
N
N
CH3
CH3

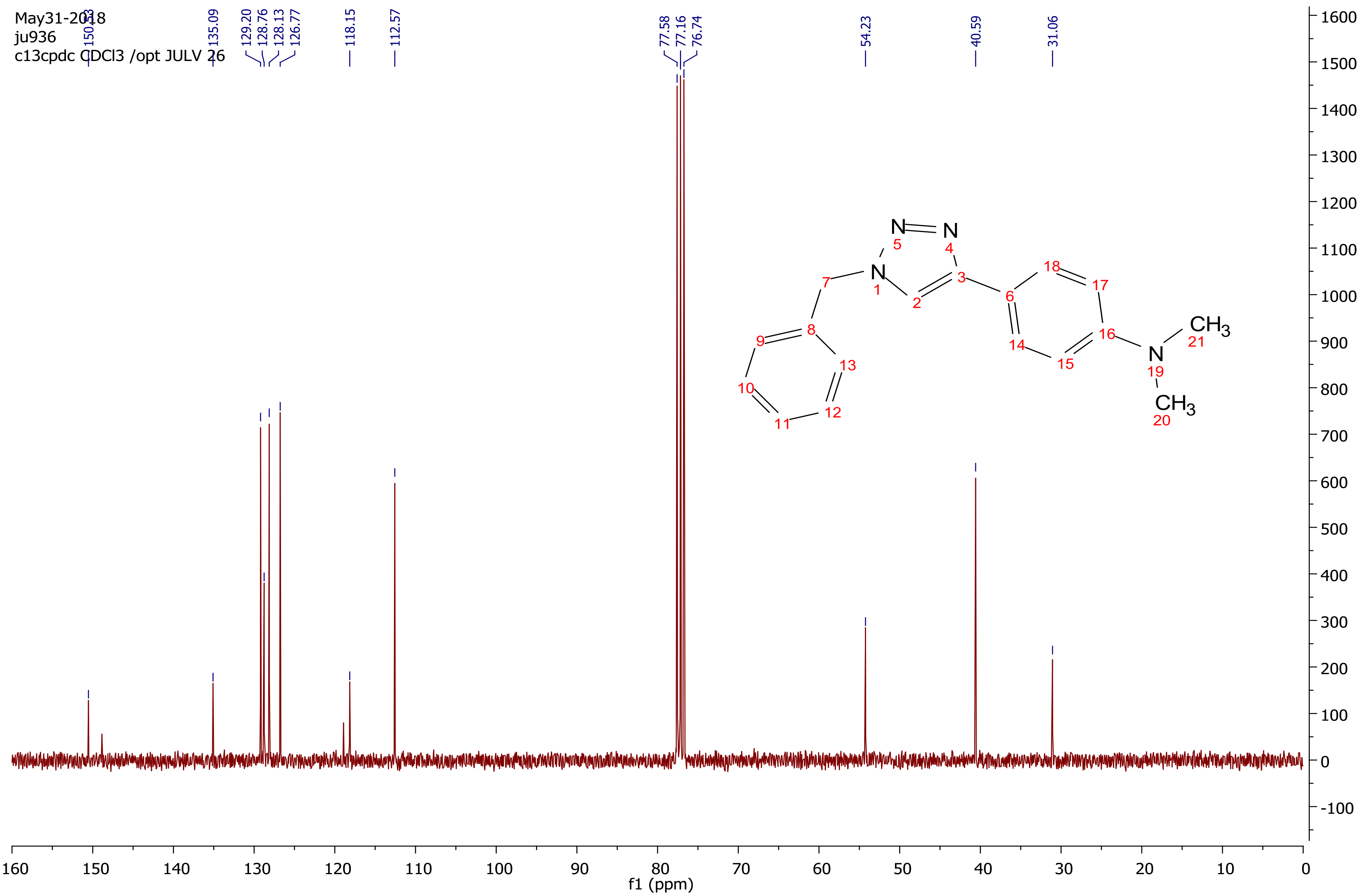
May31-2018
ju936
c13cpdc CDCl3 /opt JULV 26
150.13
135.09
129.20
128.76
128.13
126.77
118.15
112.57
77.58
77.16
76.74
54.23
40.59
31.06
f1 (ppm)

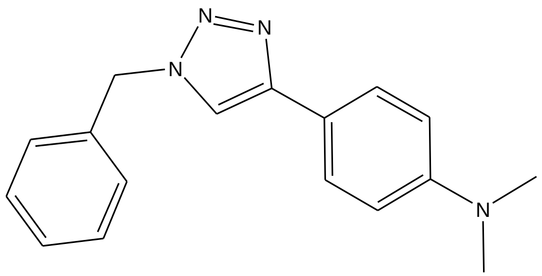
N
N
N
N

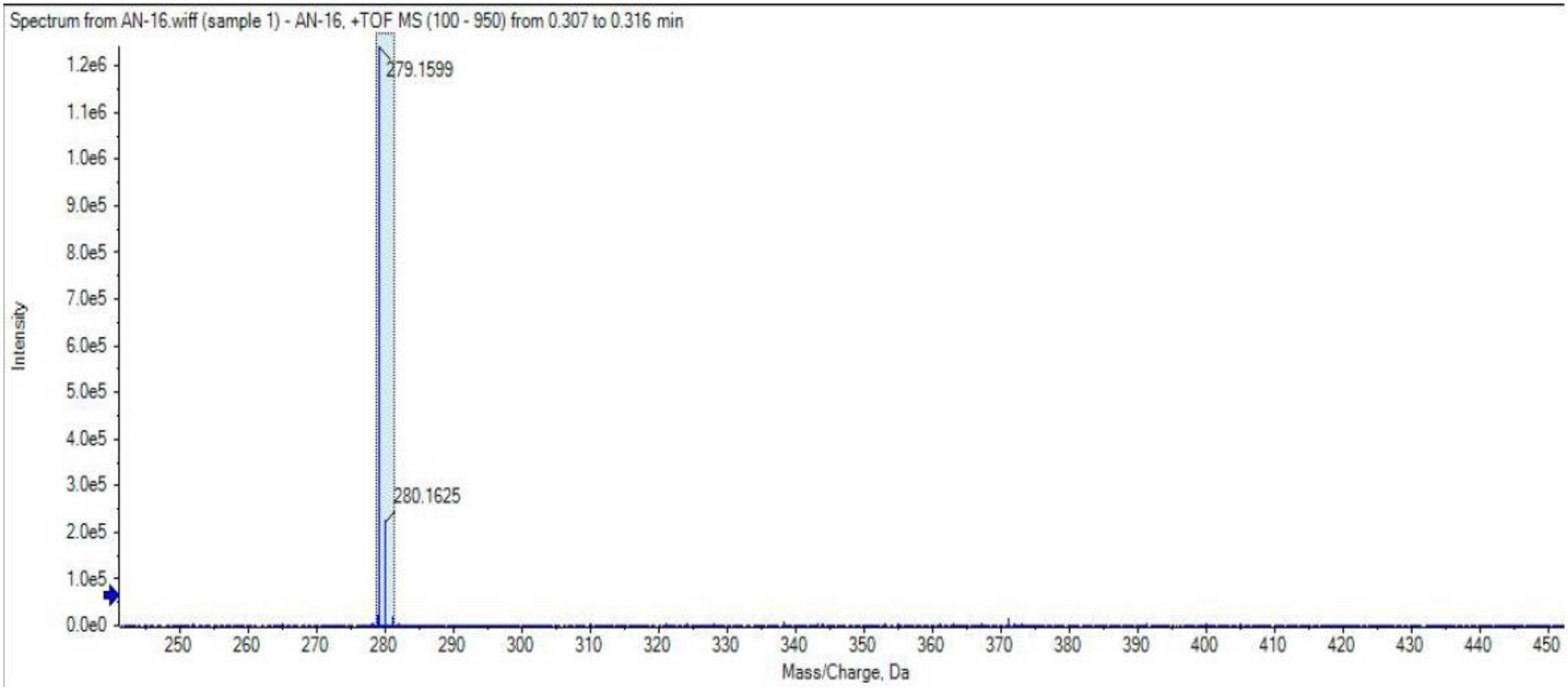
Spectrum from AN-16.wiff (sample 1) - AN-16, +TOF MS (100 - 950) from 0.307 to 0.316 min
279.1599
280.1625
Intensity
1.2e6
1.1e6
1.0e6
9.0e5
8.0e5
7.0e5
6.0e5
5.0e5
4.0e5
3.0e5
2.0e5
1.0e5
0.0e0
250
260
270
280
290
300
310
320
330
340
350
360
370
380
390
400
410
420
430
440
450
Mass/Charge, Da

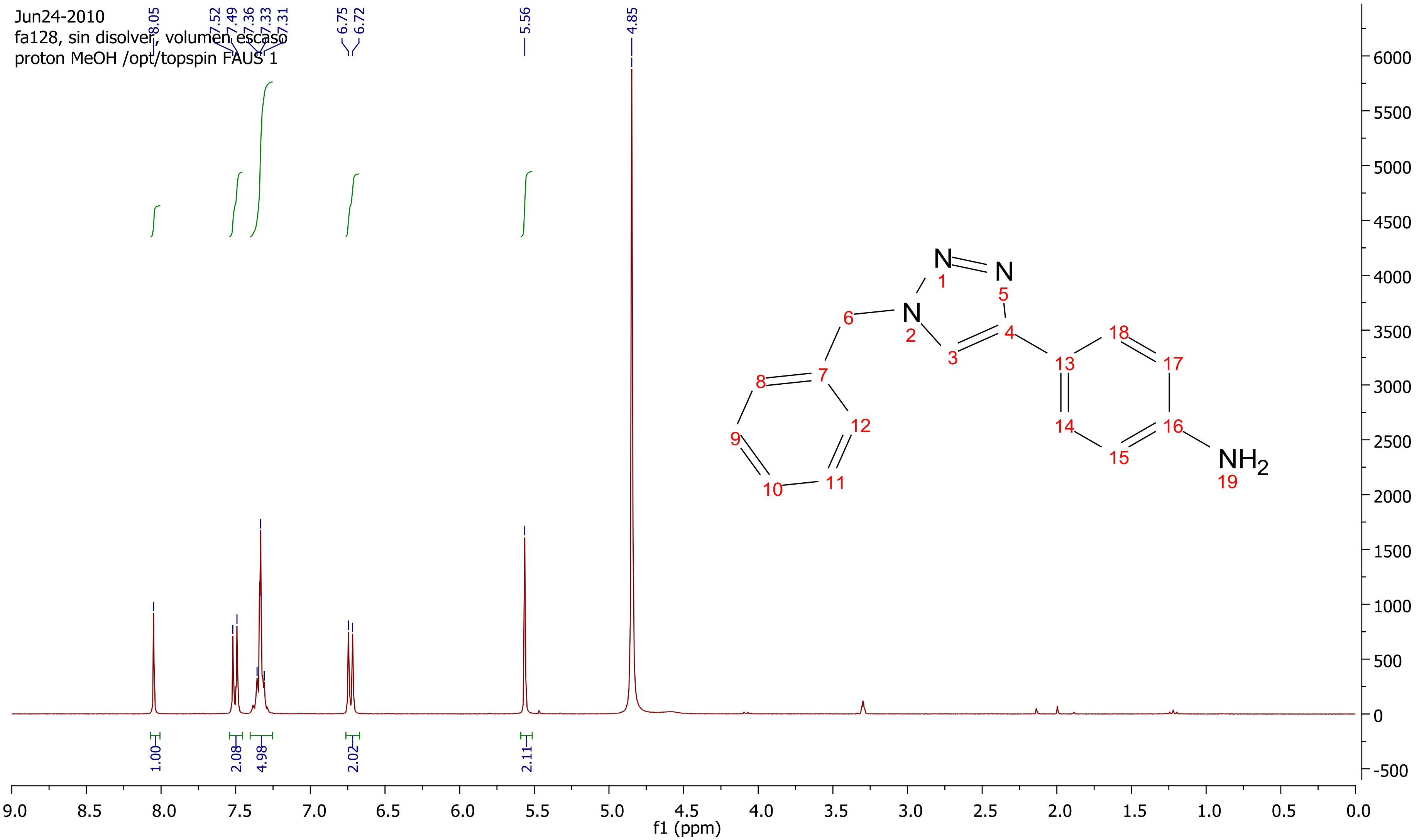
Jun24-2010
fa128, sin disolver, volumen escaso
proton MeOH /opt/topspin FAUS 1
8.05
7.52
7.49
7.36
7.33
7.31
6.75
6.72
5.56
4.85
1.00
2.08
4.98
2.02
2.11
f1 (ppm)
NH2

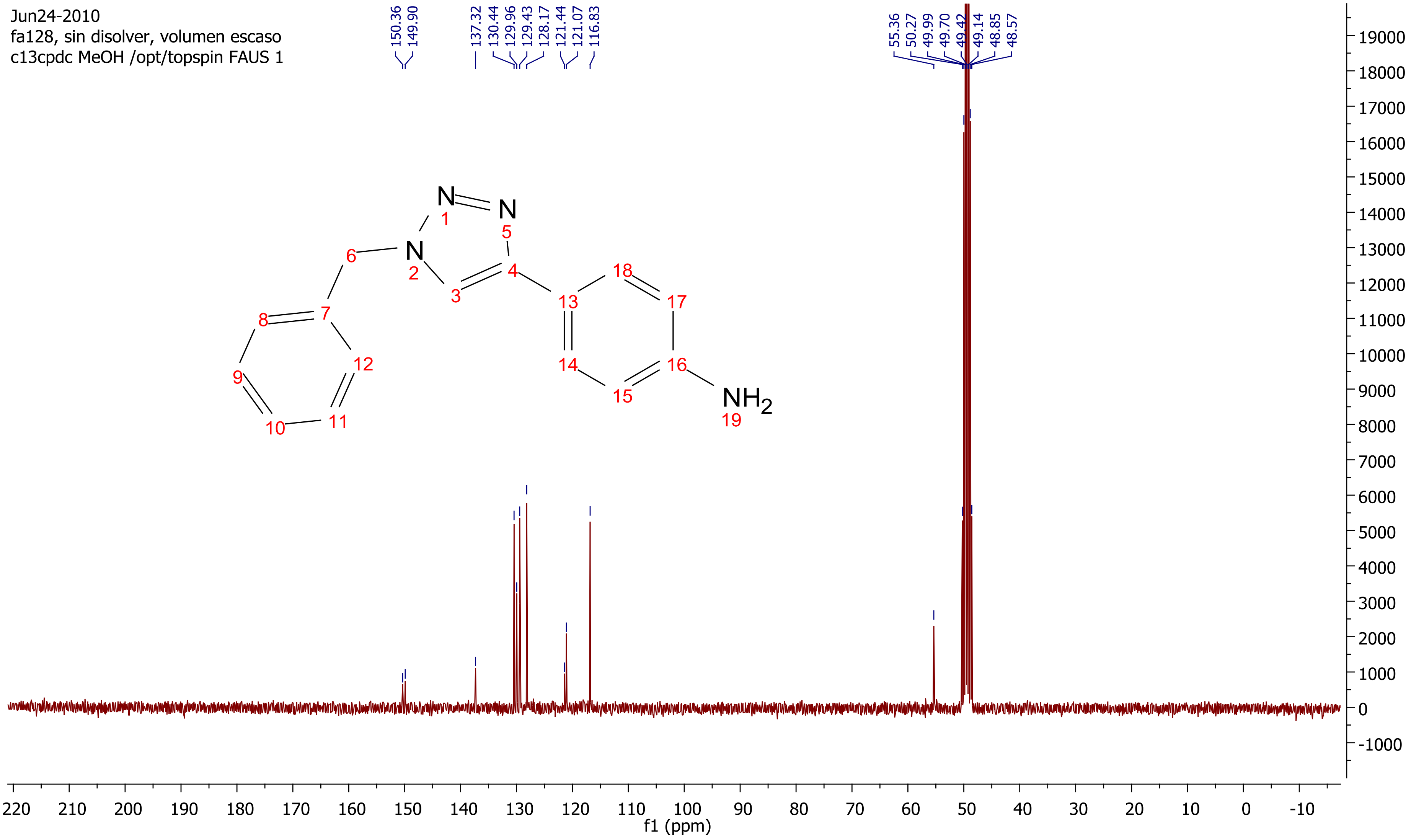
Jun24-2010
fa128, sin disolver, volumen escaso
c13cpdc MeOH /opt/topspin FAUS 1
150.36
149.90
137.32
130.44
129.96
129.43
128.17
121.44
121.07
116.83
55.36
50.27
49.99
49.70
49.42
49.14
48.85
48.57
NH2
f1 (ppm)

AB4

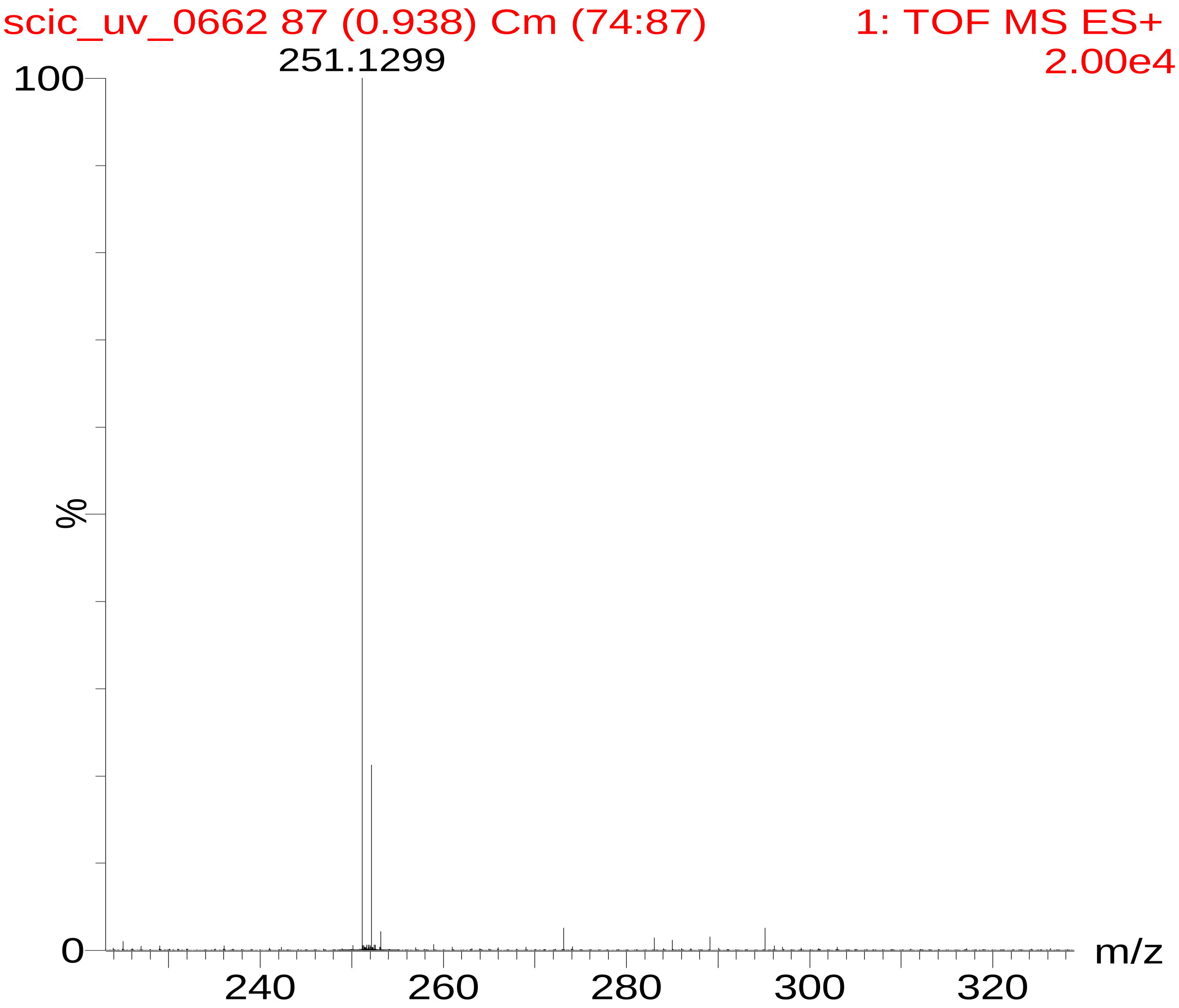
scic_uv_0662 87 (0.938) Cm (74:87)
1: TOF MS ES+
2.00e4
251.1299
100
%
0
240
260
280
300
320
m/z

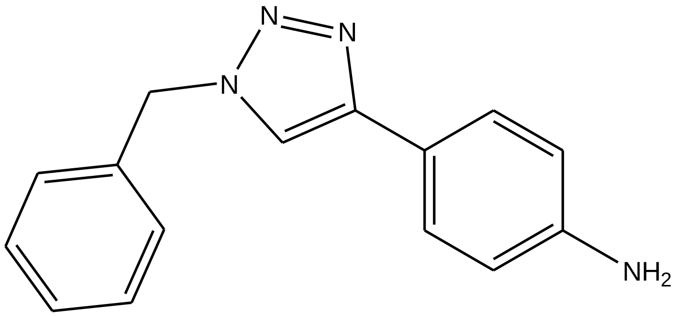
N
N
N
NH2

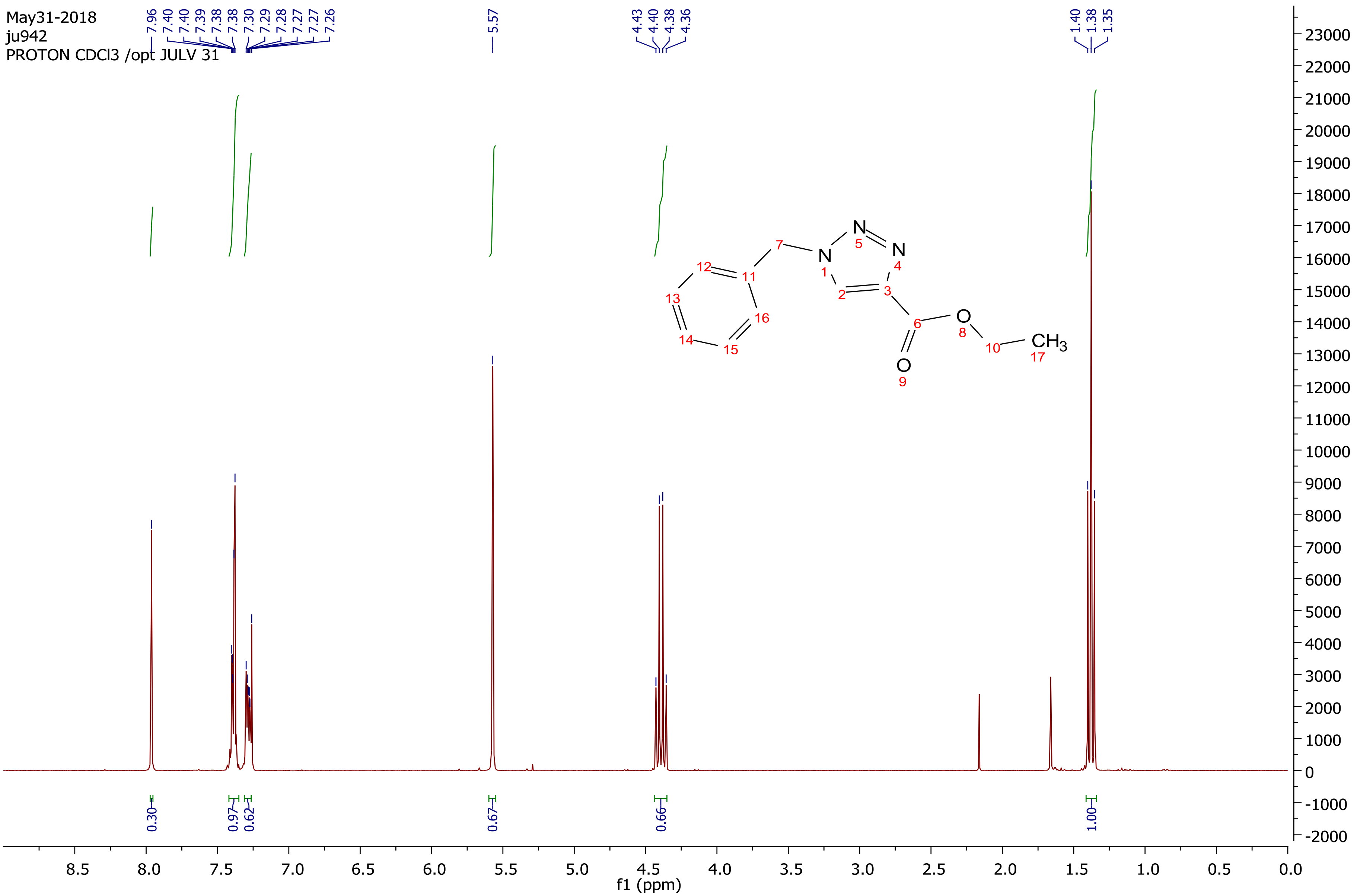
May31-2018
ju942
PROTON CDCl3 /opt JULV 31
7.96 7.40 7.40 7.39 7.38 7.38 7.30 7.29 7.28 7.27 7.27 7.26
5.57
4.43 4.40 4.38 4.36
1.40 1.38 1.35
0.30 0.97 0.62 0.67 0.66 1.00
f1 (ppm)
N
N
N
O
O
CH3
1 2 3 4 5 6 7 8 9 10 11 12 13 14 15 16 17

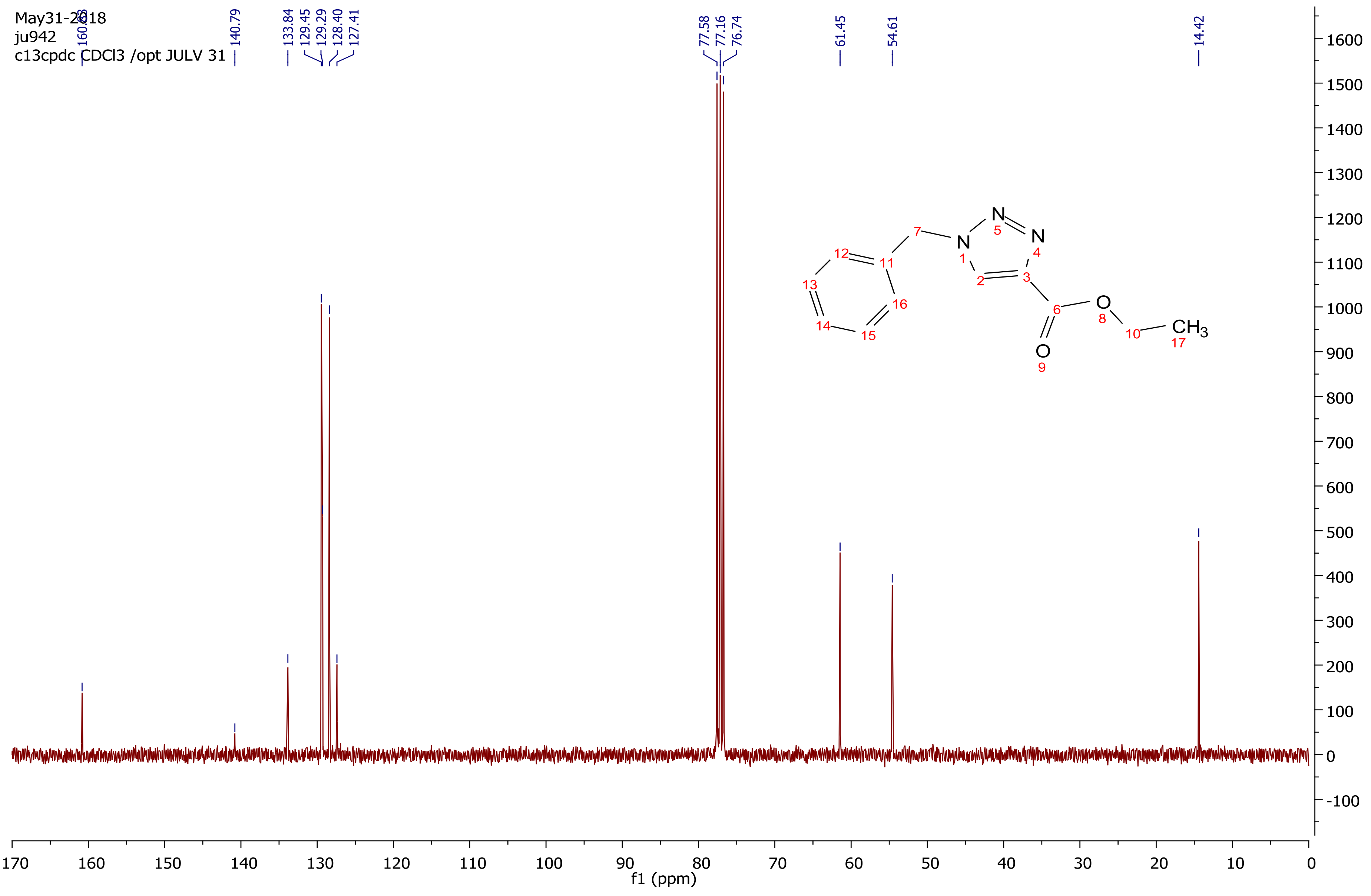
May31-2018
ju942
c13cpdc CDCl3 /opt JULV 31
160.69
140.79
133.84
129.45
129.29
128.40
127.41
77.58
77.16
76.74
61.45
54.61
14.42
f1 (ppm)

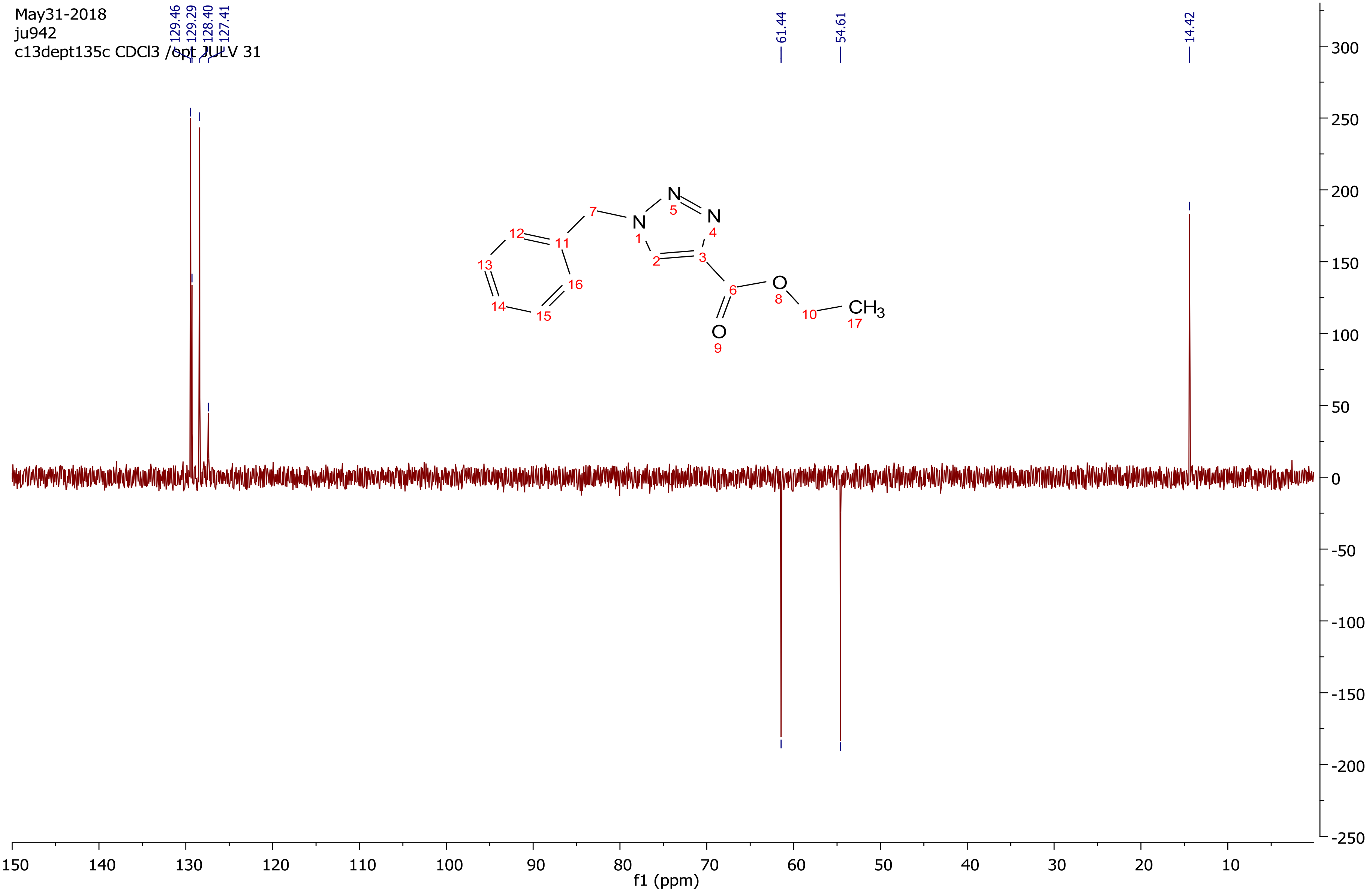
May31-2018
ju942
c13dept135c CDCl3 /opt JULV 31
129.46
129.29
128.40
127.41
61.44
54.61
14.42
N
N
N
1
2
3
4
5
6
7
8
9
10
11
12
13
14
15
16
17
O
O
CH3
300
250
200
150
100
50
0
-50
-100
-150
-200
-250
150
140
130
120
110
100
90
80
70
60
50
40
30
20
10
f1 (ppm)

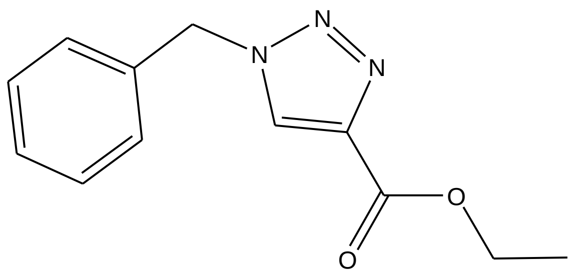
N
N
N
O
O

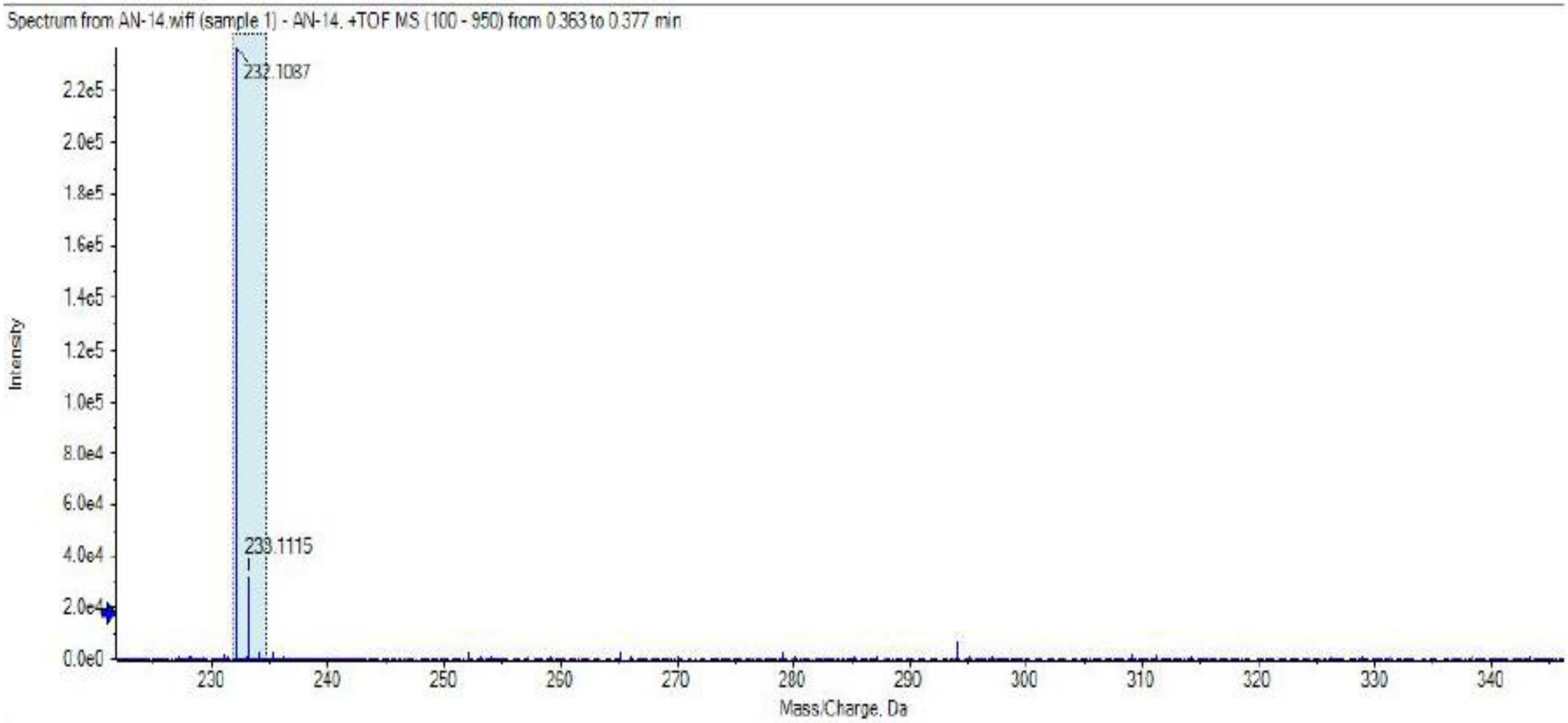
Spectrum from AN-14.wiff (sample 1) - AN-14, +TOF MS (100 - 950) from 0.363 to 0.377 min
232.1087
233.1115
Intensity
2.2e5
2.0e5
1.8e5
1.6e5
1.4e5
1.2e5
1.0e5
8.0e4
6.0e4
4.0e4
2.0e4
0.0e0
230
240
250
260
270
280
290
300
310
320
330
340
Mass/Charge, Da

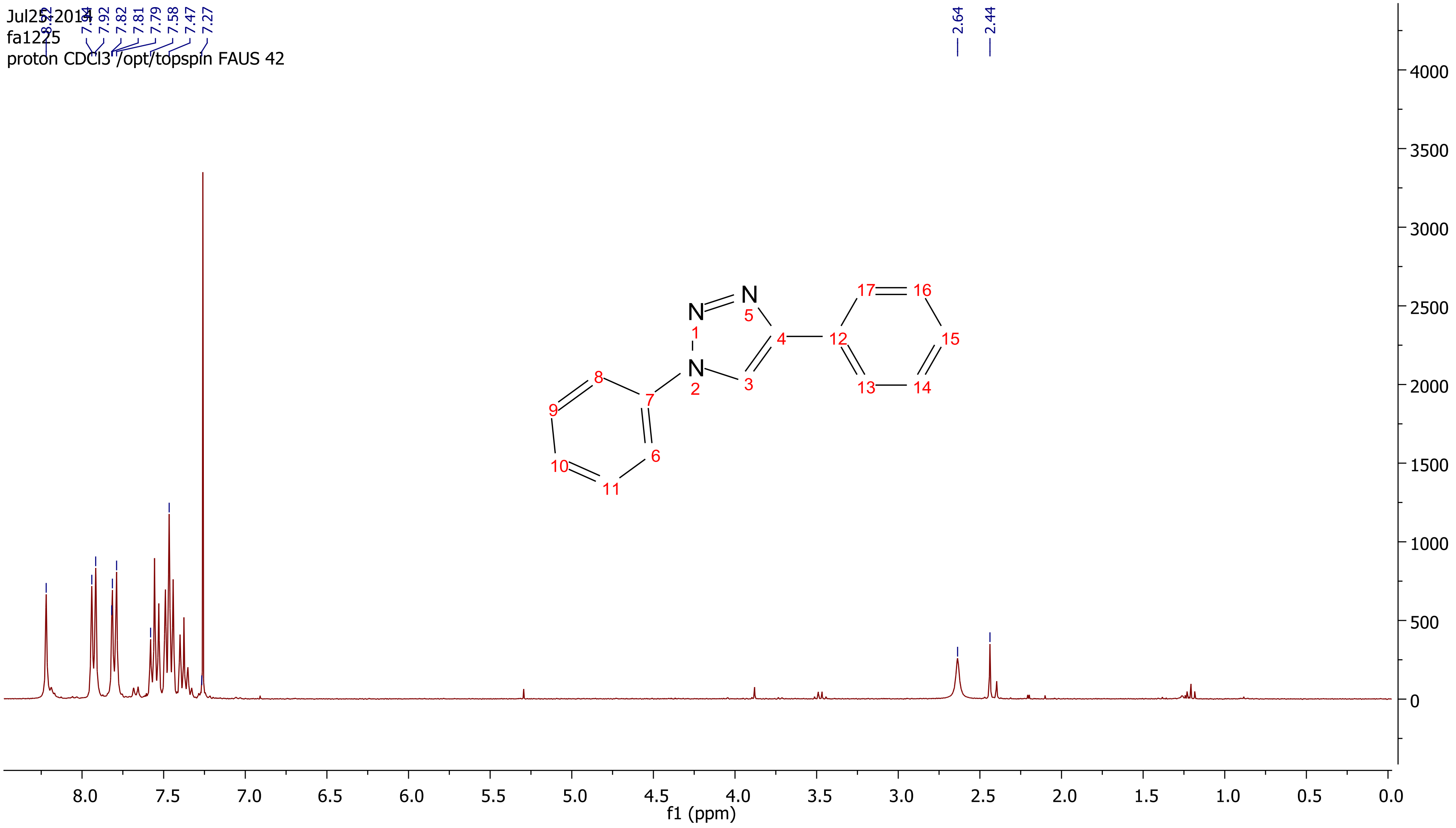
Jul25-2014
fa1225
proton CDCl3 /opt/topspin FAUS 42
8.22
7.94
7.92
7.82
7.81
7.79
7.58
7.47
7.27
2.64
2.44
N
N
N
1
2
3
4
5
6
7
8
9
10
11
12
13
14
15
16
17
f1 (ppm)

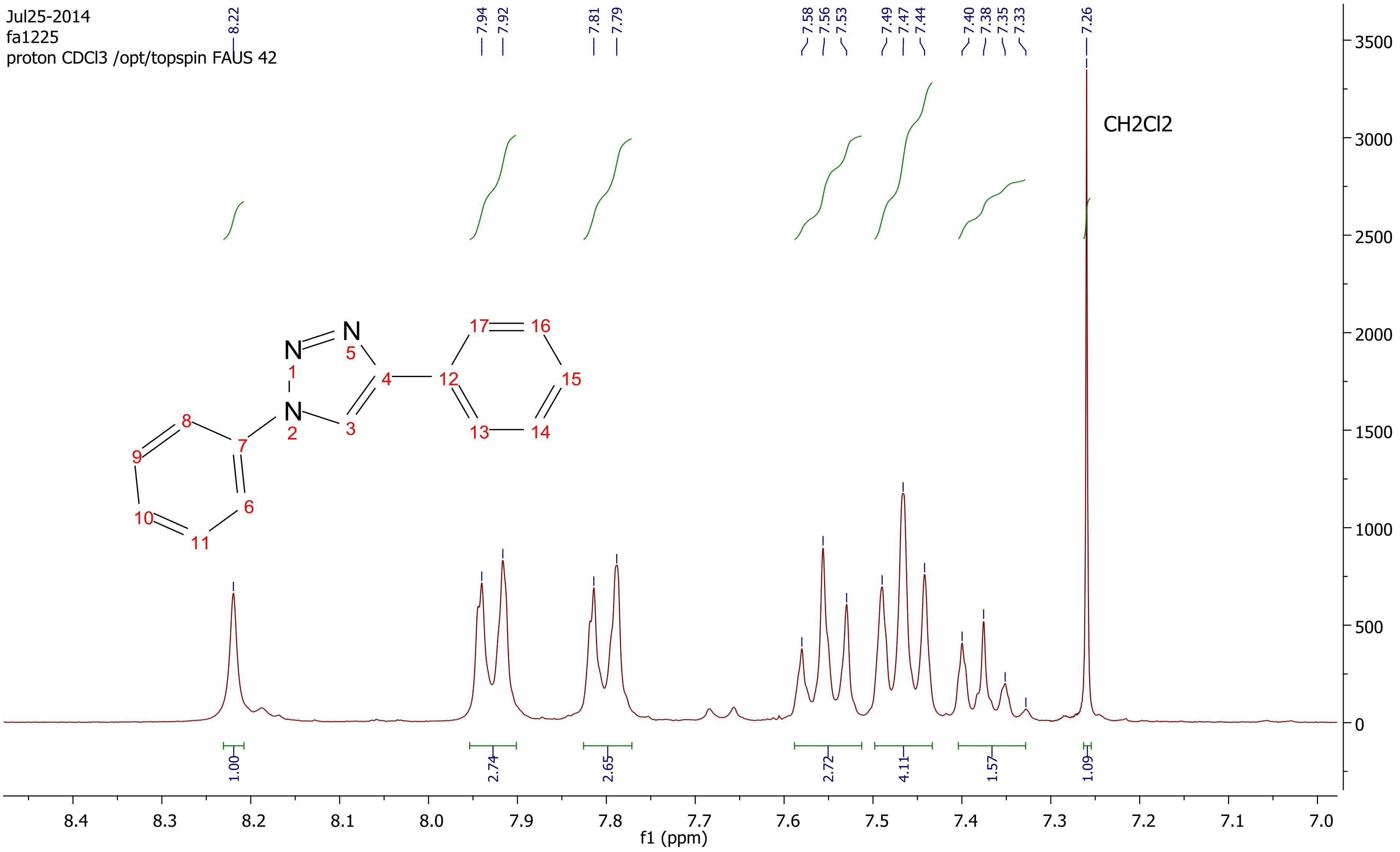
Jul25-2014
fa1225
proton CDCl3 /opt/topspin FAUS 42
8.22
7.94
7.92
7.81
7.79
7.58
7.56
7.53
7.49
7.47
7.44
7.40
7.38
7.35
7.33
7.26
CH2Cl2
1.00
2.74
2.65
2.72
4.11
1.57
1.09
f1 (ppm)

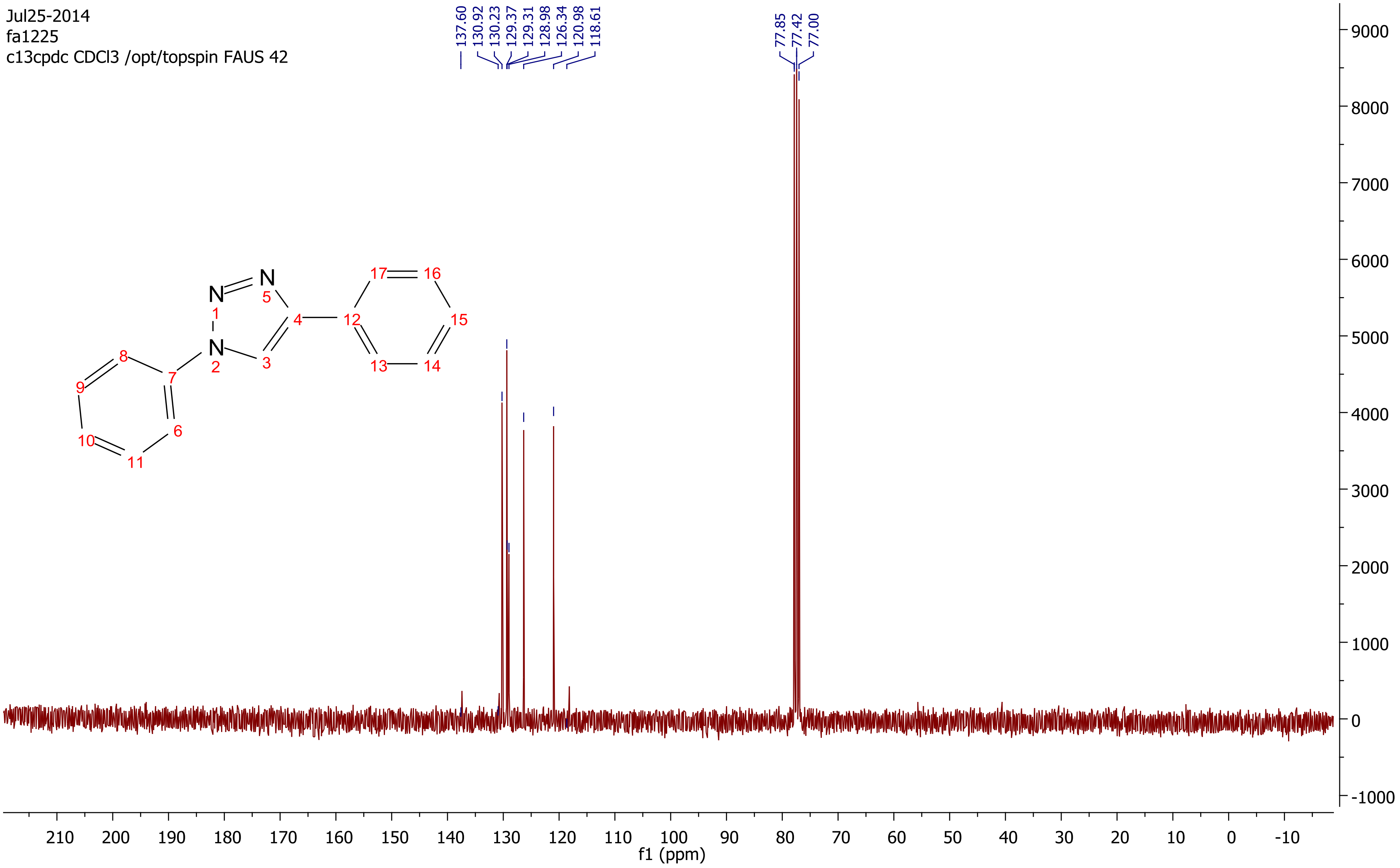

Jul25-2014
fa1225
c13cpdc CDCl3 /opt/topspin FAUS 42
137.60
130.92
130.23
129.37
129.31
128.98
126.34
120.98
118.61
77.85
77.42
77.00
N
N
N
1
2
3
4
5
6
7
8
9
10
11
12
13
14
15
16
17
9000
8000
7000
6000
5000
4000
3000
2000
1000
0
-1000
210
200
190
180
170
160
150
140
130
120
110
100
90
80
70
60
50
40
30
20
10
0
-10
f1 (ppm)

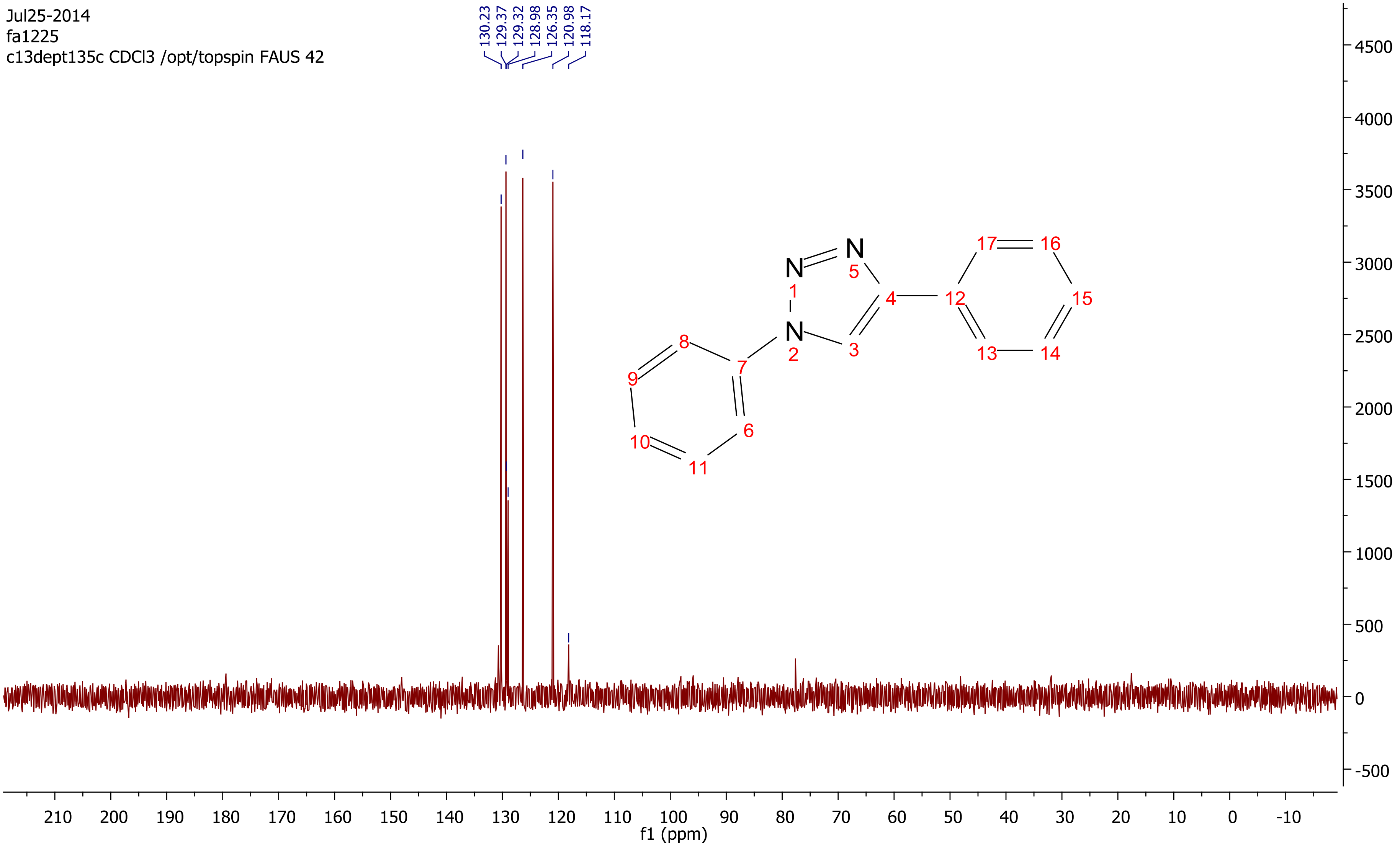

Jul25-2014
fa1225
c13dept135c CDCl3 /opt/topspin FAUS 42
130.23
129.37
129.32
128.98
126.35
120.98
118.17
N
N
N
1
2
3
4
5
6
7
8
9
10
11
12
13
14
15
16
17
4500
4000
3500
3000
2500
2000
1500
1000
500
0
-500
210
200
190
180
170
160
150
140
130
120
110
100
90
80
70
60
50
40
30
20
10
0
-10
f1 (ppm)

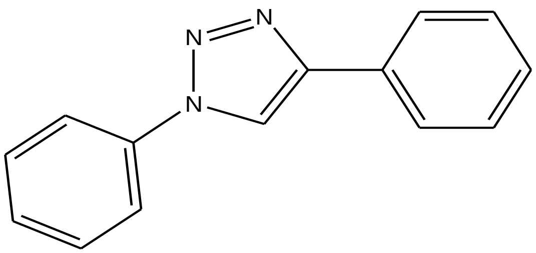

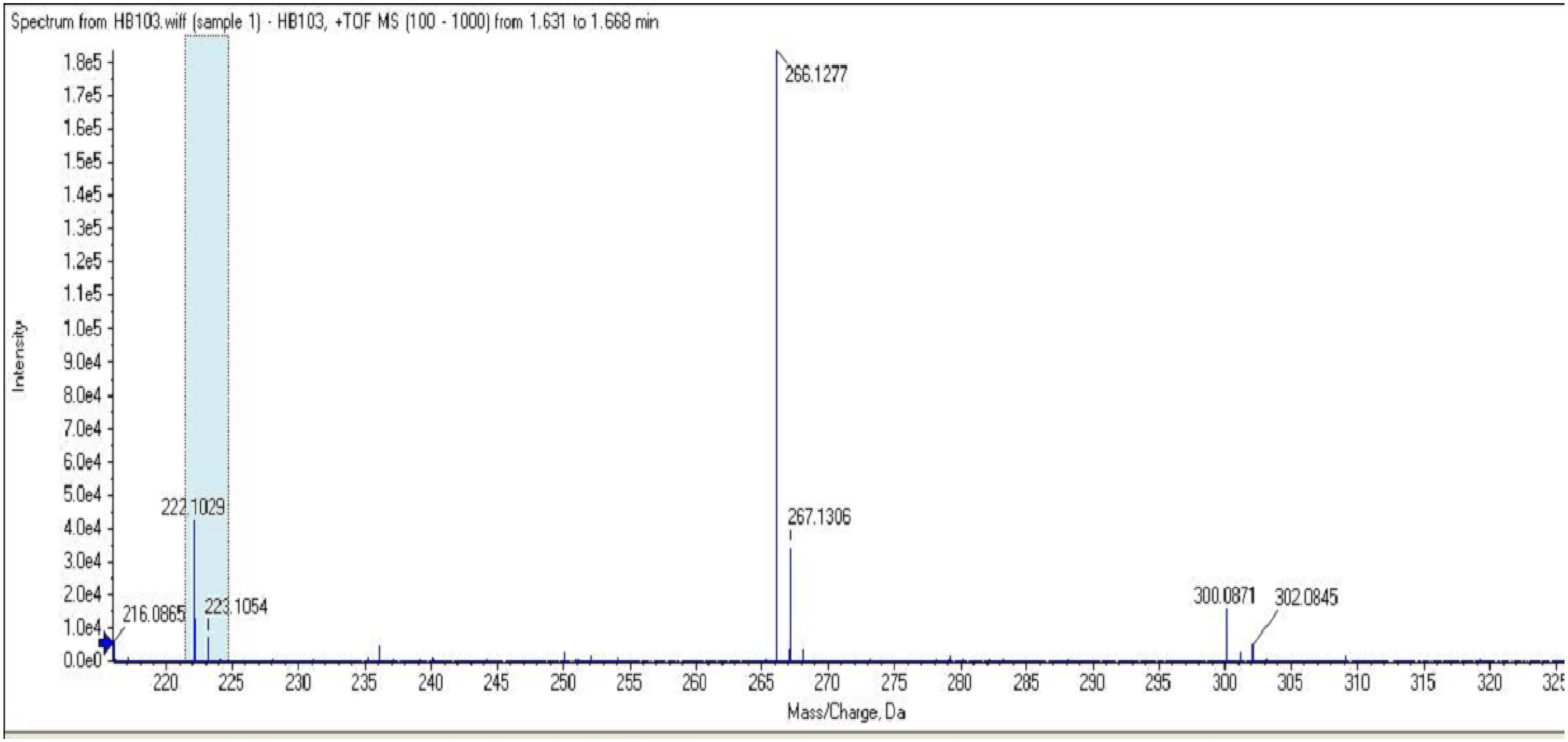
Spectrum from HB103.wiff (sample 1) - HB103, +TOF MS (100 - 1000) from 1.631 to 1.668 min
Intensity
Mass/Charge, Da
266.1277
267.1306
222.1029
223.1054
216.0865
300.0871
302.0845

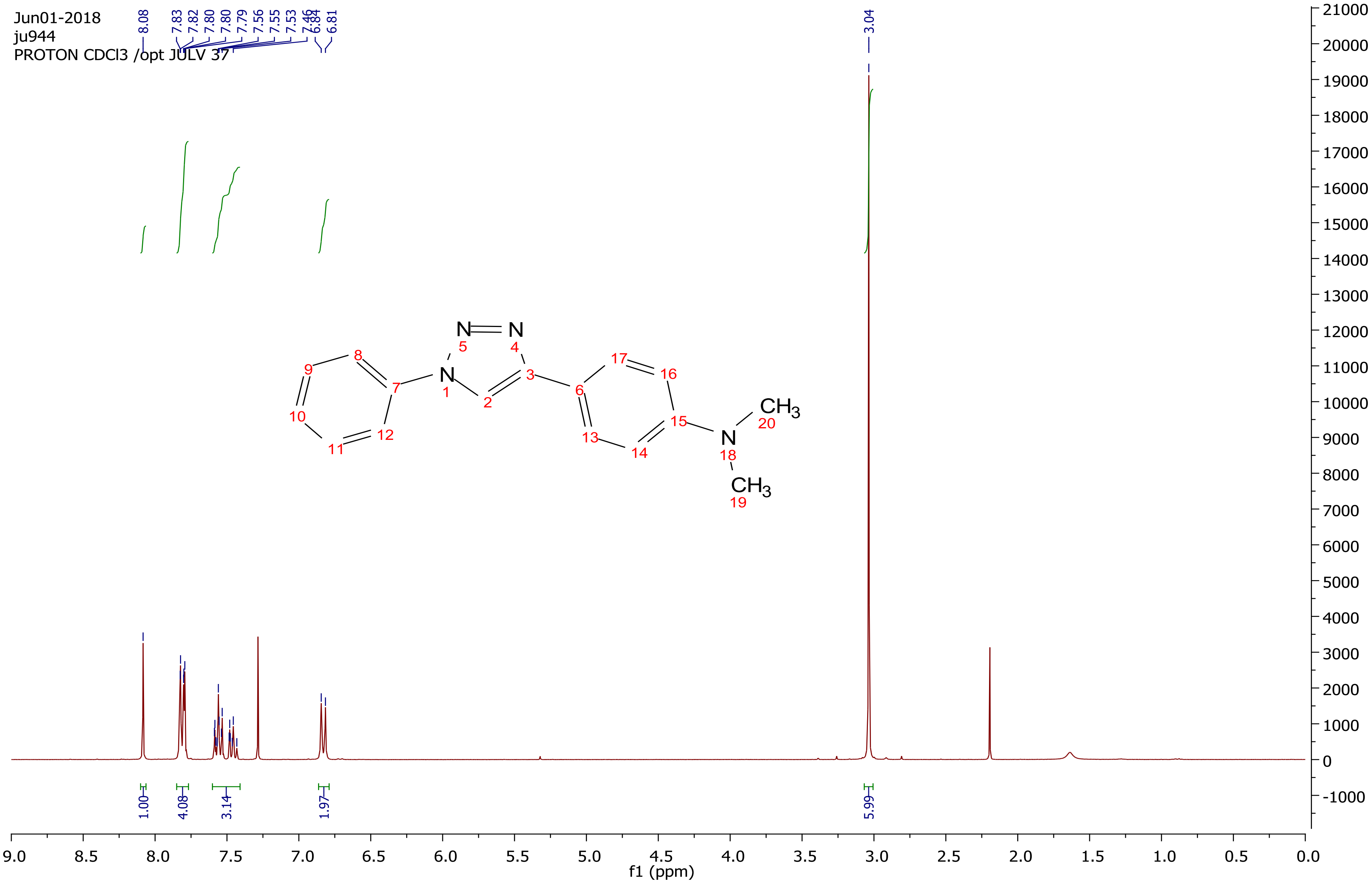
Jun01-2018
ju944
PROTON CDCl3 /opt JULV 37
8.08
7.83
7.82
7.80
7.80
7.79
7.56
7.55
7.53
7.46
6.84
6.81
3.04
1.00
4.08
3.14
1.97
5.99
f1 (ppm)

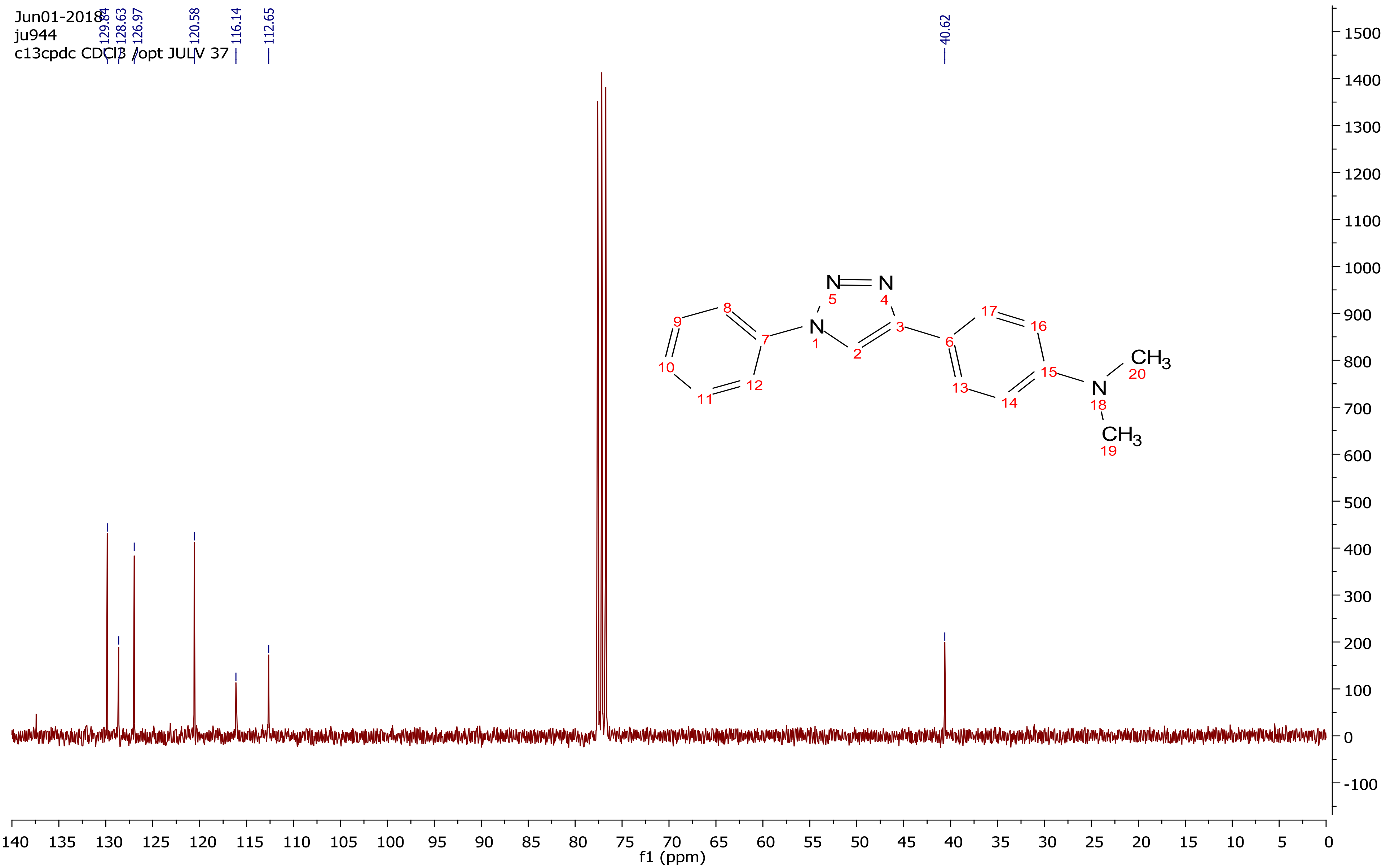
Jun01-2018
ju944
c13cpdc CDCl3 /opt JULV 37
129.84
128.63
126.97
120.58
116.14
112.65
40.62
f1 (ppm)
N
N
N
CH3
CH3

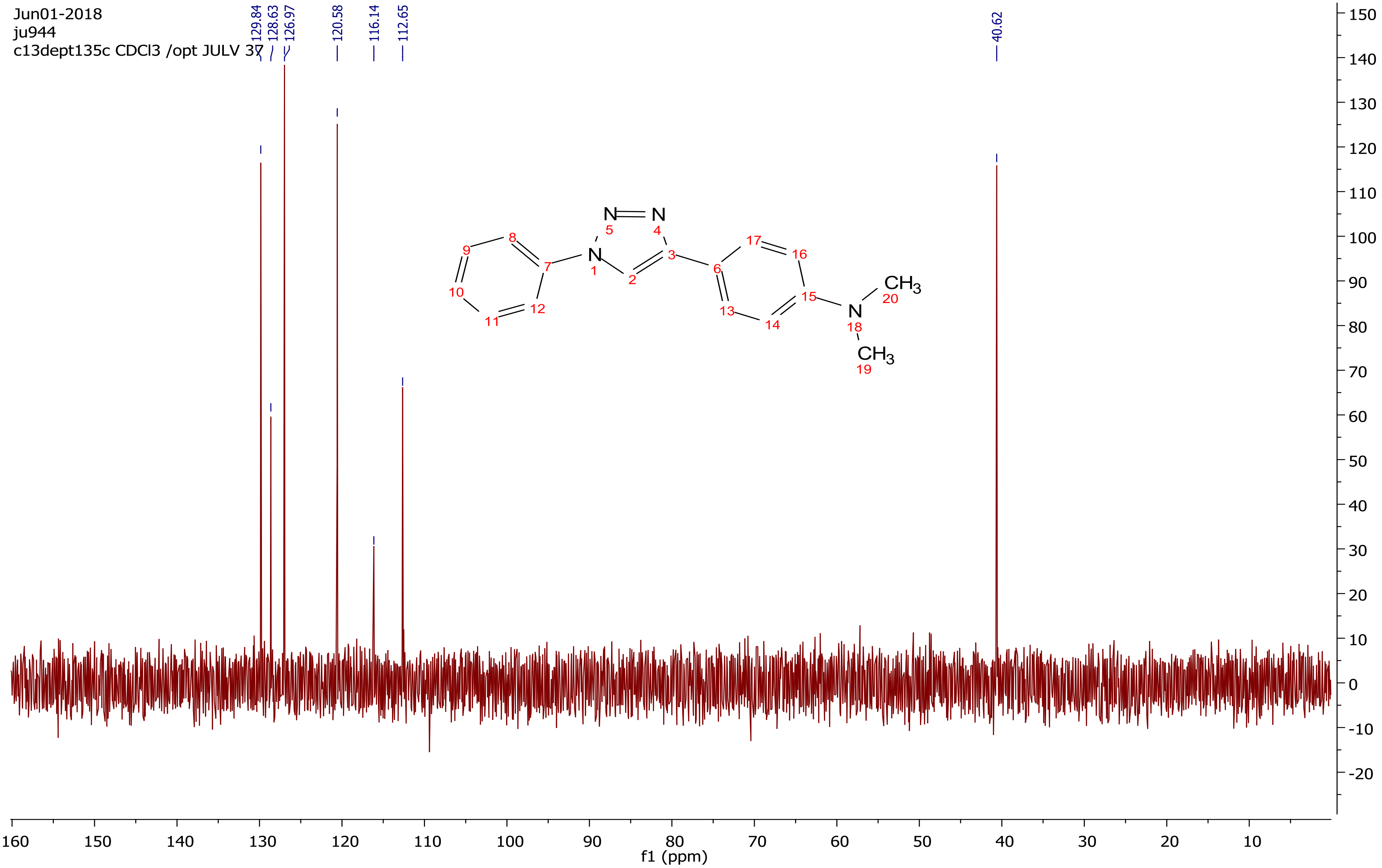

Jun01-2018
ju944
c13dept135c CDCl3 /opt JULV 37
129.84
128.63
126.97
120.58
116.14
112.65
40.62
N
N
CH3
CH3
f1 (ppm)

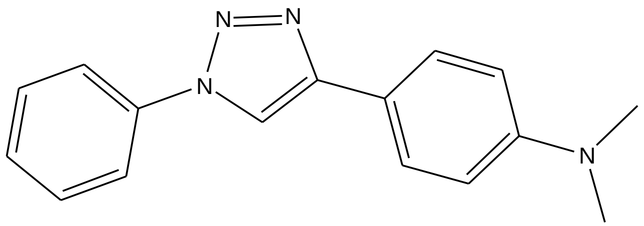

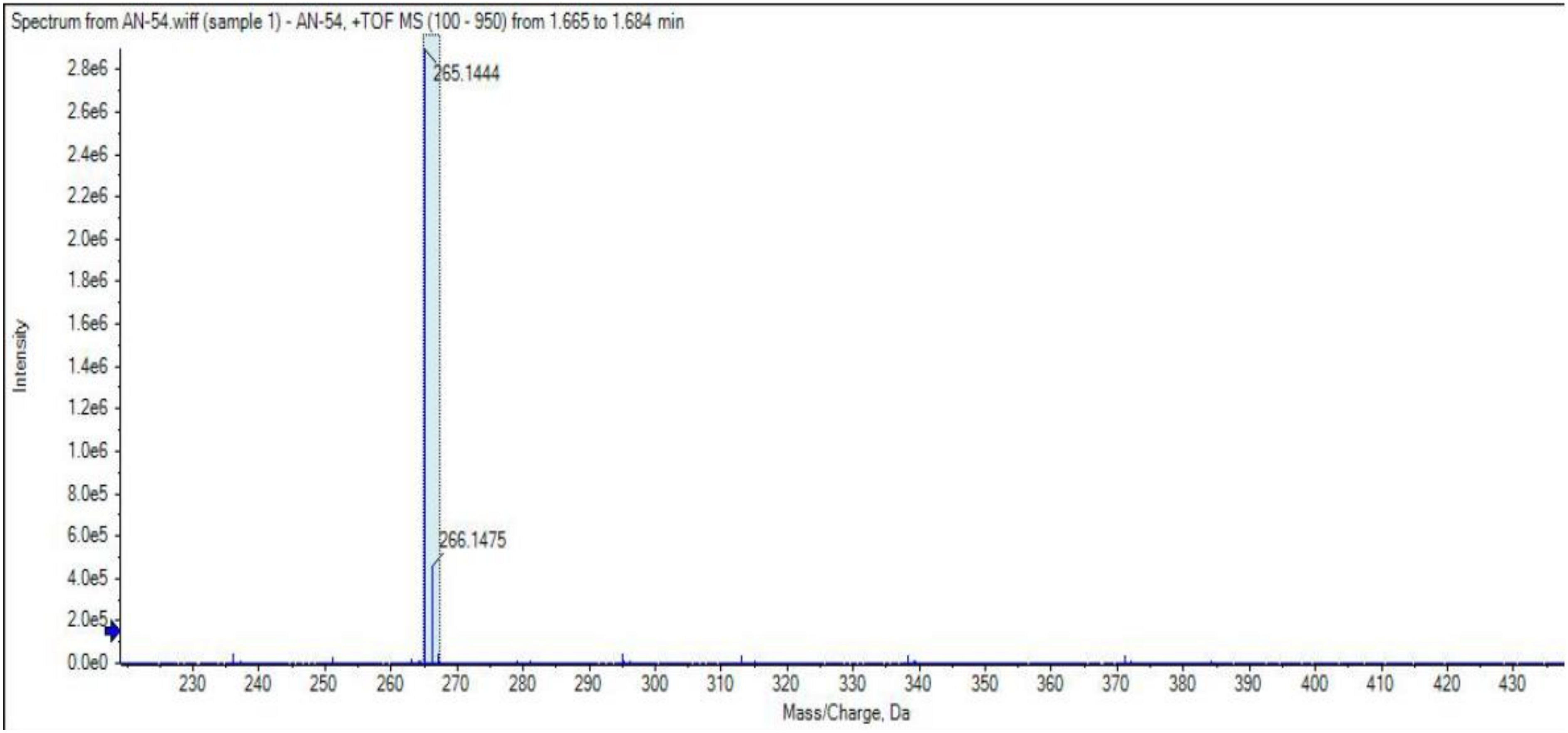
Spectrum from AN-54.wiff (sample 1) - AN-54, +TOF MS (100 - 950) from 1.665 to 1.684 min
265.1444
266.1475
Intensity
Mass/Charge, Da

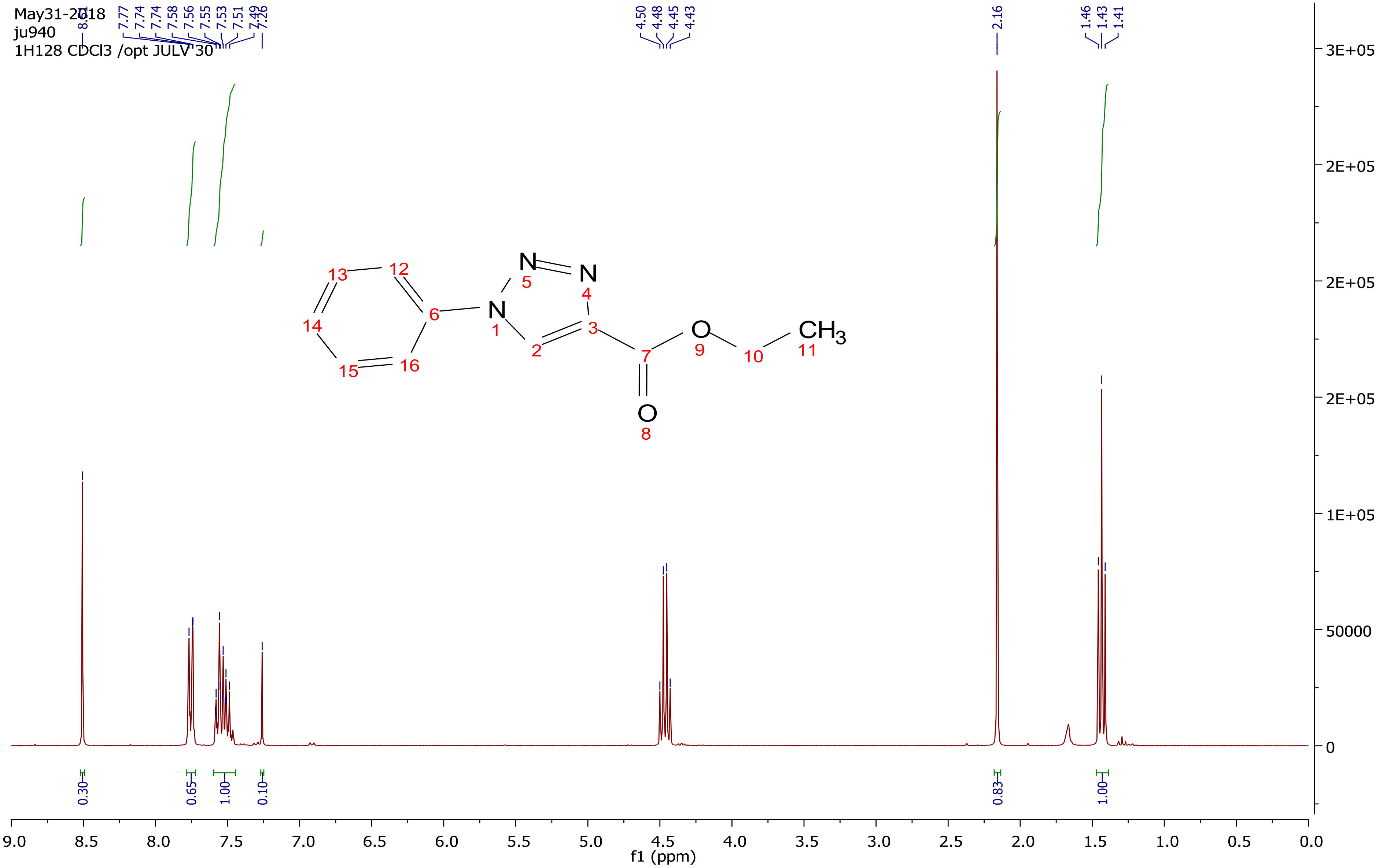

May31-2018
ju940
1H128 CDCl3 /opt JULV 30
8.51
7.77
7.74
7.74
7.58
7.56
7.55
7.53
7.51
7.49
7.26
4.50
4.48
4.45
4.43
2.16
1.46
1.43
1.41
N
N
N
O
O
CH3
0.30
0.65
1.00
0.10
0.83
1.00
f1 (ppm)

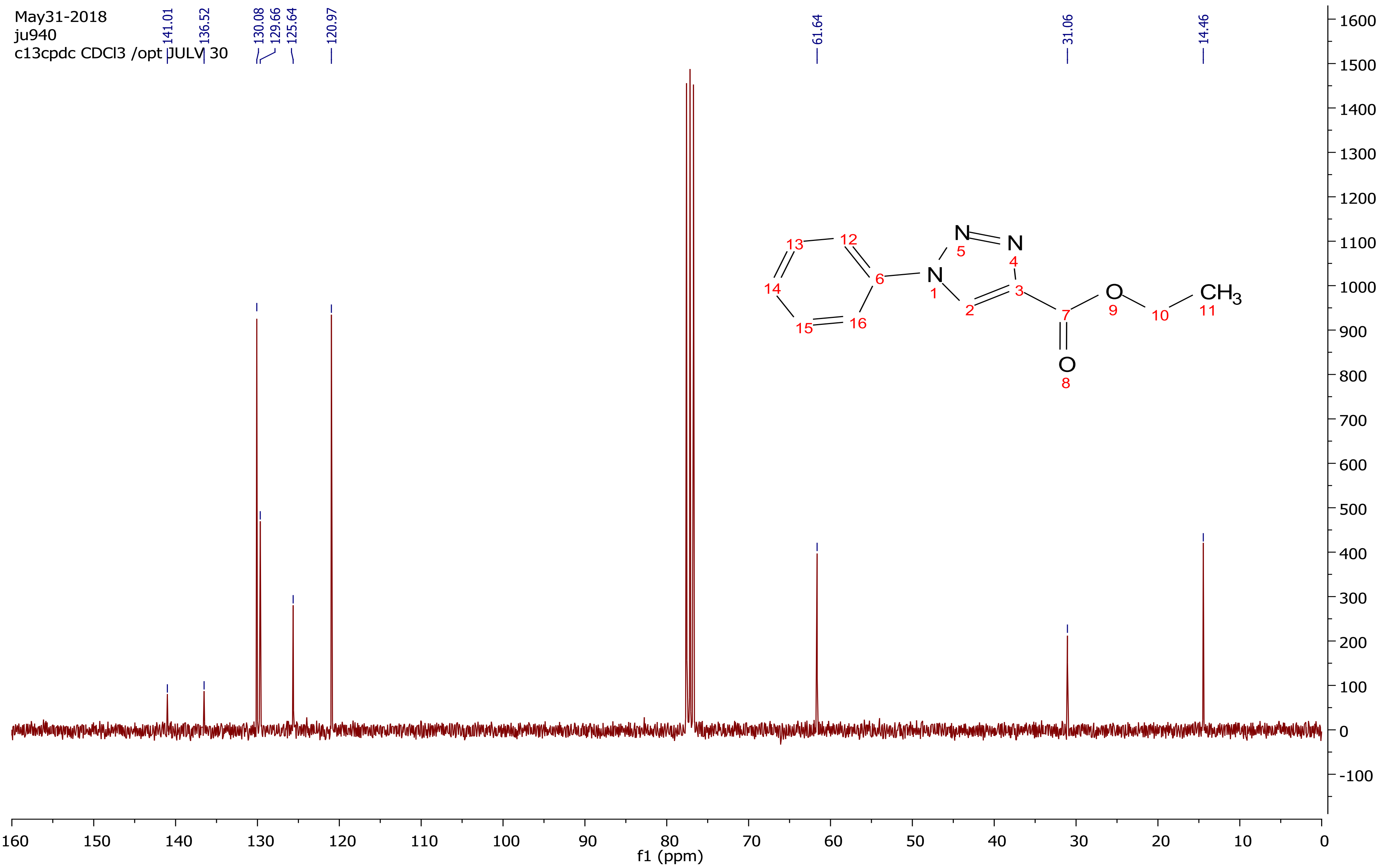
May31-2018
ju940
c13cpdc CDCl3 /opt JULV 30
141.01
136.52
130.08
129.66
125.64
120.97
61.64
31.06
14.46
N
N
N
O
O
CH3
f1 (ppm)

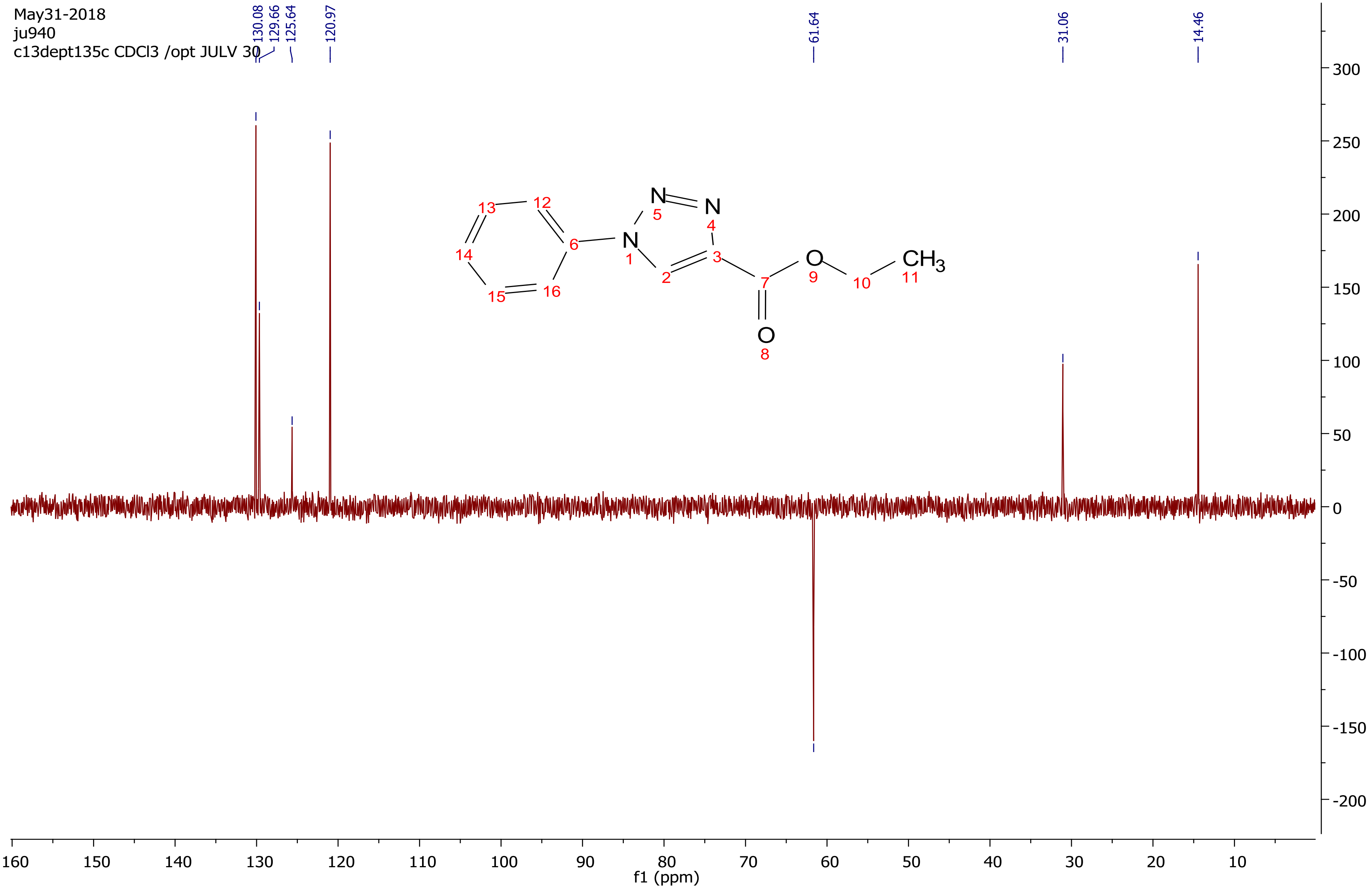
May31-2018
ju940
c13dept135c CDCl3 /opt JULV 30
130.08
129.66
125.64
120.97
61.64
31.06
14.46
N
N
5
4
N
1
3
2
6
12
13
14
15
16
7
O
9
10
CH3
11
O
8
300
250
200
150
100
50
0
-50
-100
-150
-200
160
150
140
130
120
110
100
90
80
70
60
50
40
30
20
10
f1 (ppm)

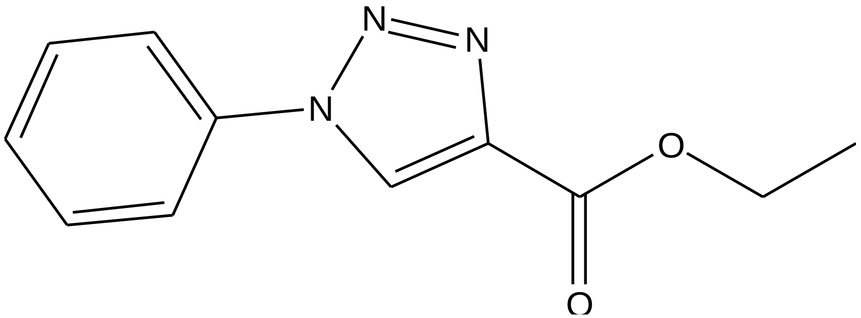

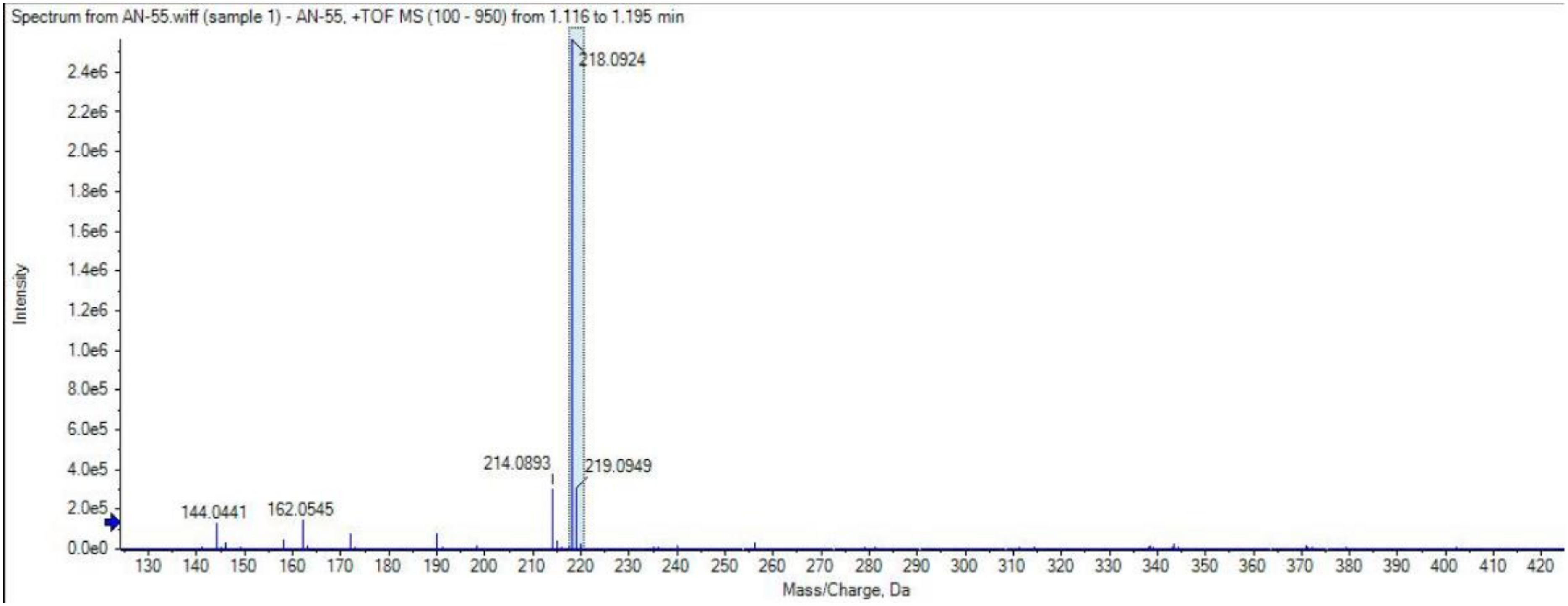
Spectrum from AN-55.wiff (sample 1) - AN-55, +TOF MS (100 - 950) from 1.116 to 1.195 min
218.0924
214.0893
219.0949
144.0441
162.0545
Intensity
Mass/Charge, Da

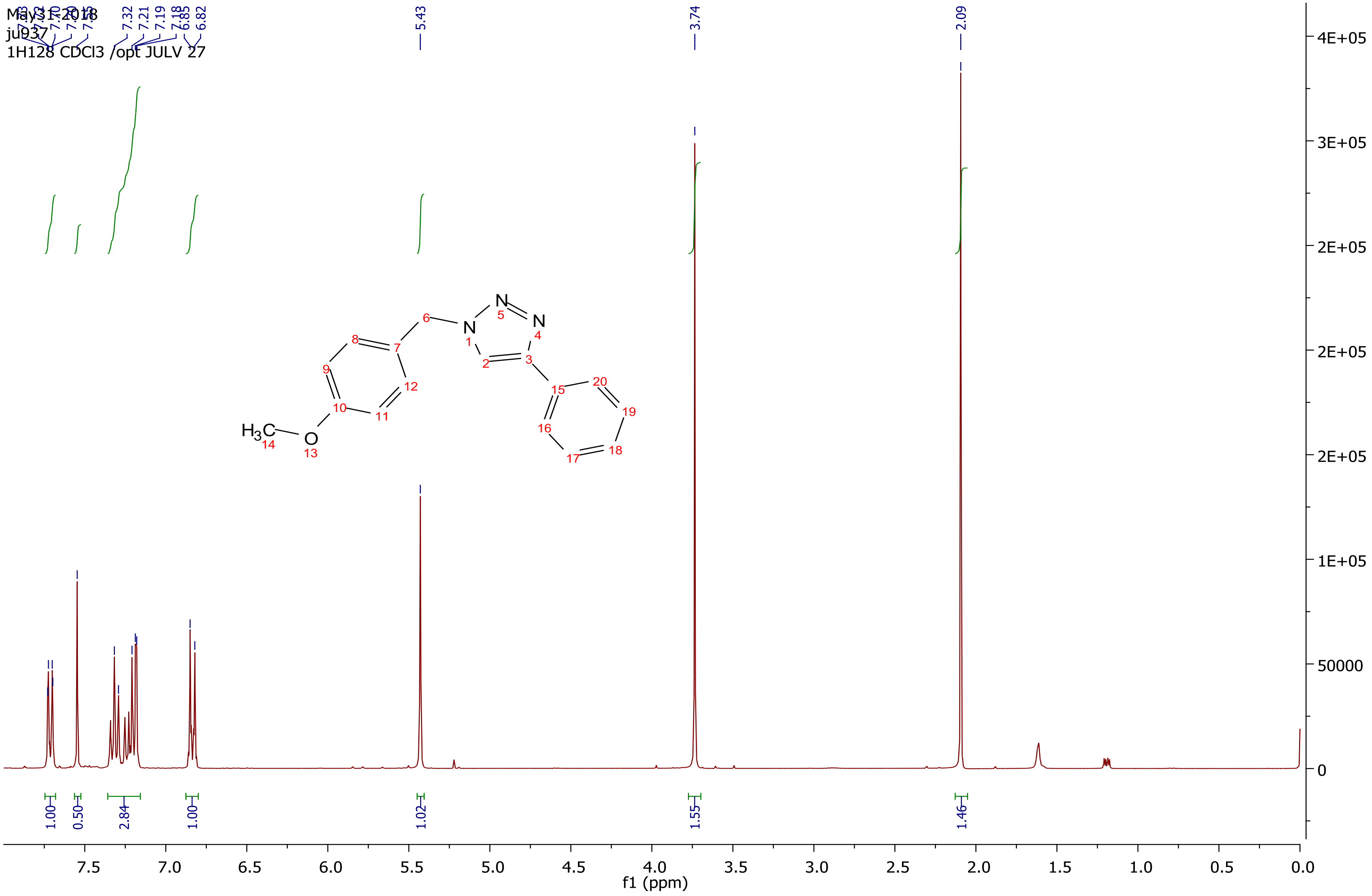
May31-2018
ju937
1H128 CDCl3 /opt JULV 27
7.32
7.21
7.19
7.18
6.85
6.82
5.43
3.74
2.09
1.00
0.50
2.84
1.00
1.02
1.55
1.46
f1 (ppm)
4E+05
3E+05
2E+05
1E+05
50000
0

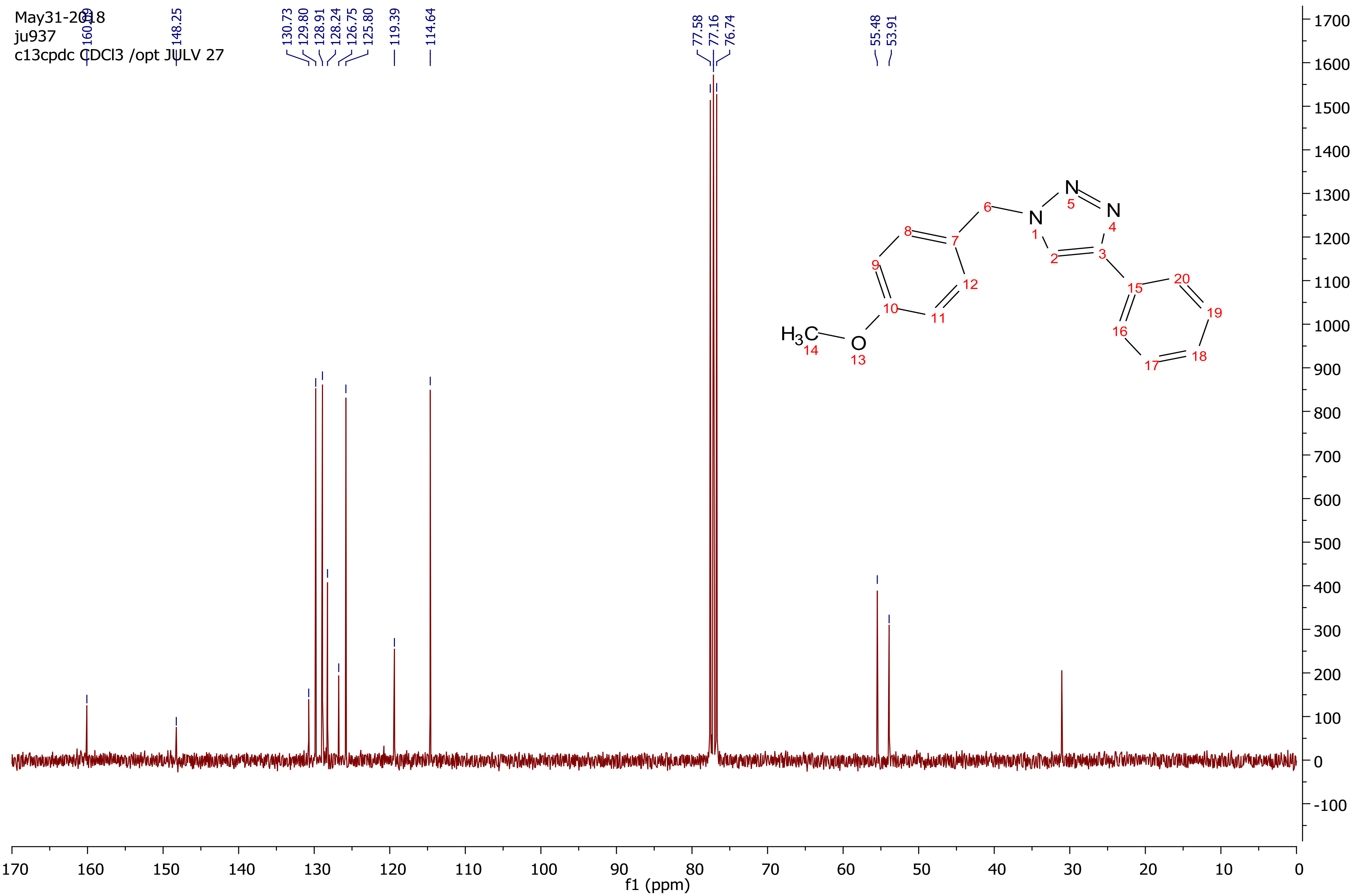

May31-2018
ju937
c13cpdc CDCl3 /opt JULV 27
160.19
148.25
130.73
129.80
128.91
128.24
126.75
125.80
119.39
114.64
77.58
77.16
76.74
55.48
53.91
f1 (ppm)

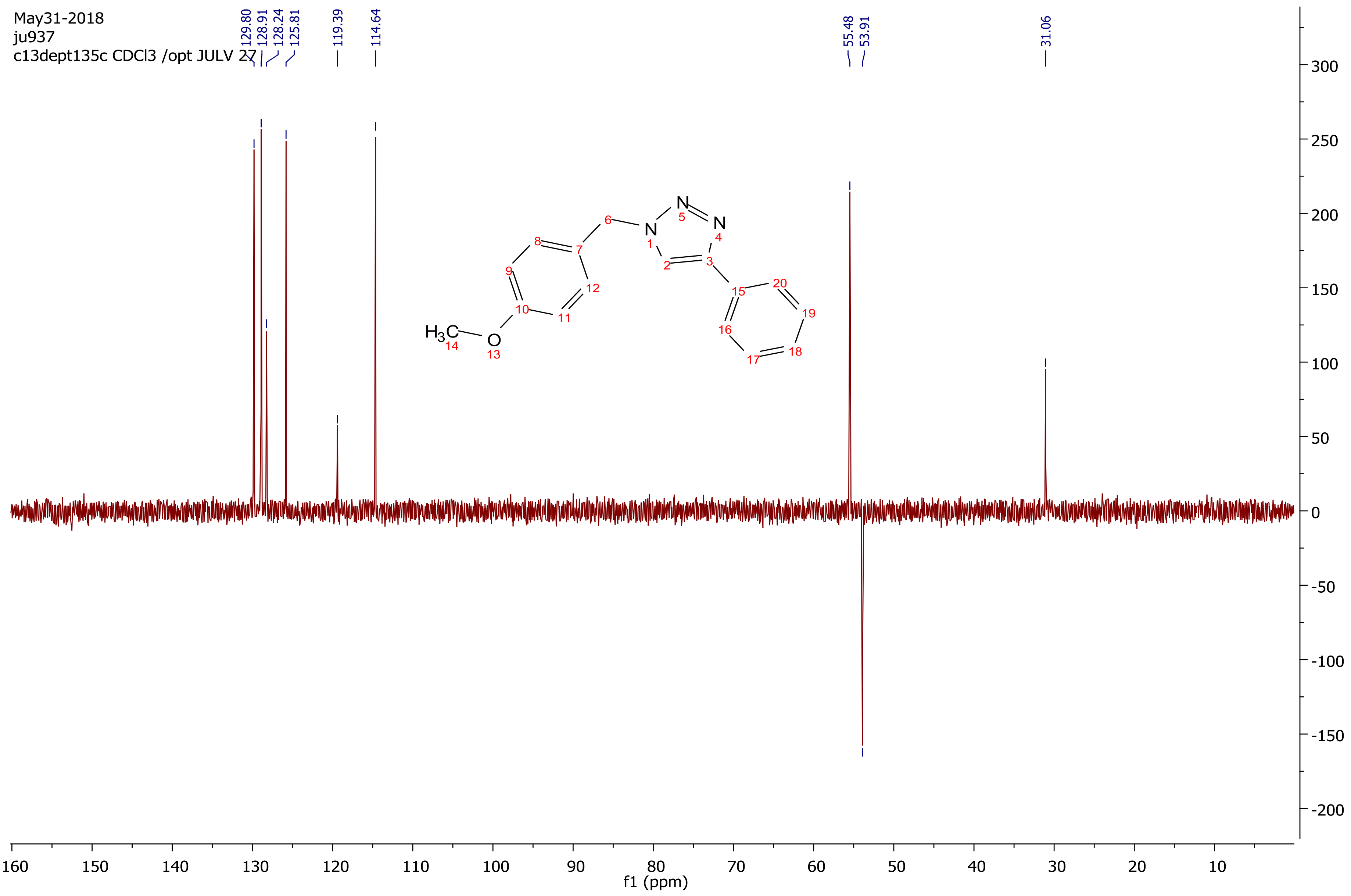

May31-2018
ju937
c13dept135c CDCl3 /opt JULV 27
129.80
128.91
128.24
125.81
119.39
114.64
55.48
53.91
31.06
f1 (ppm)

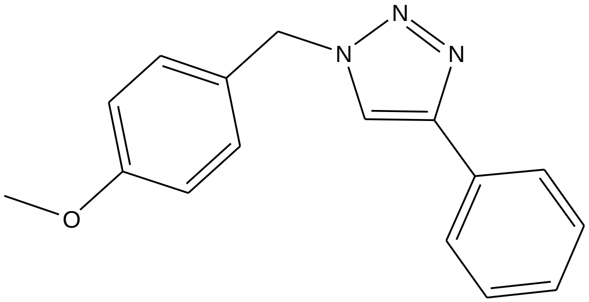
N
N
N
O

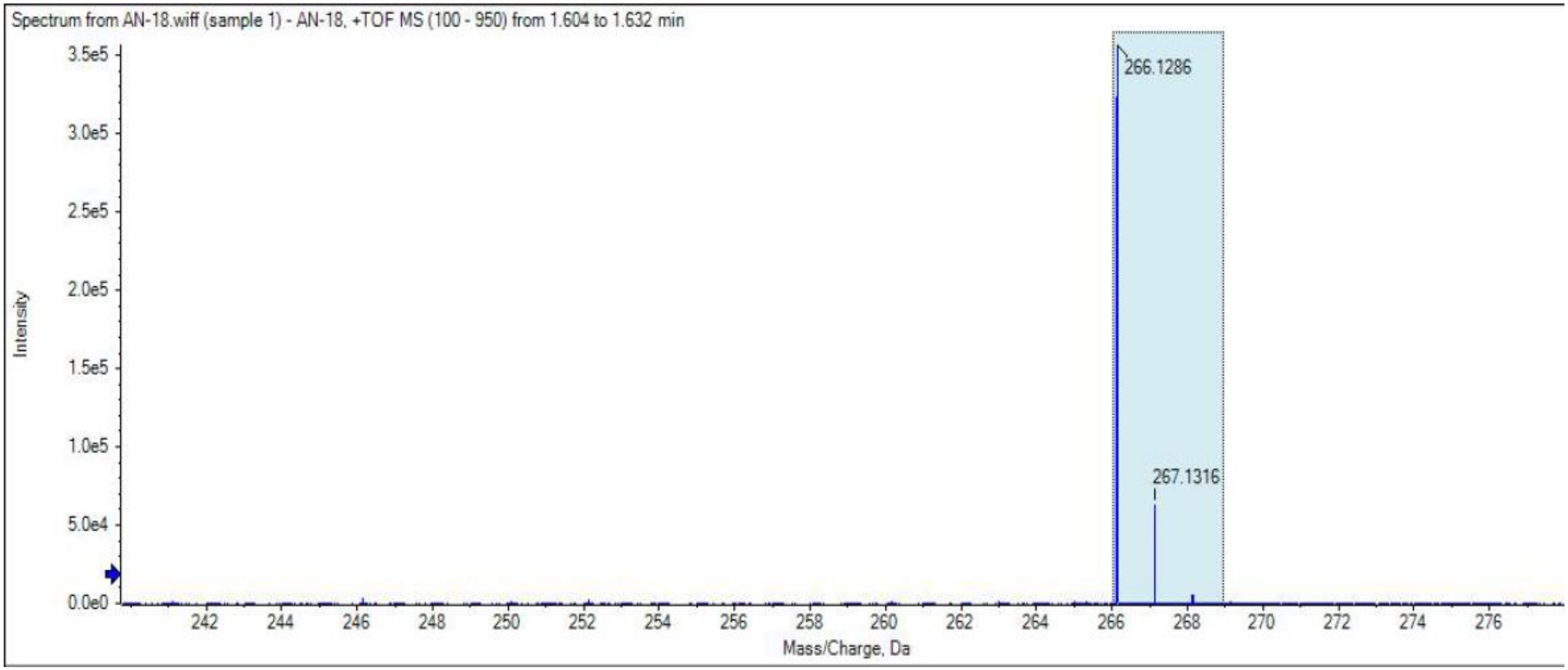
Spectrum from AN-18.wiff (sample 1) - AN-18, +TOF MS (100 - 950) from 1.604 to 1.632 min
266.1286
267.1316
Intensity
Mass/Charge, Da
3.5e5
3.0e5
2.5e5
2.0e5
1.5e5
1.0e5
5.0e4
0.0e0
242
244
246
248
250
252
254
256
258
260
262
264
266
268
270
272
274
276

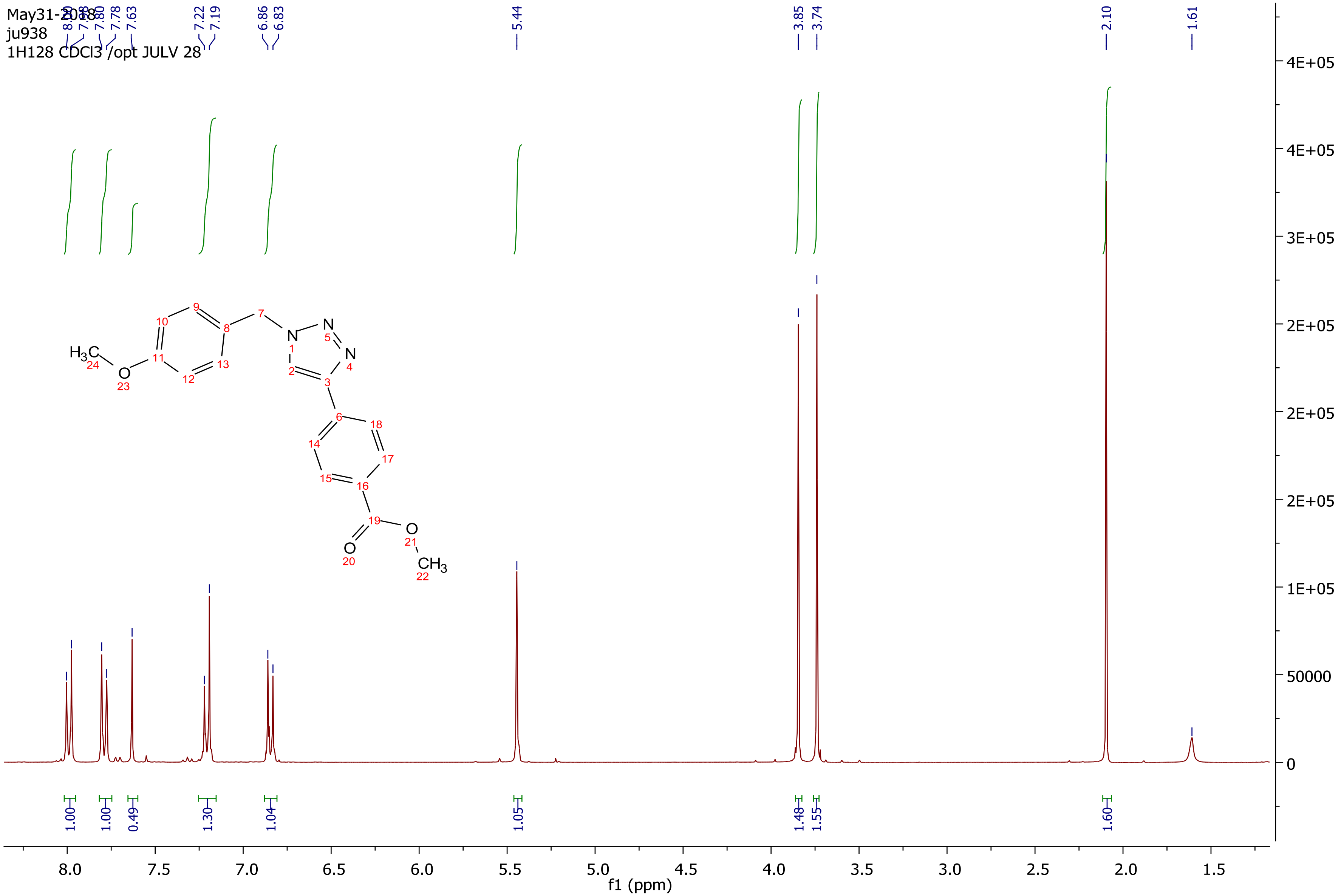
May31-2018
ju938
1H128 CDCl3 /opt JULV 28
8.00
7.98
7.80
7.78
7.63
7.22
7.19
6.86
6.83
5.44
3.85
3.74
2.10
1.61
1.00
1.00
0.49
1.30
1.04
1.05
1.48
1.55
1.60
f1 (ppm)
H3C
O
N
N
N
O
O
CH3

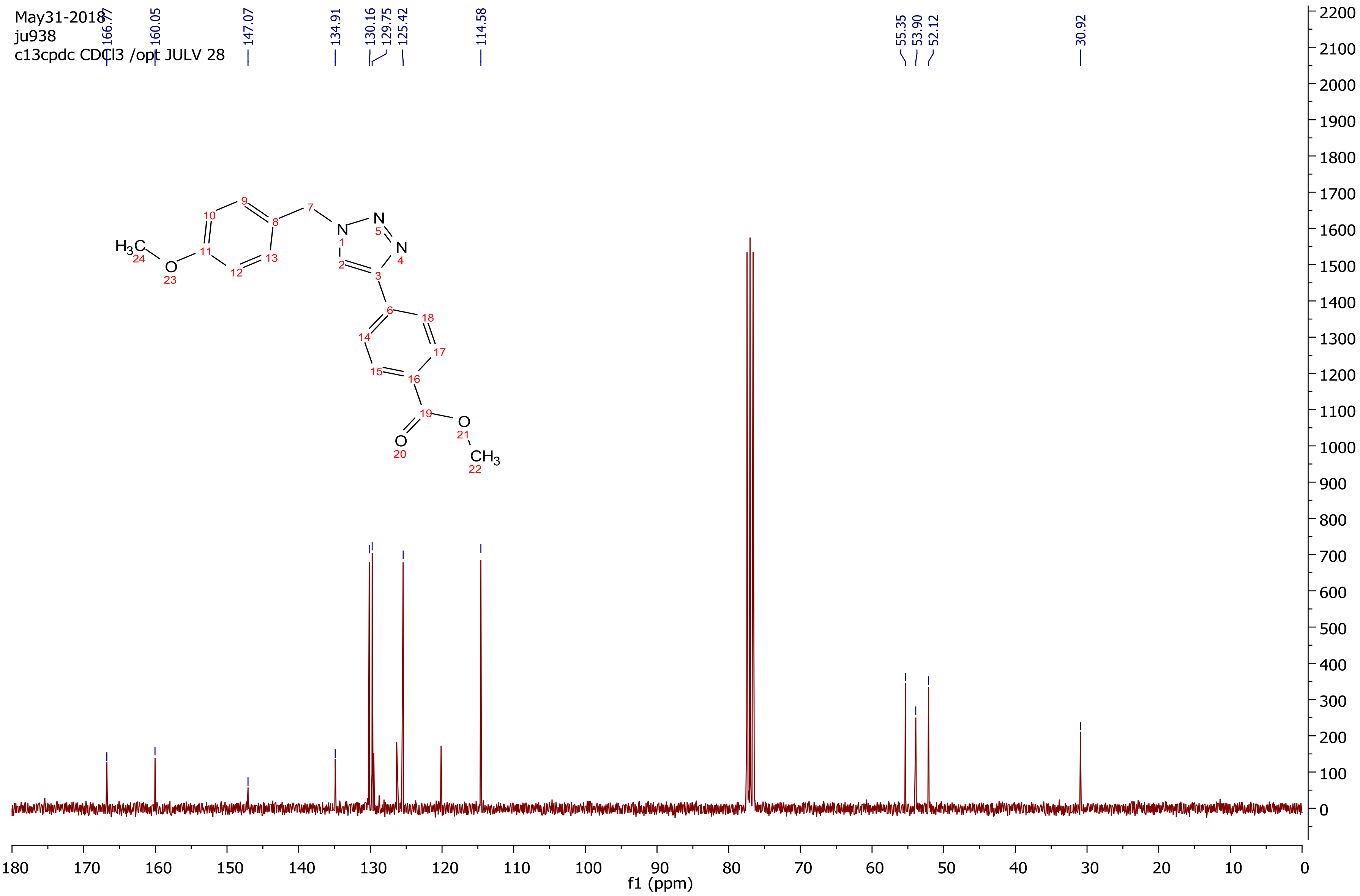
May31-2018
ju938
c13cpdc CDCl3 /opt JULV 28
166.77
160.05
147.07
134.91
130.16
129.75
125.42
114.58
55.35
53.90
52.12
30.92
f1 (ppm)

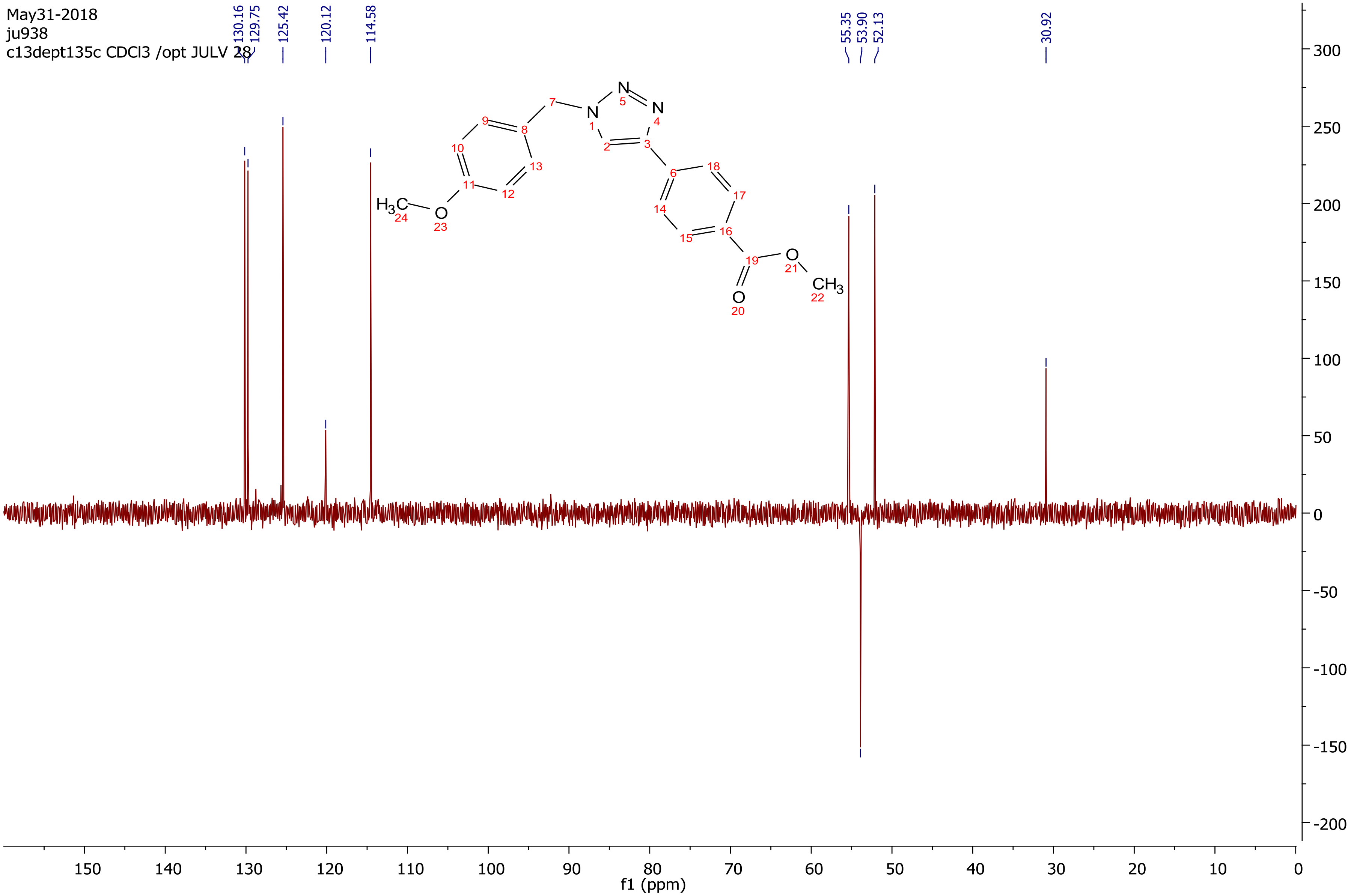

May31-2018
ju938
c13dept135c CDCl3 /opt JULV 28
130.16
129.75
125.42
120.12
114.58
55.35
53.90
52.13
30.92
f1 (ppm)

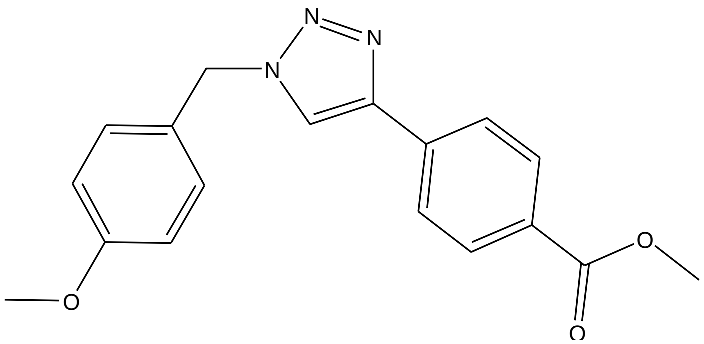

N
N
N
O
O
O

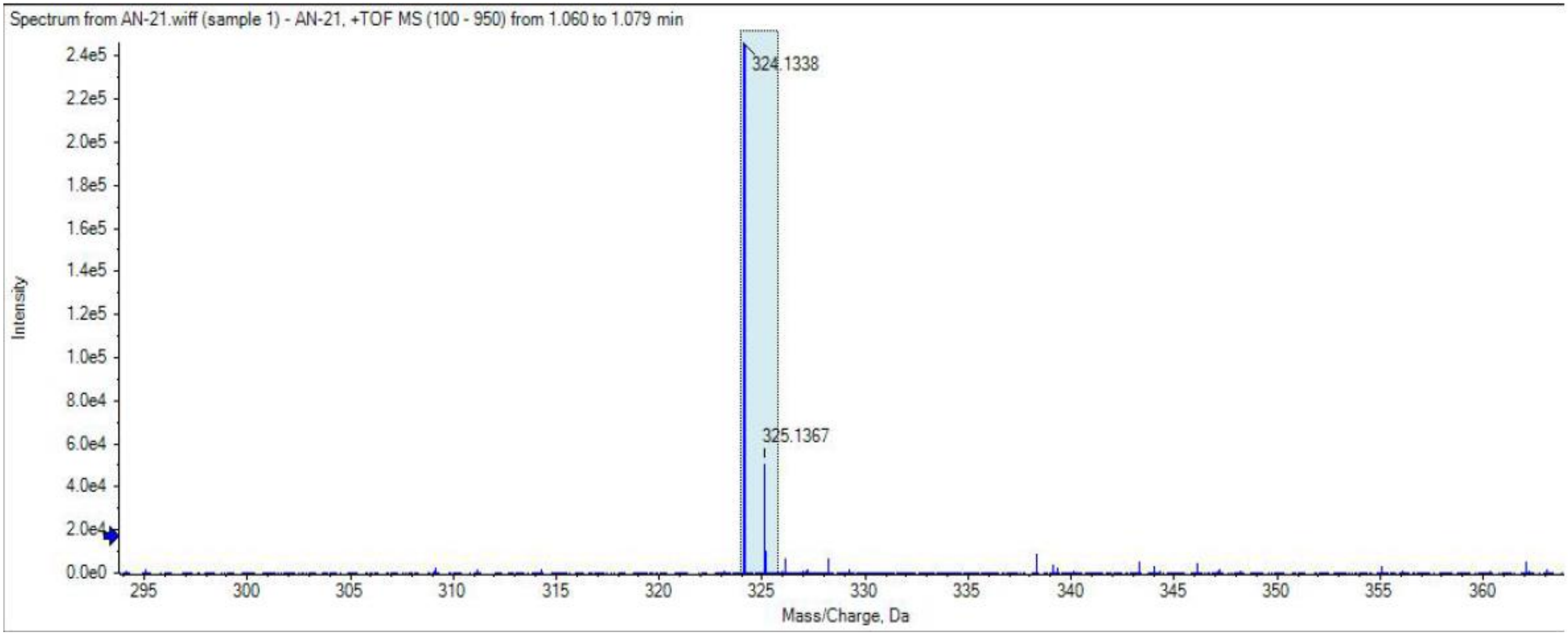

Spectrum from AN-21.wiff (sample 1) - AN-21, +TOF MS (100 - 950) from 1.060 to 1.079 min
Intensity
2.4e5
2.2e5
2.0e5
1.8e5
1.6e5
1.4e5
1.2e5
1.0e5
8.0e4
6.0e4
4.0e4
2.0e4
0.0e0
324.1338
325.1367
295
300
305
310
315
320
325
330
335
340
345
350
355
360
Mass/Charge, Da

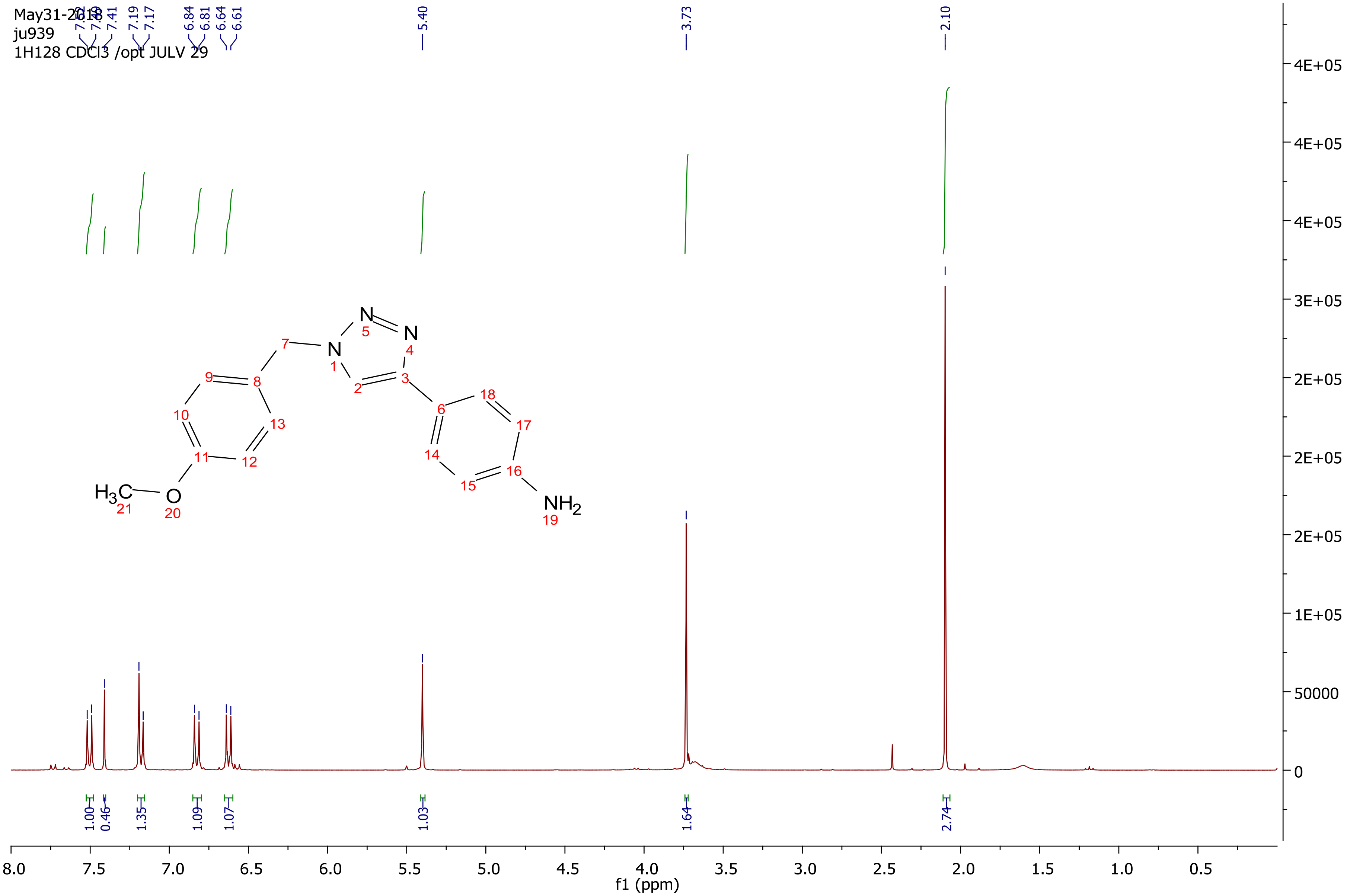
May31-2018
ju939
1H128 CDCl3 /opt JULV 29
7.41
7.19
7.17
6.84
6.81
6.64
6.61
5.40
3.73
2.10
H3C
O
N
N
N
NH2
1.00
0.46
1.35
1.09
1.07
1.03
1.64
2.74
f1 (ppm)

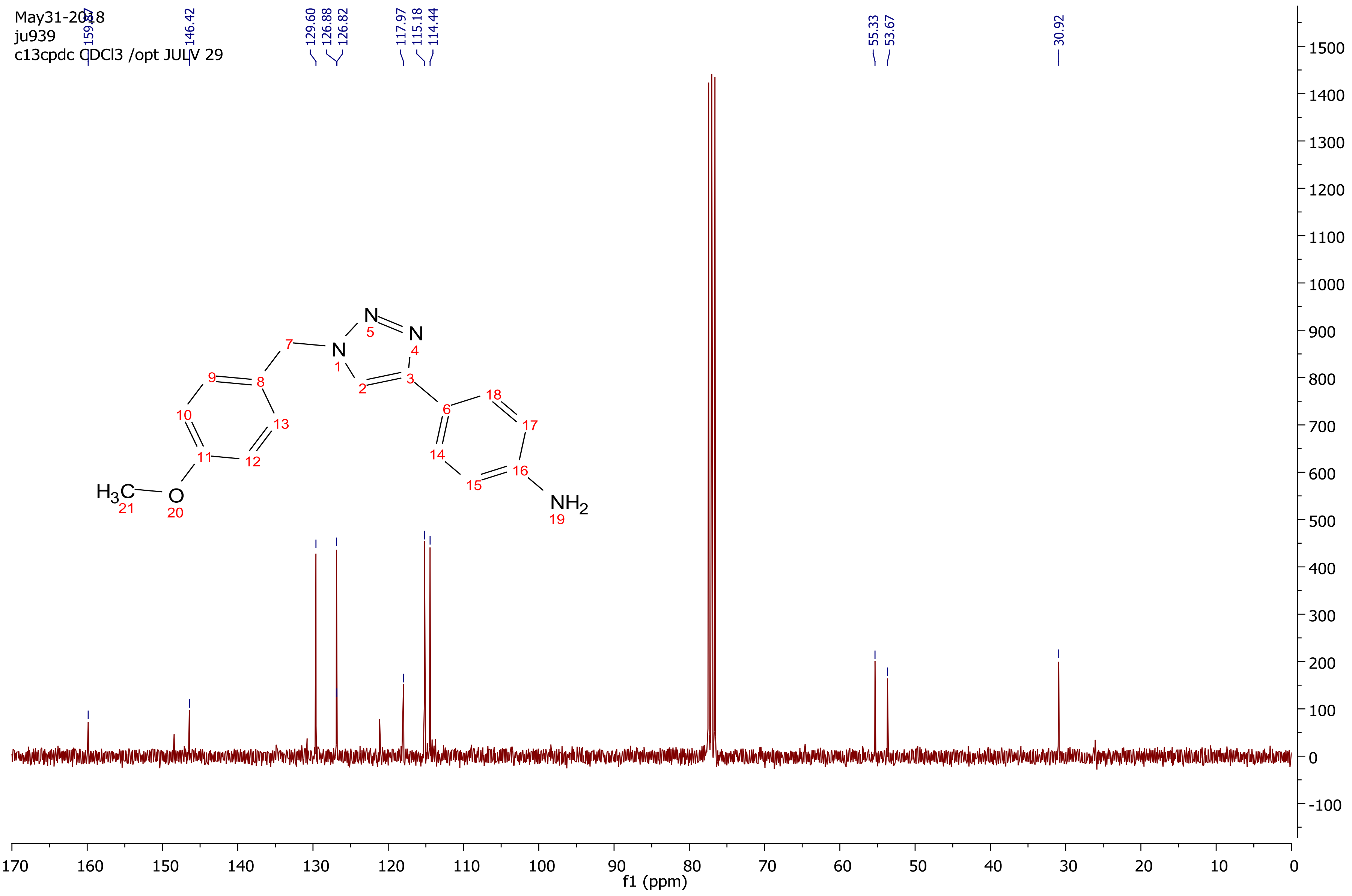

May31-2018
ju939
c13cpdc CDCl3 /opt JULY 29
159.97
146.42
129.60
126.88
126.82
117.97
115.18
114.44
55.33
53.67
30.92
f1 (ppm)

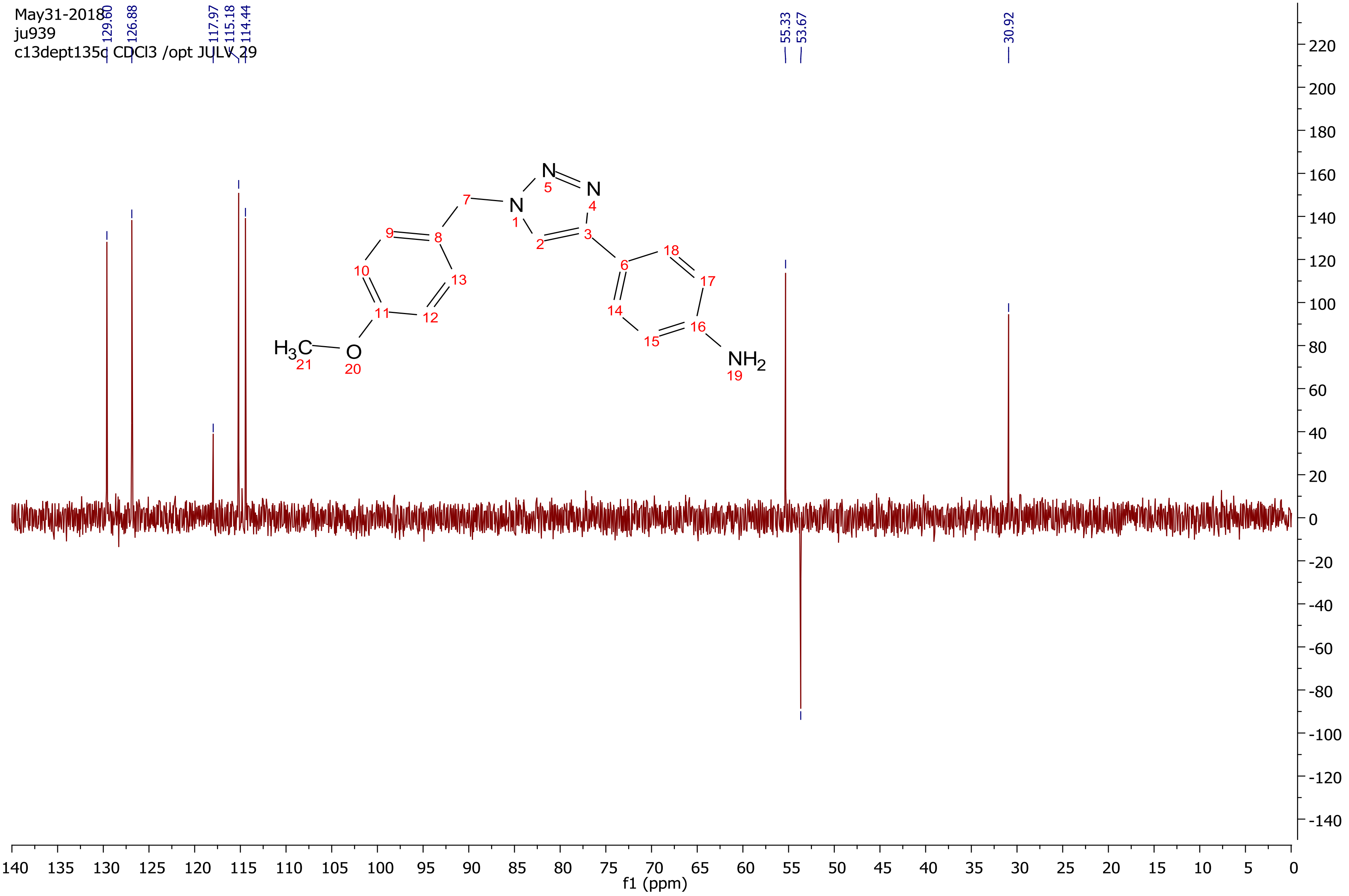
May31-2018
ju939
c13dept135c CDCl3 /opt JULV 29
129.60
126.88
117.97
115.18
114.44
55.33
53.67
30.92
f1 (ppm)
H3C
O
N
NH2

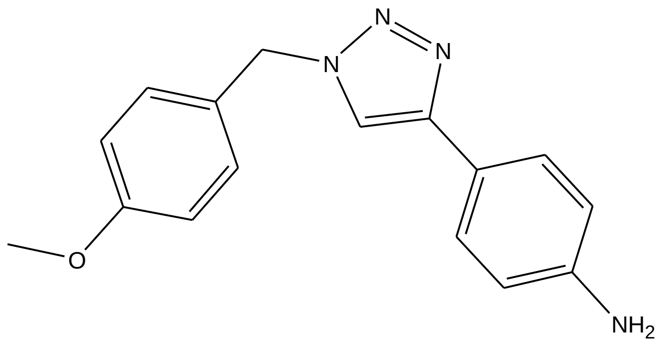
N
N
N
O
NH2

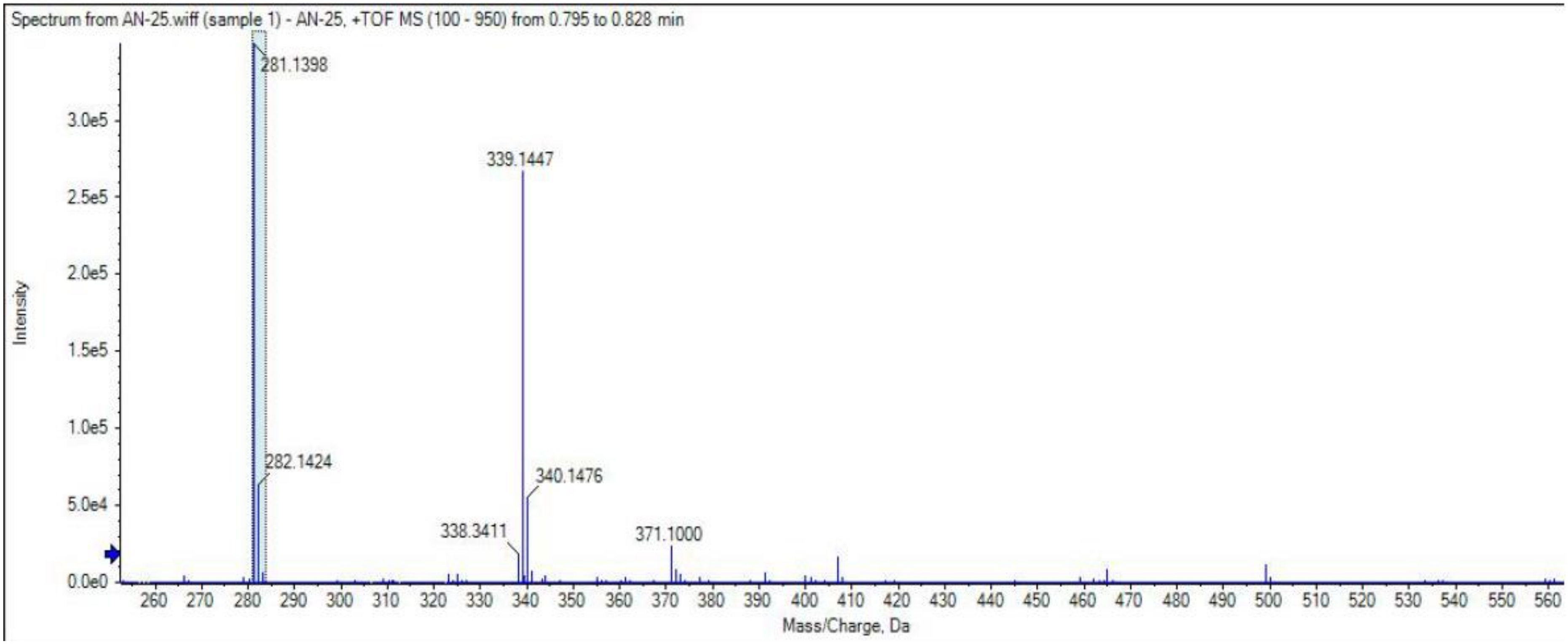
Spectrum from AN-25.wiff (sample 1) - AN-25, +TOF MS (100 - 950) from 0.795 to 0.828 min
281.1398
339.1447
282.1424
340.1476
338.3411
371.1000
Intensity
Mass/Charge, Da
3.0e5
2.5e5
2.0e5
1.5e5
1.0e5
5.0e4
0.0e0
260 270 280 290 300 310 320 330 340 350 360 370 380 390 400 410 420 430 440 450 460 470 480 490 500 510 520 530 540 550 560
